# CD22 Exon 12 Deletion as an Independent Predictor of Poor Treatment Outcomes in B-ALL

**DOI:** 10.3390/cancers15051599

**Published:** 2023-03-04

**Authors:** Sanjive Qazi, Fatih M. Uckun

**Affiliations:** 1 Ares Pharmaceuticals, Saint Paul, MN 55110, USA; 2 Division of Hematology-Oncology, Department of Pediatrics and Norris Comprehensive Cancer Center, University of Southern California Keck School of Medicine (USC KSOM), Los Angeles, CA 90027, USA

**Keywords:** B-ALL, CD22, mRNA, relapse, aberrant splicing, SIGLEC-2

## Abstract

**Simple Summary:**

We previously reported that cancer cells from the most common type of childhood cancer, namely, a form of acute leukemia known as B-ALL, are characterized by an abnormality known as CD22 exon 12 deletion. The purpose of the present study was to evaluate the clinical significance of the CD22 exon 12 deletion. Our findings provide the first evidence that CD22 exon 12 deletion is associated with a poor treatment outcome in B-ALL. The reported results also support the notion that the further evaluation of the clinical potential of new strategies targeting this abnormality in B-ALL is warranted.

**Abstract:**

We previously reported a splicing defect (CD22ΔE12) associated with the deletion of exon 12 of the inhibitory co-receptor CD22 (Siglec-2) in leukemia cells from patients with CD19^+^ B-precursor acute lymphoblastic leukemia (B-ALL). CD22ΔE12 causes a truncating frameshift mutation and yields a dysfunctional CD22 protein that lacks most of the cytoplasmic domain required for its inhibitory function, and it is associated with aggressive in vivo growth of human B-ALL cells in mouse xenograft models. Although CD22ΔE12 with selective reduction of CD22 exon 12 (CD22E12) levels was detected in a high percentage of newly diagnosed as well as relapsed B-ALL patients, its clinical significance remains unknown. We hypothesized that B-ALL patients with very low levels of wildtype CD22 would exhibit a more aggressive disease with a worse prognosis because the missing inhibitory function of the truncated CD22 molecules could not be adequately compensated by competing wildtype CD22. Here, we demonstrate that newly diagnosed B-ALL patients with very low levels of residual wildtype CD22 (“CD22E12^low^”), as measured by RNAseq-based CD22E12 mRNA levels, have significantly worse leukemia-free survival (LFS) as well as overall survival (OS) than other B-ALL patients. CD22E12^low^ status was identified as a poor prognostic indicator in both univariate and multivariate Cox proportional hazards models. CD22E12^low^ status at presentation shows clinical potential as a poor prognostic biomarker that may guide the early allocation of risk-adjusted, patient-tailored treatment regimens and refine risk classification in high-risk B-ALL.

## 1. Introduction

The inhibitory B-cell co-receptor CD22 (Siglec-2) regulates several signaling pathways related to the proliferation and survival of B-lineage lymphoid cells [1,2,3,4]. The inhibitory function of CD22 is mediated by the interactions of its cytoplasmic domain with the protein tyrosine phosphatase SHP-1 [5,6]. Any CD22 mutation impairing or preventing this protein–protein interaction would hamper the inhibitory function of CD22 and thereby result in abnormally augmented proliferation as well as prolonged survival of B-lineage lymphoid cells. We previously reported a splicing defect associated with the deletion of CD22 Exon 12 (CD22E12) in leukemia cells from patients with CD19^+^ B-precursor acute lymphoblastic leukemia (B-ALL) [7]. We demonstrated that this CD22E12 deletion (“CD22ΔE12”) causes a truncating frameshift mutation and yields a C-terminal truncated dysfunctional CD22 protein that lacks most of its cytoplasmic domain, including the signal transduction elements that are required for the interaction of CD22 with SHP-1 [7]. CD22ΔE12 is associated with aggressive in vivo growth of human B-ALL cells in mouse xenograft models [7]. Furthermore, forced overexpression of the mutant human CD22ΔE12 in transgenic mice caused fatal B-ALL, demonstrating that CD22ΔE12 alone may be sufficient as a driver lesion for the leukemic transformation and aggressive in vivo growth of BCPs [7,8]. We previously reported that CD22E12 mRNA expression levels, as measured via multiprobe transcriptome profiling using the microarray platform, are selectively and significantly reduced in B-ALL cells with the CD22ΔE12 splicing defect [8,9,10,11,12,13,14]. Using Western blot analysis and RT-PCR, we demonstrated that CD22 exon 12 deletion is not observed in normal human pro-B and pre-pre-B cells [7,10]. Further, our comparison of matched-pair diagnostic vs. post-induction remission bone marrow specimens from B-ALL patients showed a marked reduction of CD22ΔE12 mRNA levels after chemotherapy. These findings demonstrate that normal hematopoietic cells in the remission bone marrow of CD22ΔE12^+^ B-ALL patients do not express the aberrant CD22ΔE12 mRNA associated with the CD22ΔE12 genetic defect [12].

Although CD22ΔE12 was detected in a high percentage of newly diagnosed and relapsed B-ALL patients [7,8,9,10,11,12,13,14], its clinical significance has yet to be deciphered. The purpose of the present study was to evaluate the clinical prognostic significance of CD22ΔE12 in B-ALL. We postulated that residual wildtype CD22 with undeleted exon 12 could compensate for the missing inhibitory function of the truncated CD22 molecules and thereby mitigate the net effect of the CD22ΔE12 splicing defect on B-ALL cells [12,13]. Indeed, we previously demonstrated that lentiviral-based overexpression of full-length wildtype CD22 in CD22ΔE12-positive B-ALL cells virtually abrogates their clonogenic growth in vitro [13]. We hypothesized that the missing inhibitory function of the truncated CD22 molecules could not be adequately compensated by very low levels of wildtype exon 12-containing CD22. Therefore, we set out to test the hypothesis that B-ALL patients with very low levels of CD22E12 (“CD22E12^low^”), as measured by RNAseq-based CD22E12 mRNA levels, would have a worse prognosis than other B-ALL patients. Our findings provide unprecedented evidence that CD22E12^low^ B-ALL patients have worse leukemia-free survival (LFS) and overall survival (OS) outcomes than other B-ALL patients. CD22E12^low^ status was identified as a poor prognostic indicator in univariate and multivariate Cox proportional hazards models.

## 2. Materials and Methods

### 2.1. Comparative Analysis of the Expression Levels of CD22 Exons 11−14 in Primary Leukemia Cells from Newly Diagnosed Pediatric Patients with B-All and Normal Hematopoietic Cells from Non-Leukemic Controls Using a Human Genome Expression Microarray Platform for Transcriptome Profiling

We used the publicly available archived gene expression profiling datasets GSE13159, GSE11877, and GSE13351, which were generated in the GeneChip Human Genome U133 Plus 2.0 Array platform (Thermo Fischer Scientific, Waltham, MA, USA), to examine the relative expression levels of CD22 exons 11−14 in primary leukemia cells from 421 newly diagnosed pediatric B-ALL patients. The B-ALL patient population included Ph-like B-ALL patients (*n* = 154; GSE11877 and GSE13351); TCF3-PBX1^+^/E2A-PBX1 B-ALL patients (*n* = 25; GSE11877 and GSE13351); KMT2A/MLL-R+ B-ALL patients (*n* = 25; GSE11877 and GSE13351); BCR-ABL/Ph^+^ ALL patients (*n* = 123; GSE13159 and GSE13351); other B-ALL patients (*n* = 94; GSE11877 and GSE13351). BLAT analysis of CD22 probe sequences for the Affymetrix probe set 217422_s_at (9 probes covering exons 11–14; Human Genome U133 Plus 2.0 Array platform) that were mapped onto specific CD22 exons was visualized using the UCSC genome browser. Expression of each probe was log_2_-transformed and median-centered across all probes in each sample. Similarly, the expression levels of CD22E11, CD22E13, and CD22E14 were estimated by determining the mean expression values for 6 probes mapped to these CD22 exons in B-ALL samples mean-centered to the corresponding expression values in non-leukemic CON samples. The perfect match (PM) signal value for each probe was background-corrected, robust multiarray analysis (RMA)-normalized, log_2_-transformed, and median-centered across all probes in each sample. The expression level of CD22E12 in the B-ALL samples (*n* = 421) was estimated from the mean signal value for the 3 CD22E12 probes, namely HG-U133_PLUS_2:217422_S_AT_7 aligned to chr19:35836590-35836614, HG-U133_PLUS_2:217422_S_AT_6 aligned to chr19:35836566-35836590, HG-U133_PLUS_2:217422_S_AT_5 aligned to chr19:35836535-35836559, and mean-centered to the mean signal value for the same probes in non-leukemic control (CON) samples (*n* = 74, GSE13159). A CD22E12 index was calculated by subtracting the mean expression values for the 6 probes for CD22E11, CD22E13, CD22E14 in the 217422_s_at probe set, namely, HG-U133_PLUS_2:217422_S_AT_11 aligned to chr19: 35837525-35837549/CD22E14), HG-U133_PLUS_2:217422_S_AT_10 aligned to chr19: 35837478-35837502/CD22E14, HG-U133_PLUS_2:217422_S_AT_8 aligned to chr19: 35837100-35837124/CD22E13), HG U133_PLUS_2:217422_S_AT_9 aligned to chr19: 35837117-35837139/CD22E13), HG-U133_PLUS_2:217422_S_AT_4 aligned to chr19:35835979-35836003/CD22E11, and HG-U133_PLUS_2:217422_S_AT_3 aligned to chr19:35835811-35835960/CD22E11, from the expression values for the 3 CD22E12 probes, as previously reported [8,9,10,11,12,13,14].

Expression levels for CD22E12 versus the mean of CD22E11, CD22E13, and CD22E14 were visualized using density graphs (fitted using a Gaussian smoothing kernel density estimation) superimposed on histograms of the CD22E12 index values and were plotted for CON and B-ALL samples (ggplot2_3.3.5 R package).

### 2.2. Detection of CD22ΔE12 mRNA in B-ALL Leukemia Samples via Real-Time Quantitative RT-PCR

Cellular RNA was extracted from Ficoll–Hypaque-separated leukemia cells of 12 de-identified pediatric patients with newly diagnosed high-risk B-ALL and 12 de-identified pediatric patients with newly diagnosed standard-risk ALL using the Qiagen RNeasy Mini Kit (Cat# 74104, Qiagen, Valencia, CA). The secondary use of de-identified leukemia cells for subsequent laboratory studies did not meet the definition of human subject research per 45 CFR 46.102 (d and f) because it did not include identifiable private information, and it was approved by the IRB (CCI) at the Children’s Hospital Los Angeles (CHLA) (Protocol #’ CCI-09-00304 and CCI-10−00141; Human Subject Assurance Number: FWA0001914). One-step real-time quantitative (q) RT-PCR was performed using the One-Step PrimeScript RT-PCR kit (Cat. # RR064B, Takara/Clontech, Mountain View, CA) and the Applied Biosystems 7900HT Fast Real-Time PCR System housed in the CHLA/USC Stem Cell Core Facility to compare the expression levels of the CD22ΔE12 mRNA in pediatric B-ALL samples, as previously described in detail [12]. The PCR primer pair (viz.: forward primer E11-F2: 5′-CAGCGGCCAGAGCTTCTT-3′ and reverse primer E13-R2: 5′-GCGCTTGTGCAATGCTGAA-3′) was selected to amplify a 113-bp fragment spanning from Exon 11 to Exon 13 of the human CD22ΔE12 cDNA. The amplified fragment was then specifically annealed to a pre-mixed oligo DNA probe (5′-TGTGAGGAATAAAAAGAGATGCAGAGTCC-3′) conjugated with 5′ FAM reporter and 3′BHQ quencher on the CD22ΔE12-specific unique junction region between exon 11 and exon 13. The FAM reporter fluorescence intensity was recorded by applying the sequence detection system of the real-time qPCR system, expressed as threshold cycle threshold (Ct) value for quantification, as reported [12]. Each sample was also subjected to a qRT-PCR reaction for the housekeeping gene beta (β)-actin with a primer set amplifying a 234-bp region at the junction between exon 4 and exon 5 of the human β-actin gene for normalization of the Ct values, as previously reported [12]. The results were visualized in box plots superimposed with a kernel density plot showing the peaks, median, and inter-quartile range in the numerical distribution of the data (ggplot2_3.3.5 R package).

### 2.3. Data Normalization for Exon-Level CD22 Gene Expression Data Derived from Primary B-ALL Cells

We downloaded the RNAseq data from the Therapeutically Applicable Research to Generate Effective Treatments (TARGET) program (https://target-data.nci.nih.gov/Public/ALL/mRNA-seq/Phase2/L3/expression/BCCA/ accessed on 28 January 2022). Summary files were named using a coding system specific to Office of Cancer Genomics (OCG) characterization programs (https://ocg.cancer.gov/sites/default/files/OCG-Project-Codes_Tissues-and-Samples_03-02-2021.pdf accessed on 28 January 2022), allowing for the identification of data files that contained exon-level mRNA expression levels for each sample. These data files reported the exon locations for the CD22 gene (Human (GRCh37/hg19) build), raw read counts, length of exons, and reads per kilobase million (RPKM) values. Read count, alignment, and within-sample-level normalization of mRNA expression levels were detailed in previous study reports that contributed to the data deposited in the TARGET repository (4; summarized in https://ocg.cancer.gov/programs/target/target-methods#3202 (accessed on 28 September 2022)). Briefly, exon-level quantification was performed by aligning Illumina paired-end RNA sequencing reads (fasta files) to GRCh37-lite genome-plus-junctions and exon–exon junction sequences, whereby the corresponding coordinates were based on annotations of transcripts in the Ensembl (v59) reference using BWA version 0.5.7. Mapped reads between these junction regions were positioned according to the reference genome (GRCh37/hg19) build). The raw count reports the number of reads that overlapped each of the CD22 exon junctions in each sample. The corresponding data files with exon-level quantification for the CD22 exons 11, 12, 13, and 14 were manually downloaded from the TARGET repository. The exon locations, which were all on the positive strand, were as follows: CD22E11:35835954-35836029; CD22E12:35836505-35836623; CD22E13:35837054-35837138; CD22E14:35837469-35838258. We utilized the normalized RPKM metric for the exon-level quantification of CD22 mRNA. In this summarization, the total read count in a sample was divided by 10^6^ to obtain the scaling factor. The read counts were divided by the scaling factor to normalize the read counts for sequencing depth (reads per million (RPM)). The RPM value was then divided by the length of the exon in kilobases to calculate RPKM. We calculated the CD22E12 expression to measure the relative level of CD22E12 expression compared to the mean level of expression of 3 surrounding exons with similar RPKM values, namely, CD22E11, CD22E13, and CD22E14. Mean RPKM values were used for cases with duplicate samples. The sample information was obtained from annotation files deposited on the TARGET website: TARGET_ALL_SampleMatrix_Phase2_Validation_20190606.xlsx and TARGET_ALL_SampleMatrix_Phase2_Discovery_20190606.xlsx. The archived database contained the data from the B-ALL patients (*n* = 141) that were used in our analyses, including 90 pediatric or young adult patients with NCI high-risk ALL treated in the Children’s Oncology Group (COG) studies P9906 [14] or AALL0232 [15,16,17] and 51 pediatric patients with standard risk ALL treated in COG study AALL0331 [18,19]. Within the 141-patient RNA-seq subset, 89 high-risk patients were treated in the COG study AALL0232 [15,16,17], and 51 standard-risk patients were treated in the COG study AALL0331 [18,19]. CD22-targeting therapies were not part of these protocols. No patient received inotuzumab-ozogamicin. Treatment details for the AALL0232 and AALL0331 protocols are described in detail and can be accessed via https://clinicaltrials.gov/ct2/show/NCT00075725 and https://clinicaltrials.gov/ct2/show/NCT00103285, respectively. In brief, AALL0232 was a randomized, multicenter study (see https://clinicaltrials.gov/ct2/show/results/NCT00075725 for treatment details, accessed on 1 January 2023). Patients were stratified according to early response (slow early response (SER) vs. rapid early response (RER)). For Induction therapy, patients were randomized to 1 of 4 treatment arms: (i) ARM I: patients received cytarabine intrathecally (IT) on day 1, vincristine intravenously (IV) and daunorubicin IV on days 1, 8, 15, and 22, dexamethasone orally (PO) or IV twice daily (BID) on days 1–14, methotrexate (MTX) IT on days 8 and 29*, and pegaspargase intramuscularly (IM) once on day 4, 5, or 6. Patients with CNS3 disease (WBC > 5/mL in cerebrospinal fluid and positive for blasts on cytospin) also received MTX IT on days 15 and 22. (ii) ARM II: patients received induction therapy as in ARM I. (iii) ARM III: patients received cytarabine, vincristine, daunorubicin, and pegaspargase as in ARM I. Patients also received prednisone PO or IV BID on days 1–28 and MTX IT on days 8 and 29. (iv) ARM IV: patients received induction therapy as in ARM III. Patients in all arms were evaluated at day 29 of induction therapy. Patients with M3 disease were removed from the study. Patients with M1 disease and less than 1% minimal residual disease (MRD) proceeded to consolidation therapy beginning on day 36. Patients with M2 disease or with MI disease and at least 1% MRD received extended induction therapy for 2 additional weeks. Patients with SER disease and MLL rearrangements were removed from the study. For extended induction therapy, patients continued to receive therapy according to the arm to which they were originally randomized: ARMS I and II: patients received dexamethasone PO or IV BID on days 1–14, vincristine IV on days 1 and 8, daunorubicin IV on day 1, and pegaspargase IM on day 4, 5, or 6 and were then reevaluated; ARMS III and IV: patients received prednisone PO or IV BID on days 1–14 and vincristine, daunorubicin, and pegaspargase as in Arms I and II, and they were then reevaluated. Patients in all arms who had M1 disease and less than 1% MRD after extended induction proceeded to consolidation therapy and continued as SER patients. All other patients were removed from the study. For consolidation therapy, all patients received cyclophosphamide IV over 30 min on days 1 and 29, cytarabine IV or subcutaneously (SC) on days 1–4, 8–11, 29–32, and 36–39, mercaptopurine (MP) PO on days 1–14 and 29–42, vincristine IV on days 15, 22, 43, and 50, pegaspargase IM on days 15 and 43, and MTX IT on days 1, 8, 15, and 22. Patients with testicular disease also received radiotherapy to the testes. Patients with CNS3 disease received MTX on days 1 and 8 only. For interim maintenance therapy 1, patients continued to receive treatment according to the arm to which they were originally randomized: (i) ARM I: (escalating-dose MTX) patients received vincristine IV and escalating-dose MTX IV on days 1, 11, 21, 31, and 41, pegaspargase IM on days 2 and 22, and MTX IT on days 1 and 21. (ii) ARM II: (high-dose MTX) patients received vincristine IV and high-dose methotrexate IV over 24 h on days 1, 15, 29, and 43, MP PO on days 1–56, and IT MTX on days 1 and 29. Patients also received leucovorin calcium IV every 6 h for at least 3 doses, beginning 42 h after start of each MTX infusion. (iii) ARM III: (escalating-dose MTX) patients received interim maintenance 1 therapy as in ARM I. (iv) ARM IV: (high-dose MTX) patients received interim maintenance therapy as in ARM II. For delayed intensification therapy 1, all patients received vincristine IV on days 1, 8, 15, 43, and 50, dexamethasone PO or IV BID on days 1 to 21 for patients ages 1 to 12 OR on days 1–7 and 15–21 for patients ages 13 and over, doxorubicin IV on days 1, 8, and 15, pegaspargase IM on day 4, 5, or 6 as well as day 43, cyclophosphamide IV over 30 min on day 29, cytarabine IV or SC on days 30–33 and 37–40, thioguanine PO on days 29–42, and MTX IT on days 1, 29, and 36. After delayed intensification I, SER patients proceeded to interim maintenance 2 and delayed intensification 2. RER patients proceeded directly to maintenance. For interim maintenance therapy 2, all patients received vincristine IV and MTX IV on days 1, 11, 21, 31, and 41, pegaspargase IM on days 2 and 22, and MTX IT on days 1 and 21. Patients then proceeded to delayed intensification 2. For delayed intensification therapy 2, all patients received therapy as in delayed intensification 2, ARM I. CNS3 patients also received radiotherapy for 3–10 days, beginning on day 29. All other SER patients, patients with MLL rearrangements, and some patients pretreated with steroids (>48 h within the week prior to diagnosis) received prophylactic cranial radiotherapy (CRT) for 8 days, beginning on day 29. Patients then proceeded to maintenance therapy. For maintenance therapy, all patients received vincristine IV on days 1, 29, and 57, dexamethasone PO BID on days 1–5, 29–33, and 57–61, MP PO on days 1–84, MTX IT on day 1*, and MTX PO on days 1, 8, 15, 22, 29, 36, 43, 50, 57, 64, 71, and 78. RER patients (who did not undergo CRT) also received MTX IT on day 29 for maintenance courses 1–4. In all arms, maintenance therapy was repeated every 12 weeks until the total duration of therapy was 2 years from the start of interim maintenance 1 for female patients and 3 years from the start of interim maintenance 1 for male patients. Patients with testicular disease could receive testicular radiotherapy for 8 days during one of the first 3 courses of maintenance therapy. Patients were followed monthly for 1 year, every 2 months for 1 year, every 3 months for 1 year, every 6 months for 1 year, and then annually thereafter. In the AALL0331 study, which tested whether intensified postinduction therapy that improves survival in children with high-risk B-cell acute lymphoblastic leukemia (ALL) would also improve outcomes for those with standard-risk (SR) ALL, patients received a 3-drug induction with IT cytarabine on day 1; weekly IV vincristine for 4 doses; oral dexamethasone for 28 days; 1 dose of intramuscular PEG on day 4, 5, or 6; IT MTX for 2 to 4 doses (see https://www.slideshare.net/AlfredYeung2/aall0331-protocol, accessed on 28 September 2022). Bone marrow (BM) aspiration was performed on days 8 and 15 (if the day-8 marrow was M2/M3) to determine the hematologic response by examining morphology. MRD testing was performed at day 29 using flow cytometry. Rapid early response (RER) was defined as <5% BM blasts (M1) by day 15 based on local morphologic interpretation and an M1 BM with MRD < 0.1% at day 29. Slow early responders (SERs) had an M2 (5–25%) or M3 (>25%) BM on day 15 and/or positive MRD (≥0.1% to <1%) at day 29. For patients with M3 marrow at day 29, induction was considered to have failed, and they were taken off protocol therapy. Patients with an M2 marrow or an M1 marrow with MRD ≥ 1% at day 29 received an extended induction with 2 additional weeks of therapy and continued on study as SERs if they achieved day-43 M1 marrow and MRD < 1%. Those not achieving these criteria were removed from protocol therapy. All patients were initially required to have central testing for triple trisomies of chromosomes 4, 10, and 17 (TT) and BCR-ABL1, ETV6-RUNX1, or KMT2A rearrangement (KMT2A-R) using fluorescence in situ hybridization. After induction, patients were classified into 1 of 3 risk groups: SR low (RER, CNS1, and favorable cytogenetics of TT or ETV6-RUNX1 fusion), SR average (no unfavorable genetic features (BCR-ABL1, KMT2A-R, or hypodiploidy with < 44 chromosomes), RER, and CNS1 or 2 (patients with favorable genetics who were RERs and CNS2)), or SR high (KMT2A-R and RER, anyone with CNS3 at diagnosis, and SERs by morphology or MRD). Patients with overt testicular leukemia were not eligible. Patients with BCR-ABL1 fusion or hypodiploidy did not continue to receive therapy after induction. Patients with SR-low disease were randomly assigned to regimens with or without 4 additional doses of PEG at approximately 3-week intervals, with the backbone of standard consolidation (SC) and initially standard IM with weekly oral MTX. All patients with SR-low disease received standard DI and maintenance. Patients with SR-average disease were randomly assigned initially in a 2-by-2 factorial design to 1 of 4 treatment regimens: SS (SC and standard IM and DI), SA (SC with intensified IM (AIM) and DI (ADI)), IS (intensified consolidation (IC) and standard IM/DI), and IA (IC, AIM, and ADI). IC was identical to the augmented Berlin–Frankfurt–Münster (BFM) consolidation used in COG AALL0232, AIM was identical to the Capizzi-style escalating IV MTX and PEG, and DI incorporated additional doses of VCR and PEG, as used in AALL0232. Patients with SR-high disease were nonrandomly assigned to receive full augmented BFM therapy, as administered in CCG 1961, including IC, AIM1, ADI1, AIM2, ADI2, and maintenance. CNS3 patients underwent 18-Gy cranial irradiation. In all arms of AALL0331, the length of therapy from the start of IM1 was 2 years for girls and 3 years for boys. In 2008, the results of CCG 1991 became available, showing that escalating IV MTX without leucovorin rescue improved EFS compared to standard IM with oral MTX. AALL0331 amendment 2C replaced the oral MTX IM phase with escalating IV MTX for all patients with SR-average disease. All patients with SR-average disease received IM with IV escalating MTX and standard DI. This amendment also changed dexamethasone administration in DI to discontinuous dosing (days 1–7 and 15–21) rather than a continuous schedule (days 1–21) because of increased rates of osteonecrosis in AALL0232 with continuous dexamethasone during DI. When the results of AALL0232 demonstrated that high-dose MTX was superior to Capizzi MTX, amendment 7 (May 2011) changed therapy for patients with SR-high disease who had not yet begun maintenance cycle 2. They then received an additional IM phase with high-dose MTX. 26 patients out of the 1126-patient complete set (2.3%) of the TARGET database for B-ALL received a transplant compared to 6 patients out of 141 RNAseq subset (4.3%) who received a transplant (*p* = 0.2). Within the 141-patient RNAseq subset, none of the 21 CD22E12^low^ patients received transplants compared to 6 out of 120 (5%) remaining patients from the RNAseq subset (Fisher’s Exact Test, *p* = 0.6). No information is available regarding the type or timing of the transplants. No patient received inotuzumab-ozogamicin. We excluded the data from 43 additional patients with an unknown cell of origin/immunophenotype information for leukemia cells. In addition, data files reporting the RPKM metric for CD22 gene exon-level quantification of mRNA for CD22 exons 1-4 were downloaded from the TARGET phase 2 project (https://target-data.nci.nih.gov/Public/ALL/mRNA-seq/Phase2/L3/, accessed on 28 September 2022). The expression level of each exon was mean-centered to the average RPKM values across exons 1 to 4 (CD22E1–4) for each of the 141 B-ALL patients (RPKM-normalized).

### 2.4. Hierarchical Clustering Analysis to Identify CD22E12^low^ B-ALL Patients

A one-way hierarchical clustering technique was used to organize the RNAseq-based mRNA expression patterns for the CD22 exons 11, 13, and 14 side-by-side with the expression of CD22E12 across 141 B-ALL patients. The expression level of each CD22 exon was mean-centered to the average RPKM values across CD22E11–14 such that patient level and exon-level expression patterns displayed similar expression profiles that were grouped together using the average distance metric (default Euclidean distance and Wards linkage implemented using the heatmap.2 function in the R package gplots_3.1.1). The cluster analysis revealed a subset of patients whose cells exhibited a markedly and selectively reduced expression level of CD22E12 compared to CD22E11, CD22E13, and CD22E14 (“CD22E12^low^ subset”). To confirm the selective reduction of CD22E12 relative to CD22E11 and CD22E13 in CD22E12^low^ patients, the corresponding normalized RPKM expression values were compared utilizing a two-factor ANOVA model: patient grouping and exon ID were used as fixed variables, and patient grouping × exon ID was an interaction term (*p*-values were adjusted for multiple comparisons by controlling the false discovery rate to less than 0.01 (multcomp_1.4-17 and emmeans_1.7.0 packages ran in R version 4.1.2 (1 November 2021) with Rstudio front end (RStudio 2021.09.0 + 351 “Ghost Orchid” Release)). Bar chart graphics were constructed using the ggplot2_3.3.5 R package.

### 2.5. Normalization of Gene Level RNAseq Data Derived from Primary B-ALL Cells

We downloaded gene-level RNAseq data from the TARGET program (https://target-data.nci.nih.gov/Public/ALL/mRNA-seq/Phase2/L3/expression/BCCA/ accessed on 9 July 2022) using the web-scraping utility implemented in R version 4.1.2 (1 November 2021) (rvest_1.0.2 and stringr_1.4.0). These data files reported the Ensembl gene IDs, raw read counts, median length of each gene, and RPKM values. Gene identifications were converted from ensemble IDs to gene symbols using the Bioconductor database org.Hs.eg.db_3.14.0, which was interrogated using the functions provided in AnnotationDbi_1.56.1. We compared the gene level RNAseq data for B-ALL samples that exhibited substantially reduced levels of CD22E12 mRNA (*n* = 21; CD22E12^low^) versus all other B-ALL samples (*n* = 120) using the DESeq2 package (DESeq2_1.34.0) obtained from http://www.bioconductor.org/packages/release/bioc/html/DESeq2.html (accessed on 28 September 2022) and implemented using R version 4.1.2 (1 November 2021) [20]. DESeq2 employs a generalized linear model for each gene that fits raw read counts to negative binomial distribution to calculate mean and variance estimates, whereby the mean is taken as a quantity proportional to the concentration of cDNA fragments from the gene in the sample and scaled by a normalization factor across all samples. The normalization method in the DESeq2 algorithm determines the counts divided by sample-specific size factors calculated from the median ratio of gene counts relative to the geometric mean per gene across all samples that accounts for the sequencing depth and RNA composition of each gene. This method allows for fold change comparisons across treatment groups in the GLM model [21,22,23]. The statistical significance of differences in gene expression levels was assessed by testing the null hypothesis that there is no differential expression across the two sample groups (Log_2_ fold change = 0) using the Wald test [20], reporting the test statistic and *p*-value for each gene. To visualize the gene expression profiles in heatmaps, we calculated the normalized log_2_ values from the RNAseq count data using the statistical package implemented in R, vsn_3.62.0 [24]. This method uses a robust variant of the maximum likelihood estimator for the stochastic model of count data that employs data calibration, accounting for the dependence of variance of mean intensity and variance stabilizing data transformation. Low-count values tend to generate large fold changes; therefore, to calculate a more accurate log_2_ fold change estimate, we applied a shrinkage of the log_2_ fold change estimates toward zero when the read counts were low and variable (“normal” function in the DESeq2 package) [25].

### 2.6. Gene Set Enrichment Analysis (GSEA) for Evaluation of Reactome Pathways in B-ALL Patients with Low CD22E12 Expression

Data files reporting raw read counts determined using gene-level quantification of mRNA were downloaded from the TARGET phase 2 project (https://target-data.nci.nih.gov/Public/ALL/mRNA-seq/Phase2/L3/expression/BCCA/ (accessed on 9 July 2022)). We determined the differential expression of genes comparing patients that exhibited low CD22 exon 12 expression (*n* = 21; CD22E12^low^) with all other patients (*n* = 120) using DESeq2 package (DESeq2_1.34.0 implemented in R). We used GSEA (fgsea_1.20.0 [26] implemented in R) and the rank-ordered Wald statistic for the comparison of the 21 CD22E12^low^ patients versus the remaining 120 patients regarding representation patterns of reactome pathways (reactome.db_1.77.0 obtained from Bioconductor: https://www.bioconductor.org/packages/release/data/annotation/html/reactome.db.html, accessed on 9 July 2022). We focused our initial analysis on pathways grouped under transcription, translation (including mRNA processing, mRNA transport, and post-translational modification), and cell cycle because our previously published cluster analysis of highly enriched gene sets revealed that transcriptional, translational and cell cycle processes were significantly upregulated in all three comparisons of CD22ΔE12 Tg mice compared to BCR-ABL Tg, Eμ-MYC Tg, and WT mice (GSE58874 and GSE58872) [9]. In addition, pathways were analyzed and grouped by signal transduction based on our previously published phosphoproteome data for CD22ΔE12-Tg mice (GSE58873 and GSE 58874). The GSEA evaluated the enrichment score (ES) values, representing observed rankings compared to the expected null distribution calculated from the permutation of gene assignments to the ranking scores. Nominal *p*-values were computed by comparing the tails of the ES scores for observed and permutation-generated null distributions following 100,000 permutations. The significance of the association was assessed using weighted Kolmogorov–Smirnov statistics. In order to compare the differences in gene expression levels across gene sets, normalized enrichment scores (NES) were calculated based on the number of genes in the gene set. Low-expression genes (base mean values calculated in the Dseq2 procedure > 10 normalized counts across all samples) were filtered before GSEA analysis for representation in reactome pathways. This resulted in 22,068 genes that were processed for GSEA, comparing CD22E12^low^ B-ALL patients with all other patients for gene set enrichment in 1256 in reactome pathways (gene sets ranged from 10–100 genes; 100,000 permutations were performed to calculate enrichment scores and associated *p*-values). The expression of significantly affected genes was visualized using heatmaps and dendrograms represented in a cluster figure (R package gplots_3.1.1) depicting normalized expression levels in CD22E12^low^ B-ALL samples mean-centered to all other samples.

### 2.7. Analysis of Treatment Outcomes according to RNAseq-Based CD22E12 mRNA Expression Levels

The outcome data were retrieved from the TARGET clinical annotation files TARGET_ALL_ClinicalData_Phase_II_Discovery_20211118.xlsx and TARGET_ALL_ClinicalData_Phase_II_Validation_20211118.xlsx. The Kaplan–Meier (KM) method, log-rank chi-square test, and the software packages survival_3.2-13, survminer_0.4.9, and survMisc_0.5.5, which were operated in the R environment, were used to compare the treatment outcomes of patients, including time to relapse, relapse-free survival (LFS), and overall survival (OS). Graphical representations of the treatment outcomes were generated using three graph-drawing packages implemented in the R programming environment: dplyr_1.0.7, ggplot2_3.3.5, and ggthemes_4.2.4. We compared the outcomes of CD22E12^low^ patients (*n* = 21) vs. all other patients (*n* = 120) in an effort to evaluate the prognostic significance of the CD22E12^low^ status. The statistical significance of differences in the outcomes of the compared patient subsets was examined using the log-rank chi-square test, and *p*-values less than 0.05 were deemed significant.

### 2.8. Multivariate and Univariate Cox Regression Models to Test for the Independent Effect of CD22E12^low^ Status

Multivariate analysis of the poor prognostic impact of CD22ΔE12 was performed using a Cox proportional hazards model in which we examined if the CD22E12^low^ status observed in 21 of 141 B-ALL patients remained a significant predictor of adverse outcomes after controlling for other patient characteristics of established prognostic significance. We compared the hazard ratios (HR) from the multivariate Cox model with univariate Cox models for each of the prognostic factors used as covariates in the multivariate model. Estimates of the life table HR were calculated using the exponentiated regression coefficient for Cox regression analyses implemented in R (survival_3.2-13 was run with R version 4.1.2 (1 November 2021)). Forest plots were utilized to visualize the HR values obtained in the Cox proportional hazards model (survminer_0.4.9 was run with R version 4.1.2 (1 November 2021)). A total of 141 patients were evaluable in the Cox proportional hazards regression model. Analyses were performed both for all 141 patients as well as for the high-risk subset of 90 patients.

The patient characteristics analyzed in the Cox proportional hazards model included the following: (i) CD22E12^low^ status, (ii) age, (iii) gender, (iv) cytogenetic features including molecular markers, (v) WBC at diagnosis (both linear and categorized as ≥20 × 10^9^/L or < 20 × 10^9^/L), and (vi) measurable residual disease (MRD) burden at the end of induction therapy on day 29 (0% vs. >0%; 0% defined as < 0.001% or <1 × 10^−5^), as measured by using 6-color flow cytometry.

## 3. Results

### 3.1. Interpatient Heterogeneity in Microarray-Based CD22E12 and qRT-PCR-Based CD22ΔE12 mRNA Expression Levels among Newly Diagnosed B-ALL Patients

**Figure 1:**
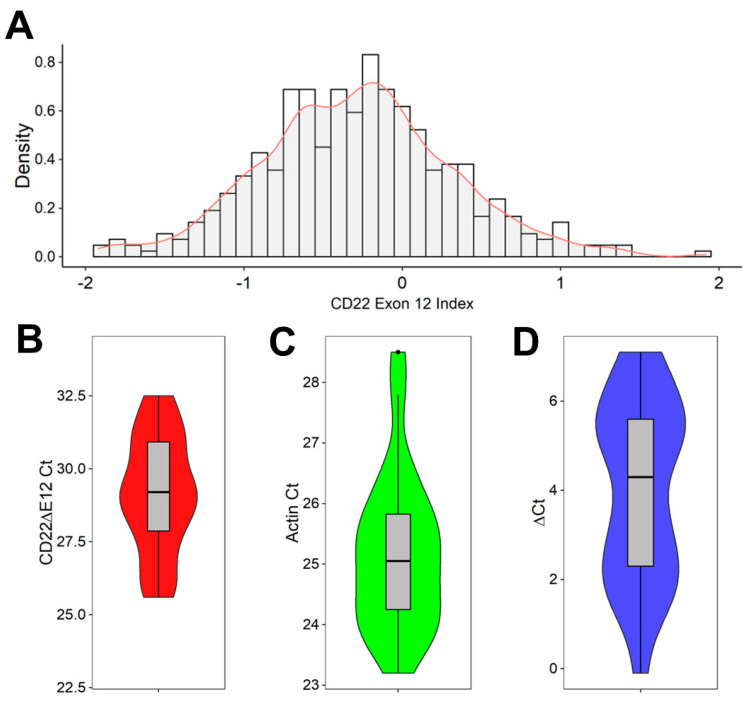
CD22ΔE12 in pediatric B-ALL. (**A**,**B**) Reduced expression levels of CD22E12 in primary leukemia cells from B-ALL patients. (**A**) Distribution of CD22E12 mRNA expression in primary leukemia cells from B-ALL patients (GSE13159, GSE11877, and GSE13351) using the density graph of the CD22E12 index values fitted by applying a Gaussian smoothing kernel density estimation for B-ALL samples (mean = −0.288 ± 0.03 (median = −0.287; interquartile range = −0.67–0.08; range = −1.921–1.917; *n* = 421) (ggplot2_3.3.5 R package). The CD22E12 index values were calculated by subtracting the mean expression values obtained with the six probes for CD22E11, CD22E13, and CD22E14 in the 217422_s_at probe set from the mean expression values obtained using the three CD22E12 probes. (**B**–**D**) Detection of CD22ΔE12 mRNA in primary leukemia cells from 24 pediatric B-ALL patients using quantitative RT-PCR. The PCR primer pair was designed to amplify a 113-bp fragment spanning from exon 11 to exon 13 of the human CD22 cDNA, as described in Materials and Methods. Depicted in (**B**,**C**) are the Ct values for CD22ΔE12 and β-actin (as the mRNA of a control house-keeping gene). Depicted in (**D**) are the ΔCt values (CD22ΔE12 expression normalized for the expression level of β-actin). The results in (**B**–**D**) are visualized as superimposed box plots and kernel density plots. The grey boxes depict, as horizontal lines within the box, the median values for CD22ΔE12 Ct (in (**C**)), β-Actin Ct (in (**C**)), and CD22ΔE12 ΔCt (in (**D**)); the boxes represent the 75th and 25th quantiles, and the whiskers represent the 3rd quartile + 1.5×(interquartile range) and 1st quartile—1.5×(interquartile range) of the values. The colored density plots (violin plots with mirrored density on the vertical axis) visualize the distribution of the individual Ct and ΔCt values, where increased broadness of the plot indicates an increase in the density of Ct and ΔCt values.

CD22ΔE12 is associated with a selective reduction in expression levels of CD22E12 mRNA [8,9,10,11,12,13,14]. We first examined the distribution of CD22E12 mRNA expression levels in primary leukemia cells from 421 B-ALL patients, as measured by the CD22E12 index values based on the transcriptome profiling data obtained using the genome expression microarray platform (Figure 1A). The CD22E12 index values for B-ALL samples showed interpatient variability (mean ± SE = −0.29 ± 0.03; median = −0.29; range = −1.92–1.92; *n* = 421) (Figure 1A). The density graph of the CD22E12 index values for B-ALL cells showed broad multipeak distributions consistent with marked patient-to-patient heterogeneity in CD22E12 expression levels. Next, we used qRT-PCR to examine the CD22ΔE12 expression levels in primary leukemia cells from 24 pediatric B-ALL patients (Figure 1B–D). The displayed ΔCt values demonstrated marked interpatient heterogeneity and broad multipeak distributions in CD22ΔE12 expression levels. The data on the microarray-based CD22E12 index values combined with the qRT-PCR-based data on CD22ΔE12 Ct and ΔCt values indicate that the biological impact of CD22ΔE12 may vary from patient to patient due to varying levels of normal CD22 and truncated CD22 levels.

### 3.2. Interpatient Heterogeneity in Selective Reduction of RNAseq-Based CD22E12 Expression Levels among B-ALL Patients

**Figure 2:**
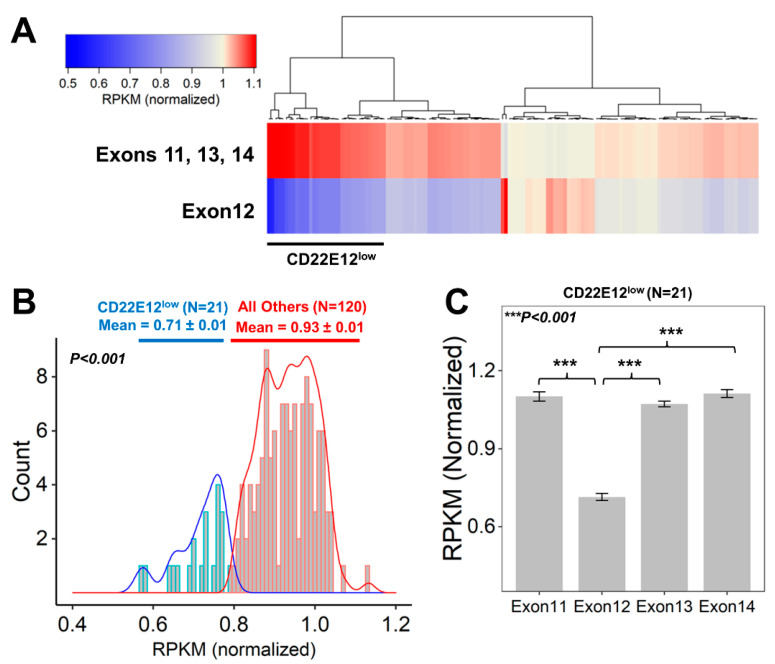
Identification of newly diagnosed B-ALL patients who exhibited CD22ΔE12-associated selective reductions of CD22E12 relative to the expression of CD22 exons 11 to 14. Data files reporting the “reads per kilobase million (RPKM)” metric for CD22 gene exon-level quantification of mRNA for CD22 exons 11–14 were downloaded from the TARGET phase 2 project (https://target-data.nci.nih.gov/Public/ALL/mRNA-seq/Phase2/L3/, accessed on 9 July 2022). The expression level of each exon was mean-centered to the average RPKM values across exons 11 to 14 for each of the 141 B-ALL patients to obtain the “normalized” RPKM values. A subset of 21 patients with CD22ΔE12-associated selective reductions of CD22E12 (CD22E12^low^) were identified via examination of the RPKM values for CD22E12 and the adjacent CD22 exons 11 (CD22E11), 13 (CD22E13), and 14 (CD22E14). (**A**) The cluster figure depicts the average normalized RPKM values for CD22 exons 11, 13, and 14 (top row) and 12 (bottom row) for each patient (across columns). Heat map: Blue represents underexpression, and red represents overexpression. The clustering algorithm (Euclidean distance and Wards linkage) identified 21 patients with low levels of CD22 exon 12 expression relative to exons 11, 13, and 14 (black bar for CD22E12^low^ patients). (**B**) Depicted are histograms of the distribution profile of normalized RPKM values for CD22E12 in (i) CD22E12^low^ patients (*n* = 21) as blue lines and in (ii) other patients (*n* = 120) as red lines. The density plots under the histograms shown as blue (CD22E12^low^ patients) or red (all other patients) bars represent the normalized RPKM values. For CD22E12^low^ patients, the normalized RPKM values were <0.8. The mean value for CD22E12 expression level (in normalized RPKM) for CD22E12^low^ patients was 0.714 ± 0.014 (median = 0.728; range = 0.572–0.785), which was significantly lower than the mean CD22E12 expression level of 0.934 ± 0.006 (median = 0.935; range = 0.805–1.133) for the remaining 120 patients (two-way ANOVA, linear contrast, *p*-value < 10^−15^). (**C**) The average (mean ± SEM, normalized RPKM) expression levels for CD22E11, CD22E12, CD22E13, and CD22E14 depicted in bar charts for the CD22E12^low^ patient subset (*n* = 21) illustrate the selective reduction of CD22E12 (Exon12) relative to exons 11, 13, and 14. CD22E12 expression levels for CD22E12^low^ patients (mean = 0.714 ± 0.014; median = 0.728; range = 0.572–0.785) were significantly lower than the expression levels for CD22E11 (mean = 1.101 ± 0.018; median = 1.109; range = 0.839–1.224; *p*-value < 10^−15^), CD22E13 (mean = 1.073 ± 0.011; median = 1.076; range = 0.961–1.151; *p*-value < 10^−15^), and CD22E14 (mean = 1.112 ± 0.015; median = 1.103; range = 1.026–1.279; *p*-value < 10^−15^).

We next examined the relative expression levels of CD22E12 in primary leukemia cells from 141 patients with B-ALL by interrogating the archived exon-level quantitative RNAseq data from the TARGET program. In accordance with the results obtained with the microarray platform and qRT-PCR data, the RNAseq data showed marked heterogeneity in the magnitude of the selective reduction of CD22E12 mRNA expression levels, as evidenced by the heat map of the cluster figure (Figure 2A) and the broad distribution of the normalized RPKM values for CD22E12 (Figure 2B). By using the method of hierarchical clustering, a subset of 21 patients (“CD22E12^low^”) with selective reduction CD22E12 levels relative to CD22E11, CD22E13, and CD22E14 levels was identified, in which the normalized RPKM value for CD22E12 mRNA was <0.8 (Figure 2B). The mean CD22E12 expression level (in normalized RPKM) for the CD22E12^low^ patients was 0.714 ± 0.014 (median = 0.728; range = 0.572–0.785), which was significantly lower than the mean CD22E12 expression level of 0.934 ± 0.006 (median = 0.935; range = 0.805–1.133) for the remaining 120 patients (two-way ANOVA, linear contrast, *p*-value < 10^−15^) (Figure 2B). The CD22E12 expression level for this subset was significantly lower than the expression levels for exons CD22E11 (mean = 1.101 ± 0.018; median = 1.109; range = 0.839–1.224; *p*-value < 10^−15^), CD22E13 (mean = 1.073 ± 0.011; median = 1.076; range = 0.961–1.151; *p*-value < 10^−15^), and CD22E14 (mean = 1.112 ± 0.015; median = 1.103; range = 1.026–1.279; *p*-value < 10^−15^). (Figure 2C).

In addition to CD22ΔE12, CD22 exon 2 (CD22E2) skipping that results in very low CD22E2 mRNA levels also occurs in B-ALL and is associated with resistance to CD22-directed immunotherapies due to reduced expression of the target CD22 protein. As both events are caused by aberrant splicing, we next evaluated CD22E2 mRNA levels in CD22E12^low^ patients in an effort to determine if CD22E12^low^ and CD22E2^low^ subsets overlap in the studied B-ALL patient population (*n* = 141). A subset of 34 patients (24.1%) with CD22E2 reductions (CD22E2^low^) were identified via examination of the RPKM values for CD22E2 and the adjacent CD22 exons 1 (CD22E1), 3 (CD22E3), and 4 (CD22E4) (Figure S1). The normalized RPKM value for each patient in this CD22E2^low^ subset was less than 0.397. The mean CD22E2 expression level for the CD22E2^low^ patients was 0.295 ± 0.016, which was significantly lower than the mean CD22E2 expression level of 0.565 ± 0.014 for the remaining 107 patients (Welch two-sample *t*-test, T = 12.673, df = 83.3, *p*-value < 10^−15^) (Figure S1). Among the 21 CD22E12^low^ patients and 120 others, 8 (38%) and 26 (22%) were CD22E2^low^ (Fisher’s exact test, *p* = 0.16). The density plots for CD22E2 mRNA expression for CD22E12^low^ vs. other patients displayed a near-complete overlap (Figure S2). The mean CD22E2 expression level for the CD22E12^low^ patients was 0.46 ± 0.039, which was not significantly different from the mean CD22E2 expression level of 0.507 ± 0.016 for the remaining 120 patients (Welch two-sample *t*-test, T = 1.13, df = 27.2, *p*-value = 0.27). The CD22E2 expression levels among the 34 CD22E2^low^ patients were very similar for the 8 CD22E12^low^ subset vs. the remaining 26 patients (0.298 ± 0.032 vs. 0.295 ± 0.019). Likewise, the CD22E2 expression levels for the 107 non-CD22E2^low^ patients did not show significant differences between 13 CD22E12^low^ patients and the remaining 94 patients (0.56 ± 0.039 vs. 0.566 ± 0.015) (two-way ANOVA, FDR-adjusted *p*-value = 0.96 for both CD22E2^low^ and all other patients comparisons) (Figure S2). These results demonstrate that CD22E12^low^ status applies to a smaller fraction of B-ALL patients (14.9% vs. 24.1%, *p* = 0.16, taking into account eight patients (6%) who were both CD22E12^low^ and CD22E2^low^) and does not show an apparent relationship to the more frequent CD22E2^low^ status.

### 3.3. Presenting Features of CD22E12^low^ B-ALL Patients

We next sought to determine if the CD22E12^low^ patient subset is characterized by an enrichment of high-risk prognostic markers within the confines of a relatively small sample size (*n* = 141). As shown in Table 1, the patient characteristics exhibited similar ages and gender/race/ethnicity distribution, and they did not show marked differences between CD22E12^low^ patients and the remainder of the patient population (Table S1 and Table 1). The mean age was 8.2 ± 1.2 years (median = 7.6 years) for CD22E12^low^ patients vs. 7.9 ± 0.5 years (median = 6.4 years) for other patients (*p* = 0.8). A total of 11 of 21 (52.4%) CD22E12^low^ patients vs. 53 of 120 (44.2%) other patients were in the high-risk age category (age < 2 years or ≥10 years) (*p* = 0.6, Table 1). There was no enrichment for adult patients (viz.: >18 years of age; 1/21 (4.8%) CD22E12^low^ patients versus 4/120 (3.3%) others; *p* = 0.6). The mean WBC was 45.5 ± 13.1 × 10^9^/L (median = 15.9 × 10^9^/L) for CD22E12^low^ patients and 76.8 ± 12.0 10^9^/L (median = 33 × 10^9^/L) for other patients (*p* = 0.2). A total of 11 of 21 (52.4%) CD22E12^low^ patients vs. 79 of the remaining 120 patients (65.8%) had NCI high-risk ALL (Fisher’s Exact, 2-tailed test, *p* = 0.3, Table 1). There was no enrichment for patients with higher WBC (viz.: WBC ≥ 20 × 10^9^/L (10/21 (47.6%) vs. 73/120 (60.8%), *p* = 0.3), CNS2 or CNS3 category (5 of 21 CD22E12^low^ patients (23.8%) versus 23 of 120 (19.2%) other patients, *p* = 0.6), poor-risk cytogenetics assessed by karyotyping (pseudodiploidy, hyperdiploidy, or hypodiploidy with structural chromosomal abnormalities (SCA) (i.e., 7 of 12 (58.3%) evaluable CD22E12^low^ patients versus 71 of 94 (75.5%) evaluable other patients, *p* = 0.3). None of the evaluable CD22E12^low^ patients were t(9;22)/BCR-ABL1^+^ or MLL-R/t(4;11)^+^.

Three out of twenty (15%) evaluable CD22E12^low^ patients were TCF3-PBX1^+^ compared to 11/112 (9.8%) evaluable other patients (*p* = 0.4). We noticed that none of the 20 evaluable CD22E12^low^ patients had the favorable prognosis markers ETV6-RUNX1 or hyperdiploidy with trisomy 4 and 8. By comparison, among the remaining 112 evaluable other patients, 11 (9.8%) were ETV6-RUNX1^+^ (*p* = 0.2), and 10 (8.9%) had trisomy 4 and 10 (0/21 (0%) vs. 21/112 (18.8%), *p* = 0.042). Notably, there was no apparent evidence for intrinsic resistance to induction chemotherapy. There were only two induction failures in this group of patients; none of the 21 CD22E12^low^ patients and 2 of the 119 other patients in the RNA-seq subset experienced an induction failure. At the end of the induction therapy, 57.1% of CD22E12^low^ patients and 46.7% of the remaining patients had no measurable residual disease (MRD), as measured with 6-color flow cytometry (*p* = 0.5). The mean day 29 MRD values were 0.07 ± 0.03% for CD22E12^low^ patients and 0.87 ± 0.35% for the remaining patients (*p* = 0.4). End-of-consolidation MRD data was available for only three CD22E12^low^ patients, and they all were zero (Table 1). Within the NCI high-risk subset of 90 patients, only 3 of 52 patients (6%) with MRD > 0 and 8 of 38 patients (21%) with MRD = 0 were CD22E12^low^ (Fisher’s exact *p*-value = 0.048).

### 3.4. Gene Set Enrichment Analysis of Reactome Pathways in CD22E12^low^ B-ALL Patients

**Figure 3:**
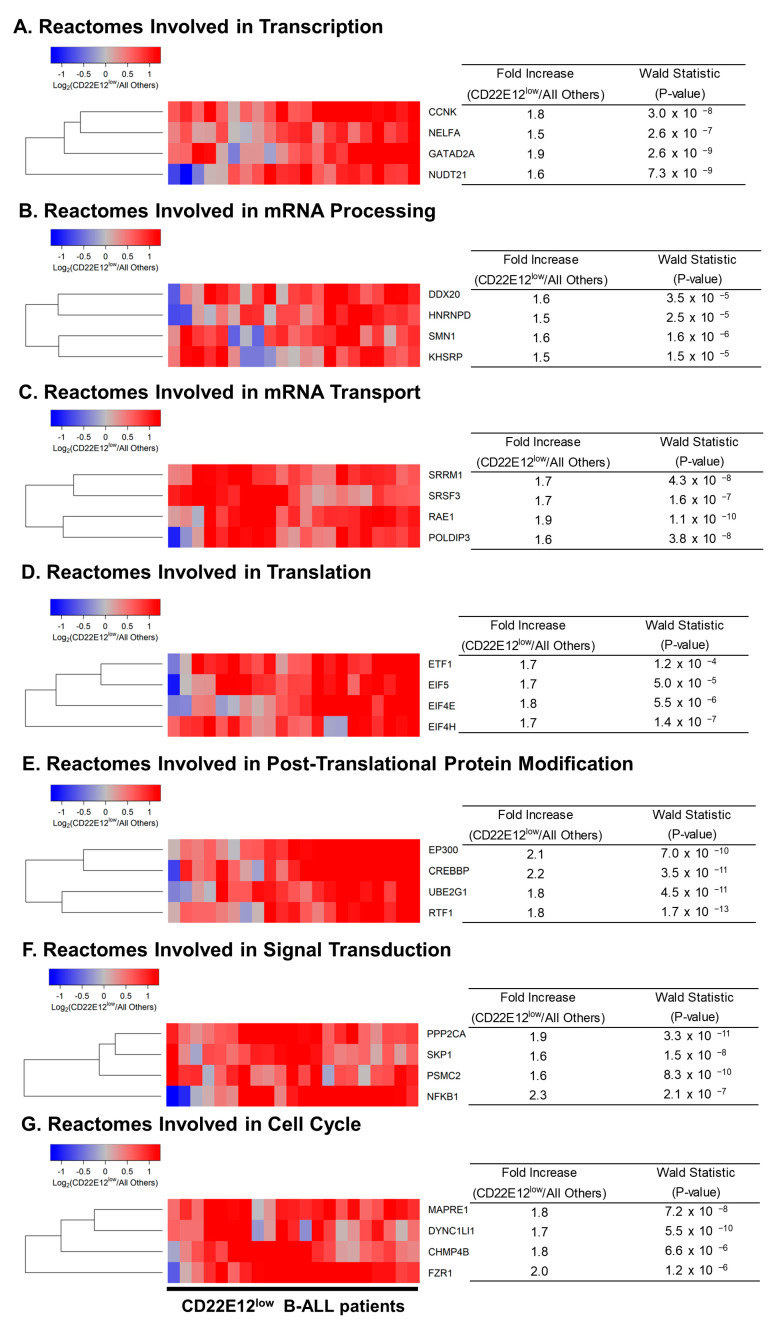
Most significantly overexpressed genes in CD22E12^low^ patients for reactomes representing transcription, translation, signal transduction, and cell cycle. We determined the differential expression of genes comparing CD22E12^low^ patients (*n* = 21) with all other patients (*n* = 120) using DESeq2 package (DESeq2_1.34.0 implemented in R). The cluster figures display the variance-stabilized, normalized log_2_ expression values in CD22E12^low^ B-ALL patients mean-centered to the expression levels in all other patients (blue represents underexpression and red represents overexpression in CD22E12^low^ patients). See Supplemental Figures S3–S7 and Tables S2 –S8 for more details regarding dysregulated gene sets, including up and downregulated genes. Depicted in (**A**–**G**) are, for each group of the seven reactome gene sets, the four genes with the highest values for Wald Statistics comparing CD22E12^low^ versus all other patients. Fold Increase values were calculated from the baseline mean of counts determined using the DESeq2 algorithm following removal of low-expression genes (mean ≤ 10 counts); median count for the depicted 28 genes was 14,492 (range = 546 − 60391; interquartile range = 5322 −24229 counts).

We used GSEA to examine the transcriptomes of primary leukemia cells from CD22E12^low^ B-ALL patients for selective disruptions of reactome pathways that are selectively affected in murine B-ALL cells from CD22ΔE12-Tg mice [9]. Notably, the expression of several genes represented in reactomes involved in transcription (Figure S3, Table S2), mRNA processing/mRNA transport (Figure S4, Tables S3 and S4), translation (Figure S5A, Table S5), post-translational protein modification (Figure S5B, Table S6), signal transduction (Figure S6, Table S7), and cell cycle (Figure S7, Table S8) were dysregulated in primary leukemia cells from CD22E12^low^ patients. Table 2 compares the affected reactome pathways in CD22E12^low^ human B-ALL cells vs. murine B-ALL cells originating from CD22ΔE12-Tg mice [9]. The most significantly upregulated genes in primary leukemia cells from CD22E12^low^ B-ALL patients, according to the affected reactome pathways they are represented in, exhibited fold-increase values over the expression values in primary leukemia cells from other patients ranging from 1.5 to 2.3 and included (i) CCNK, NELFA, GATAD2A, and NUDT21 (transcription); (ii) DDX20, HNRNPD, SMN1, and KHSRP (mRNA processing); (iii) SRRM1, SRSF3, RAE1, and POLDIP3 (mRNA transport); (iv) ETF1, EIF5, EIF4E, and EIF4H (translation); (v) EP300, CREBBP, UBE2G1, and RTF1 (post-translational protein modification); (vi), PPP2CA, SKP1, PSMC2, and NFKB1 (signal transduction); (vii) MAPRE1, DYNC1LJ1, CHMP4B, and FZR1 (cell cycle) (Figure 3).

### 3.5. Clinical Prognostic Significance of the Interpatient Heterogeneity in Selective Reduction of RNAseq-Based CD22 Exon 12 Expression Levels among B-ALL Patients

**Figure 4:**
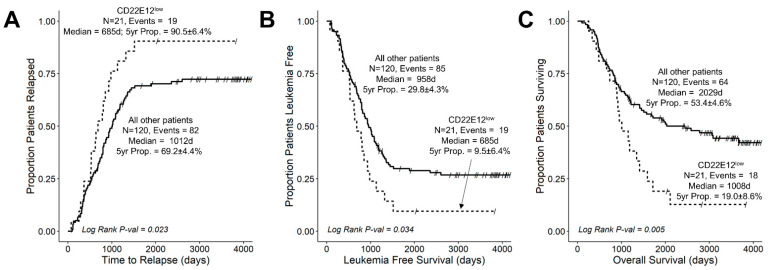
Unfavorable impact of CD22DE12-associated selective reduction of CD22E12 expression on treatment outcomes in newly diagnosed B-ALL. Treatment outcomes were compared using Kaplan–Meier analysis for the CD22E12^low^ subset (*n* = 21) vs. other patients (*n* = 120). See Table 1 for patient characteristics. (**A**) Relapse. (**B**) LFS. (**C**) OS. CD22E12^low^ patients exhibited a higher probability of relapse, shorter time to relapse, worse LFS with shorter times to first event (relapse or death), and worse OS. See text for discussion of results.

We next evaluated the potential impact of reduced CD22 exon 12 expression on the treatment outcomes in B-ALL by comparing the outcomes of CD22E12^low^ patients to the outcomes of the remaining patients. The median follow-up time was 963 days (range = 77–4175 days; interquartile range = 549–1948 days) for the 141 B-ALL patients with RNA-seq data. Among these patients, the median follow-up times were 867 days (range = 77–3830 days; interquartile range = 528–1220 days) for the 21 CD22E12^low^ patients and 1017 days (range = 98–4175 days; interquartile range = 558–2426 days) for the remaining 120 patients. A total of 101 of 141 patients experienced a relapse after attaining remission. CD22E12^low^ patients had a significantly higher incidence of relapse (19/21; 90.4%) than other patients (82/120 (68%) (Fisher’s exact test, *p* = 0.039). The probability of relapse within five years was 90.5 ± 6.4% for CD22E12^low^ subset and 69.2 ± 4.4% for other patients (*p* = 0.023) (Figure 4A). Further, the 685-day median time to relapse (95% CI: 531–1129) in CD22E12^low^ patients was significantly shorter than the median time to relapse in the remaining patients (median: 1012, 95% CI: 819–1191 days; log-rank chi-square = 5.19, *p*-value = 0.023) (Figure 4A). CD22E12^low^ patients had significantly worse LFS outcomes than other patients (Figure 4B). The probability of surviving leukemia-free at 5-years was 9.5 ± 6.4% for the CD22E12^low^ subset and 29.8 ± 4.3% for other patients (*p* = 0.039). The median LFS time for CD22E12^low^ patients was 685 (95% CI: 531–1129) days, which was significantly shorter than the median LFS time of 958 (95% CI: 801–1178) days for other patients (log rank chi-square = 4.48, *p*-value = 0.034) (Figure 4B). The OS outcome of CD22E12^low^ patients was significantly worse than the OS outcome of other patients (Figure 4C). The probability of being alive after five years was 19.0 ± 8.6% for the CD22E12^low^ subset and 53.9 ± 4.6% for other patients (*p* = 0.004). The median OS time for CD22E12^low^ patients was 1008 days (need 95% CI = 883–1715), which is significantly shorter than the median OS time of 2029 days (95% CI = 1456-NA) for the remaining patients (log-rank chi-square = 7.82, *p*-value = 0.005) (Figure 4C).

We next asked if CD22E12^low^ status remained a poor prognostic indicator for LFS and OS in the 90-patient subset of NCI high-risk B-ALL patients. CD22E12^low^ patients (*n* = 11) within the high-risk subset of patients had worse treatment outcomes than the remaining patients (*n* = 79) (Figure S8). CD22E12^low^ high-risk B-ALL patients had a shorter time to relapse (median values: 625 days (95% CI = 520–NA) vs. 1083 days (95% CI = 881–NA); log-rank chi-square = 4.19, *p*-value = 0.04) (Figure S8A), shorter LFS (median values: 625 days (95% CI = 520–NA) vs. 1040 days (95% CI = 863–NA); log rank chi square = 3.7, *p*-value = 0.05) (Figure S8B), and shorter OS (median values: 1008 days (95% CI = 736–NA) vs. 2029 days (95% CI = 1413–NA); log-rank chi-square = 4.58, *p*-value = 0.03) than other high-risk B-ALL patients (Figure S8C). The probability of high-risk B-ALL patients to be alive and leukemia-free after five years was 18.2 ± 11.6% for the CD22E12^low^ subset and 46.4 ± 5.6% for other patients (*p* = 0.1). The probability of high-risk patients to be alive after five years was 18.2 ± 11.6% for the CD22E12^low^ subset and 54.3 ± 5.7% for other patients (*p* = 0.025).

**Figure 5:**
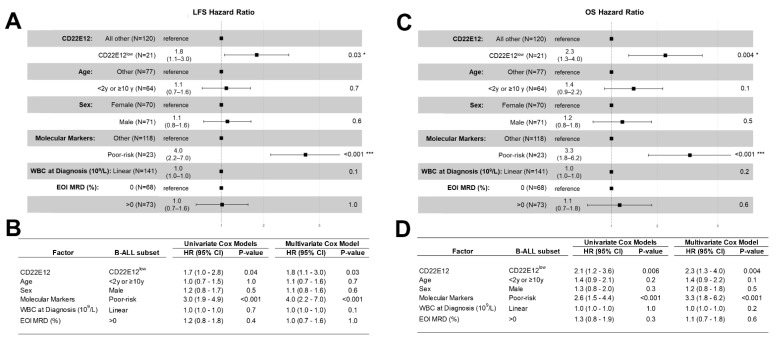
CD22E12^low^ status in B-ALL as a poor prognostic indicator in univariate and multivariate Cox proportional hazards models comparing LFS and OS outcomes. We examined the effects of CD22E12^low^ status as an indicator of poor prognostic prognosis that is associated with worse LFS and OS outcomes in univariate and multivariate Cox proportional hazards models. These models include as variables the following candidate poor prognostic characteristics for hazard ratio (HR) determinations: (i) CD22E12^low^ status; (ii) age < 2 y or ≥10 y; (iii) male; (iv) poor-risk classification based on molecular markers (BCR-ABL1^+^, MLL-R^+^, or TCF3-PBX1^+^); (v) WBC x 10^9^/L at diagnosis as a linear covariate; and (vi) end-of-induction day 29 MRD burden > 0 (i.e., MRD ≥ 0.001%). (**A**) Depicted are the Forest plots for LFS along with the corresponding HRs and *p*-values for each covariate in the multivariate Cox proportional hazards model. * *p* < 0.05; *** *p* < 0.001. (**B**) The HR for LFS and corresponding *p*-values are provided for each variable in both the univariate and multivariate models. CD22E12^low^ status (HR = 1.7 ± 0.3, *p*-value = 0.036) and having a poor prognostic molecular marker (HR = 3.0 ± 0.2, *p*-value = 8 × 10^−6^) were significant predictors of poor LFS in the univariate model. Both CD22E12^low^ status (HR = 1.8 ± 0.3, *p*-value = 0.03) and having a poor prognostic molecular marker (HR = 4 ± 0.3, *p*-value = 2 × 10^−6^) also exhibited statistically significant increases in HR for LFS in the multivariate model. (**C**) Depicted are the Forest plots for OS along with the corresponding HRs and *p*-values for each covariate in the multivariate Cox proportional hazards model. * *p* < 0.05; *** *p* < 0.001 (**D**) The HR for OS and corresponding *p*-values are provided for each variable in both the univariate and multivariate models. CD22E12^low^ status (HR = 2.1 ± 0.3, *p*-value = 0.006) as well as having a poor prognostic molecular marker (HR = 2.6 ± 0.3, *p*-value = 4.2 × 10^−4^) were identified as predictors of poor OS in the univariate model. Both variables were also identified as significant and independent predictors of poor OS outcome in the multivariate model (HR for CD22E12^low^ status = 2.3 ± 0.3, *p*-value = 0.004; HR for having a poor prognostic molecular marker = 3.3 ± 0.3, *p*-value = 2.2 × 10^−4^).

We also examined the effects of CD22E12^low^ status as an indicator of poor prognosis associated with a poor LFS as well as poor OS in univariate and multivariate Cox proportional hazards models. These models include as variables the following candidate poor prognostic characteristics for hazard ratio (HR) determinations: (i) CD22E12^low^ status; (ii) age < 2 y or ≥ 10 y; (iii) male; (iv) poor risk classification based on molecular markers (BCR-ABL1^+^, MLL-R^+^, or TCF3-PBX1^+^); (v) WBC at diagnosis (as a linear covariate); and (vi) end-of-induction day 29 MRD burden > 0 (defined as MRD ≥ 0.001%). CD22E12^low^ status was associated with an increased hazard ratio for shorter LFS in both univariate (HR = 1.7 ± 0.3, 95% CI: 1.0–2.8, *p*-value = 0.04) and multivariate models (HR = 1.8 ± 0.3, 95% CI: 1.1–3.0, *p*-value = 0.03) (Figure 5A,B). Similarly, CD22E12^low^ status was associated with an increased hazard ratio for shorter OS in both univariate (HR = 2.1 ± 0.3, 95% CI: 1.2–3.6, *p*-value = 0.006) and multivariate models (HR = 2.3 ± 0.3, 95% CI: 1.3–4.0, *p*-value = 0.004) (Figure 5C,D). In the multivariate Cox model, the HR values for CD22E12^low^ status (viz.: 1.8 for LFS and 2.3 for OS) were not as high as those associated with a poor-risk molecular marker profile (viz.: 4.0 for LFS and 3.3 for OS) but higher than those associated with any other poor risk variable analyzed.

Notably, the comparison of the LFS and OS outcomes for the subset of 90 evaluable high-risk B-ALL patients also showed a significant increase in HR for patients with CD22E12^low^ status (*n* = 11) compared to other patients (*n* = 79) (Figure S9). In the multivariate Cox regression model, CD22E12^low^ status was found to be a significant and independent predictor of poor LFS outcomes (HR = 3.1 ± 0.4, *p*-value = 0.01) as well as poor OS outcomes (4.0 ± 0.5, *p*-value = 0.003). The HR values for CD22E12^low^ status (viz.: 3.1 for LFS and 4.0 for OS) were higher than those associated with any other poor risk variable included in the multivariate Cox model.

## 4. Discussion

We had originally hypothesized that CD22ΔΕ12 might act as an oncogenic protein by competitively binding to the cis ligands of CD22 and preventing residual wildtype CD22 from in cis ligand binding, thereby contributing to the increased proliferation and defective apoptosis of leukemic B-cell precursors from B-ALL patients caused by the CD22ΔE12-associated impaired regulatory function of CD22 [7,8]. Expression of a truncated CD22ΔE12 protein was indeed associated with aggressive in vivo growth of primary B-ALL cells in immunodeficient NOD/SCID mice [7]. Lentiviral-based overexpression of truncated CD22ΔE12 protein but not full-length CD22 in B-ALL cells resulted in a marked increase in the sizes of blast colonies in vitro, consistent with increased self-renewal and clonogenicity [13]. Further, CD22ΔE12 depletion via CD22ΔE12-specific small interfering RNA (siRNA) and their liposomal formulations inhibited the in vitro and in vivo clonogenicity of B-ALL cells [8,9,13]. These experimental findings collectively support our hypothesis regarding the oncogenic function of CD22ΔE12 protein and demonstrate that CD22ΔE12 expression is associated with the selective survival and growth advantage of B-ALL cells [7,8,9,10,11,12,13,14].

Our initial studies demonstrated that CD22ΔE12, with the selective reduction of CD22E12 levels, is a characteristic splicing defect in primary leukemia cells from newly diagnosed high-risk and relapsed B-ALL patients [10]. We now report for the first time that in newly diagnosed B-ALL, CD22E12^low^ patients with very low CD22E12 levels, who represent 14.9% of the patient population, had significantly worse LFS and OS outcomes than other patients. Our study thereby fills a significant gap in our understanding of the clinical significance of CD22ΔE12 in B-ALL. The cumulative proportion of patients in the analyzed population of 141 newly diagnosed B-ALL patients who survived after five years was 19.0 ± 8.6% for the CD22E12^low^ subset (*n* = 21) and 53.4 ± 4.6% for the remaining 120 patients (*p* = 0.004). The median OS time for CD22E12^low^ patients was 1008 days, which is significantly shorter than the median OS time of 2029 days for the remaining patients (log-rank chi-square = 7.8, *p*-value = 0.005). Likewise, in the 90-patient subset of NCI high-risk B-ALL patients, CD22E12^low^ patients (*n* = 11) had significantly worse treatment outcomes than the remaining 79 patients (*n* = 79).

Importantly, CD22E12^low^ status was associated with a significantly increased hazard ratio for shorter PFS and OS in both univariate and multivariate models. In the multivariate Cox regression model for the NCI high-risk subset that also included patient age, sex, WBC at presentation, end-of-induction MRD burden, and poor-risk cytogenetics/FISH markers (BCR-ABL1^+^, MLL-R^+^ or TCF3-PBX1^+^) as covariables, we found CD22E12^low^ status to be a significant and independent predictor of poor LFS outcomes (HR = 3.1 ± 0.4, *p*-value = 0.01) as well as poor OS outcomes (HR = 4.0 ± 0.5, *p*-value = 0.003). These new results significantly extend a previous study that implicated CD22ΔE12 as a driver lesion, contributing to the aggressive biology of relapsed pediatric B-ALL [10]. CD2E12^low^ status at presentation may have clinical utility as a poor prognostic biomarker and may help guide the early allocation of risk-adjusted, patient-tailored treatment regimens as well as refine risk classification in high-risk B-ALL.

Forced overexpression of the mutant CD22ΔE12 in transgenic mice caused fatal B-ALL, demonstrating that CD22ΔE12 alone may be sufficient as a driver lesion for the leukemic transformation and aggressive in vivo growth of B-cell precursors [7,8]. Notably, genes related to transcriptional, translational, signal transduction-related, and cell cycle-related reactome pathways were significantly upregulated in B-ALL cells from CD22ΔE12 Tg mice [9]. We previously reported that the CD22ΔE12 signature transcriptome encodes a phosphoproteome that is differentially overexpressed in CD22ΔE12-Tg B-ALL cells, confirming that CD22ΔE12 corrupts the regulation of multiple signaling networks. Several anti-apoptotic proteins such as mTOR, AKT, NFκB, transcription factors implicated in oncogenesis, and serine kinase signaling pathway proteins such as MAPK, PKC, and PKD were among the most significantly overexpressed members of the CD22ΔE12 signature phosphoproteome [9,13]. In the current study, we used GSEA in gene sets obtained from the Bioconductor Reactome Database to examine the transcriptomes of primary leukemia cells from CD22E12^low^ B-ALL patients for selective disruptions of reactome pathways we had previously discovered to be selectively dysregulated in murine B-ALL cells from CD22ΔE12-Tg mice [9,13]. Notably, the expression of several genes represented in reactomes involved in transcription, mRNA processing/mRNA transport, translation, post-translational protein modification, signal transduction, and cell cycle were selectively amplified in primary leukemia cells from CD22E12^low^ patients. These findings demonstrate that the regulation of gene expression is corrupted in CD22E12^low^ B-ALL cells across multiple reactomes, which is reminiscent of the CD22ΔE12-associated transcriptome changes in transgenic mice [9,13]. We propose that the observed differences in gene expression profiles of CD22E12^low^ vs. other B-ALL patients may contribute to the seemingly more aggressive biology of the CD22E12^low^ subset.

RNAi therapeutics targeting CD22ΔE12 may disrupt the signaling networks that promote the proliferation and survival of CD22ΔE12^+^ B-ALL cells [8,9,13]. We previously reported preclinical data regarding the in vitro and in vivo anti-leukemic activity of nanoformulations of CD22ΔE12-specific siRNA as an innovative new treatment platform for CD22ΔE12^+^ B-ALL [8,9,13]. Further development and optimization of this experimental platform may have clinical potential. The clinical development of personalized nanomedicines against the CD22ΔE12 poor-risk B-ALL might address an unmet challenge in treating B-ALL by improving the landscape of effective treatment modalities. The observed poor prognosis of newly diagnosed B-ALL patients with CD22ΔE12-associated CD22E12^low^ status, as reported here, supports the notion that further exploration of the clinical potential of CD22ΔE12-targeting RNAi therapeutics is warranted.

Black et al. reported mutations in splicing factor genes, including the genes for hnRNPs, as a possible mechanism for aberrant splicing in B-ALL [27]. Pathogenic intronic mutations were implicated in aberrant splicing and human disease [28,29]. In patients with CD22ΔE12, we previously reported multiple homozygous mutations within a 132-bp mutational hotspot segment of the intronic sequence between exons 12 and 13, some of which involved locations of known single nucleotide polymorphisms (SNP) [7]. Within this intronic segment, we identified multiple accessible potential binding sites for the heterogenous nuclear ribonucleoprotein (hnRNP) family of splicing factors that act as global regulators of alternative splicing [7,30,31]. The documented mutations within the hot spot region were associated with secondary structure conformation and folding patterns that affected the target motifs for hnRNP-E2/PCBP, hnRNP-I/PTB, and hnRNP-L as well as the surrounding structure of the predicted pre-mRNA [7]. Therefore, we proposed that these mutations might contribute to aberrant pre-mRNA splicing by affecting the recognition of the 5′ splice site of CD22E12 via the splicing factors and preventing an orderly assembly of the splicesome assembly [7]. In addition to CD22ΔE12, CD22E2 skipping by aberrant splicing [32,33] also occurs in B-ALL, which results in very low CD22E2 mRNA levels and is associated with inherent resistance to CD22-directed immunotherapies such as inotuzumab, ozogamicin, and CAR-T cells [34,35,36] due to reduced expression of the target CD22 protein. As both events are caused by aberrant splicing, we evaluated CD22E2 mRNA levels in CD22E12^low^ patients in an effort to determine if CD22E12^low^ and CD22E2^low^ subsets overlap in the studied B-ALL patient population. Our findings indicate that the CD22E12^low^ status applies to a smaller fraction of B-ALL patients and does not show an apparent relationship to the more common CD22E2^low^ status. Therefore, CD22-targeting immunotherapeutics remain a viable treatment modality for a significant portion of CD22E12^low^ patients without evidence of CD22E2 skipping and/or a marked reduction of CD22 expression levels. The present study has a significant limitation owing to a patient selection bias caused by the availability of RNA-seq data for only 12.5% of patients in the TARGET database who had worse leukemia-free survival and overall survival outcomes than the total patient population in the database. A number of statistically significant differences in patient characteristics suggests that the 141-patient RNA-seq subset was likely enriched for cases with a biologically more aggressive disease, including a higher mean WBC at diagnosis in the RNA-seq subset (72.2 ± 10.4 63.7 ± 3.2, *p* = 0.046), a higher portion of patients with CNS 2 or CNS 3 at diagnosis in the RNA-seq subset (19.9% vs. 11.6%, *p* = 0.003), a higher incidence of CNS relapse in the RNA-seq subset (10.6% vs. 5.1%, *p* = 0.003, odds ratio: 2.6), a higher portion of patients with MRD > 0 on day 29 in the RNA-seq subset (51.8 vs. 41.8%, *p* = 0.014), and a higher portion of patients with structural chromosomal abnormalities in the RNA-seq subset (73.6% vs. 64.4%, *p* = 0.04, odds ratio: 1.6) (Table S9). Although these differences might have contributed to poor survival outcomes, it was not possible to decipher the exact reasons of the worse-than-expected survival outcomes in the analysis population, as patient-specific treatment information was not archived in the TARGET database. Another limitation of the study relates to the fact that some of the prognostically relevant data, such as the MRD values at the end of consolidation, were not collected for all patients. Although the characteristics of the CD22E12^low^ patients within the RNA-seq subset were very similar to those of the remainder of patients in the RNA-seq subset, which allowed an accurate comparison of the outcomes of the CD22E12^low^ and CD22E12^high^ patients (Table 1, Table S1), a hypothesis-testing prospective validation study will be necessary to confirm the poor prognostic effect of CD22E12^low^ status, preferably with RT-qPCR and RNA-seq in a larger B-ALL patient population treated according to a contemporary standard of care regimen.

## 5. Conclusions

Our study demonstrates that newly diagnosed B-ALL patients with very low levels of residual wildtype CD22 (“CD22E12^low^”), as measured by RNAseq-based CD22E12 mRNA levels, have significantly worse LFS as well as OS than other B-ALL patients. CD22E12^low^ status was identified as a poor prognostic indicator in both univariate and multivariate Cox proportional hazards models. CD22E12^low^ status at presentation shows clinical potential as a poor prognostic biomarker that may guide the early allocation of risk-adjusted, patient-tailored treatment regimens and refine risk classification in high-risk B-ALL.

## Figures and Tables

**Table 1:** Patient Characteristics According to CD22E12^low^ Status.

Variable	CD22E12^low^ (N = 21)		All Others (N = 120)		*p*-Value	
	Mean/Median(Range) orN (% Evaluable)		Mean/Median(Range) or N (% Evaluable)		Mann-Whitney U Test or Fisher’s Exact	
**Age (yrs)**						
Mean ± SEM/Median (Range)	8.2 ± 1.2	7.6 (1.4–18.1)	7.9 ± 0.5	6.4 (1.2–30)	0.8	
**WBC (×10^9^/L)**						
Mean ± SEM/Median (Range)	45.5 ± 13.1	15.9 (1.3–214.5)	76.8 ± 12	33 (1.1–1149)	0.2	
**MRD at Day 29**						
Mean ± SEM/Median (Range)	0.07 ± 0.03	0 (0–0.57)	0.9 ± 0.4	0 (0–26)	0.4	
**MRD at End of Consolidation**						
Mean ± SEM/Median (Range)	0 ± 0 (N = 3)	0 (0–0)	1.6 ± 1.4 (N = 23)	0 (0–31.5)	0.3	
**Age category**						
Adult (≥18 yrs)	1/21	(4.8%)	4/120	(3.3%)	0.6	
**CNS Status at Diagnosis**						
CNS 2 + CNS 3 **Induction Failure**	5/21 0/21	(23.8%) (0%)	23/120 2/119	(19.2%) (1.7%)	0.6 1.0	
**NCI Risk**						
High Risk	11/21	(52.4%)	79/120	(65.8%)	0.3	
**Age risk**						
Poor (Age <2 yrs or ≥10 years)	11/21	(52.4%)	53/120	(44.2%)	0.6	
**WBC category**						
≥20 × 10^9^/L **MRD at Day 8** MRD > 0	10/21 7/7	(47.6%) (100%)	73/120 33/33	(60.8%) (100%)	0.3 1.0	
**MRD at Day 29**						
MRD > 0 **MRD at End of Consolidation** MRD > 0	9/21 0/3	(42.9%) (0%)	64/120 8/23	(53.3%) (34.8%)	0.5 0.5	
**Cytogenetics (N = 106)**						
Pseudodiploid	5/12	(41.7%)	38/94	(40.4%)	1.0	
Pseudodiploid + Hypodiploid or Hyperdiploid with SCA	7/12	(58.3%)	71/94	(75.5%)	0.3	
**Molecular Markers/FISH (N = 132)**						
BCR-ABL1	0/20	(0%)	5/112	(4.5%)	1.0	
MLL/KMT2A rearranged	0/20	(0%)	4/112	(3.6%)	1.0	
TCF3-PBX1	3/20	(15%)	11/112	(9.8%)	0.4	
Hyperdiploid with Trisomy of chromosomes 4 and 10	0/20	(0%)	10/112	(8.9%)	0.4	
ETV6-RUNX1	0/20	(0%)	11/112	(9.8%)	0.2	
ETV6-RUNX1 + Trisomy of chromosomes 4 and 10	0/20	(0%)	21/112	(18.8%)	0.04	

**Table 2:** Significantly Affected Reactome Pathways in CD22E12^low^ B-ALL and CD22ΔE12-Tg mice.

Reactome Pathway	Enrichment Score in CD22E12^low^ B-ALL		Enrichment Score in CD22ΔE12-Tg Mice	
	**NES**	***p*-Value**	**NES**	***p*-Value**
**Reactomes Involved in Transcription**				
mRNA 3’-end processing	2.4	2.8 × 10^−5^	2.4	1.3 × 10^−5^
RNA polymerase II transcription termination	2.3	2.8 × 10^−5^	2.5	1.3 × 10^−5^
Transport of mature mRNA derived from an intronless transcript	2.1	2.7 × 10^−5^	2.4	1.4 × 10^−5^
RNA polymerase II pretranscription events	1.8	4.7 × 10^−4^	2.6	1.2 × 10^−5^
RNA polymerase II transcription elongation	1.7	1.6 × 10^−3^	2.4	1.3 × 10^−5^
Transcriptional regulation by small RNAs	1.5	9.9 × 10^−3^	2.4	1.3 × 10^−5^
Positive epigenetic regulation of rRNA expression	1.5	1.9 × 10^−2^	2.0	1.3 × 10^−5^
**Reactomes Involved in mRNA Processing**				
Regulation of mRNA stability by proteins that bind AU-rich elements	1.8	2.4 × 0^−4^	2.5	1.2 × 10^−5^
mRNA splicing—minor pathway	1.6	1.3 × 10^−2^	2.3	1.3 × 10^−5^
tRNA processing in the nucleus	1.5	2.3 × 10^−2^	2.5	1.3 × 10^−5^
Metabolism of non-coding RNA	1.5	3.0 × 10^−2^	2.6	1.3 × 10^−5^
**Reactomes Involved in mRNA Transport**				
Transport of mature transcript to cytoplasm	2.6	3.0 × 10^−5^	2.6	1.2 × 10^−5^
Transport of mature mRNA derived from an intron-containing transcript	2.5	2.9 × 10^−5^	2.6	1.3 × 10^−5^
Transport of mature mRNAs’ intronless transcripts	2.2	2.7 × 10^−5^	2.4	1.3 × 10^−5^
**Reactomes Involved in Translation**				
Formation of a pool of free 40S subunits	3.0	3.1 × 10^−5^	2.8	1.2 × 10^−5^
Eukaryotic translation elongation	3.0	3.0 × 10^−5^	2.8	1.2 × 10^−5^
Peptide chain elongation	2.9	3.0 × 10^−5^	2.7	1.2 × 10^−5^
Eukaryotic translation termination	2.6	3.0 × 10^−5^	2.7	1.2 × 10^−5^
Ribosomal scanning and start codon recognition	2.6	2.8 × 10^−5^	2.6	1.3 × 10^−5^
Translation initiation complex formation	2.6	2.8 × 10^−5^	2.6	1.3 × 10^−5^
Activation of the mRNA upon binding of the cap-binding complex and eIFs and subsequent binding to 43S	2.4	2.8 × 10^−5^	2.6	1.3 × 10^−5^
Formation of the ternary complex and, subsequently, the 43S complex	2.4	2.7 × 10^−5^	2.5	1.3 × 10^−5^
**Reactomes Involved in Post-Translational Protein Modification**				
SUMOylation of RNA binding proteins	2.2	2.7 × 10^−5^	2.4	1.3 × 10^−5^
Synthesis of active ubiquitin: roles of E1/E2 enzymes	2.2	2.5 × 10^−5^	2.0	1.8 × 10^−4^
SUMOylation of SUMOylation proteins	2.1	7.7 × 10^−5^	2.3	1.4 × 10^−5^
SUMOylation of DNA replication proteins	2.0	1.1 × 10^−4^	2.4	1.3 × 10^−5^
SUMOylation of ubiquitinylation proteins	2.0	2.9 × 10^−4^	2.3	1.4 × 10^−5^
Protein ubiquitination	2.0	8.6 × 10^−5^	2.3	1.3 × 10^−5^
SUMOylation of transcription cofactors	1.9	3.2 × 10^−4^	1.9	7.9 × 10^−5^
SUMOylation of chromatin organization proteins	1.8	1.1 × 10^−3^	2.3	1.3 × 10^−5^
SUMOylation of DNA damage response and repair proteins	1.6	7.1 × 10^−3^	2.4	1.2 × 10^−5^
**Reactomes Involved in Signal Transduction**				
RAF activation	2.2	2.6 × 10^−5^	1.7	8.7 × 10^−3^
MAP kinase activation	1.9	2.5 × 10^−4^	1.5	1.5 × 10^−2^
RHOBTB2 GTPase cycle	1.9	2.1 × 10^−3^	1.8	2.7 × 10^−3^
Regulation of RAS by GAPs	1.9	2.6 × 10^−4^	2.0	1.2 × 10^−5^
MAPK6/MAPK4 signaling	1.8	4.5 × 10^−4^	2.1	1.2 × 10^−5^
**Reactomes Involved in Cell Cycle Pathway**				
Postmitotic nuclear pore complex (NPC) reformation	1.9	1.5 × 10^−3^	2.1	4.2 × 10^−5^
Nuclear envelope (NE) reassembly	1.8	9.7 × 10^−4^	2.2	1.2 × 10^−5^
Mitotic telophase/cytokinesis	1.8	1.2 × 10^−2^	1.9	1.3 × 10^−5^
Regulation of apoptosis	1.7	3.8 × 10^−3^	2.2	1.3 × 10^−5^
Establishment of sister chromatid cohesion	1.7	2.4 × 10^−2^	1.9	1.3 × 10^−3^
Nuclear pore complex (NPC) disassembly	1.7	7.9 × 10^−3^	2.3	1.4 × 10^−5^
Nuclear envelope breakdown	1.6	1.3 × 10^−2^	2.3	1.3 × 10^−5^
Amplification of signal from unattached kinetochores via a MAD2 inhibitory signal	1.5	1.3 × 10^−2^	2.7	1.2 × 10^−5^
Amplification of signal from the kinetochores	1.5	1.3 × 10^−2^	2.7	1.2 × 10^−5^
APC/C-mediated degradation of cell cycle proteins	1.5	1.6 × 10^−2^	2.4	1.2 × 10^−5^
Regulation of mitotic cell cycle	1.5	1.6 × 10^−2^	2.4	1.2 × 10^−5^

Enrichment scores are presented for each of the reactome gene sets selectively dysregulated in B-ALL cells from CD22ΔE12-Tg mice (in comparison to B-ALL cells from Eμ-MYC Tg and BCR-ABL Tg mice) and primary leukemia cells from CD22E12^low^ patients (in comparison to primary leukemia cells from other B-ALL patients). The non-parametric GSEA enrichment scores yielded significantly overexpressed reactomes, and Wald statistics were utilized to identify overexpressed genes. See Figure 3, Figures S3–S7 and Tables S2–S8 for details.

## Data Availability

We used the publicly available archived gene expression profiling datasets GSE13159, GSE11877, and GSE13351, which were generated in the GeneChip Human Genome U133 Plus 2.0 Array platform (ThermoFischer Scientific, Waltham, MA, USA), to examine the relative expression levels of CD22 exons 11−14 in primary leukemia cells from 421 newly diagnosed B-ALL patients. We downloaded the RNAseq data from the Therapeutically Applicable Research to Generate Effective Treatments (TARGET) program (https://target-data.nci.nih.gov/Public/ALL/mRNA-seq/Phase2/L3/expression/BCCA/ accessed on 28 January 2022). The data files with exon-level quantification for the CD22 exons 11, 12, 13, and 14 were manually downloaded from the TARGET repository. The clinical outcome data were retrieved from the TARGET clinical annotation files TARGET_ALL_ClinicalData_Phase_II_Discovery_20211118.xlsx and TARGET_ALL_ClinicalData_Phase_II_Validation_20211118.xlsx. Our previously reported archived datasets on gene expression profiles (GSE58874 and GSE58872) and phosphoproteomes (GSE58873 and GSE58874) of B-ALL cells from CD22ΔE12-Tg mice were used for comparison in our efforts to identify the dysregulated reactome pathways in CD22E12^low^ B-ALL patients. The original contributions presented in the study are included in the article/Supplementary Material. Further inquiries can be directed to the corresponding author.

## Supplementary Materials

The following supporting information can be downloaded at: https://www.mdpi.com/article/10.3390/cancers15051599/s1, Figure S1: Identification of B-ALL patients with low CD22 Exon 2 (CD22E2) expression, Figure S2: Newly diagnosed B-ALL patients exhibited similar expression of CD22 exon 2 in CD22E12^low^ and all other patients, Figure S3: Significantly overexpressed transcription pathway genes in CD22E12^low^ B-ALL patients, Figure S4: Significantly overexpressed mRNA processing and transport pathway group of genes in CD22E12^low^ B-ALL patients, Figure S5: Significantly overexpressed translation and post-translational protein modification pathway genes in CD22E12^low^ B-ALL patients, Figure S6: Significantly overexpressed signal transduction pathway genes in CD22E12^low^ B-ALL patients, Figure S7: Significantly overexpressed cell cycle pathway group of genes in CD22E12^low^ B-ALL patients, Figure S8: Unfavorable impact of CD22DE12-associated selective reduction of CD22E12 expression on treatment outcomes in newly diagnosed high-risk B-ALL, Figure S9: CD22E12^low^ status in high-risk B-ALL as a poor prognostic indicator in univariate and multivariate Cox proportional hazards models comparing LFS and OS outcomes; Table S1: Patient characteristics, Table S2: Dysregulated expression of transcription pathway group of genes in CD22E12^low^ B-ALL patients, Table S3: Expression of mRNA processing pathway group of genes in CD22E12^low^ B-ALL patients, Table S4: Expression of mRNA_transport pathway group of genes in CD22E12^low^ B-ALL patients, Table S5: Expression of translation pathway group of genes in CD22E12^low^ B-ALL patients, Table S6: Expression of post-translational protein modification pathway group of genes in CD22E12^low^ B-ALL patients, Table S7: Expression of signal transduction pathway genes in CD22E12^low^ B-ALL patients, Table S8: Expression of cell cycle pathway genes in CD22E12^low^ B-ALL patients; Table S9: Comparison of the clinical features of the 141-patient RNAseq subset with those for the total TARGET B-ALL patient population.
