# Supplementary material for: CD22 Exon 12 Deletion as an Independent Predictor of Poor Treatment Outcomes in B-ALL
Source: Cancers (Basel). 2023 Mar 4;15(5):1599. doi: 10.3390/cancers15051599 (PMC10000517; doi:10.3390/cancers15051599)
Supplement: Supplementary file 1 [file cancers-15-01599-s001.zip › cancers-2193409-supplementary.pdf]

# CD22 Exon 12 Deletion as an Independent Predictor of Poor Treatment Outcomes in B-ALL

**Sanjive Qazi and Fatih M. Uckun**

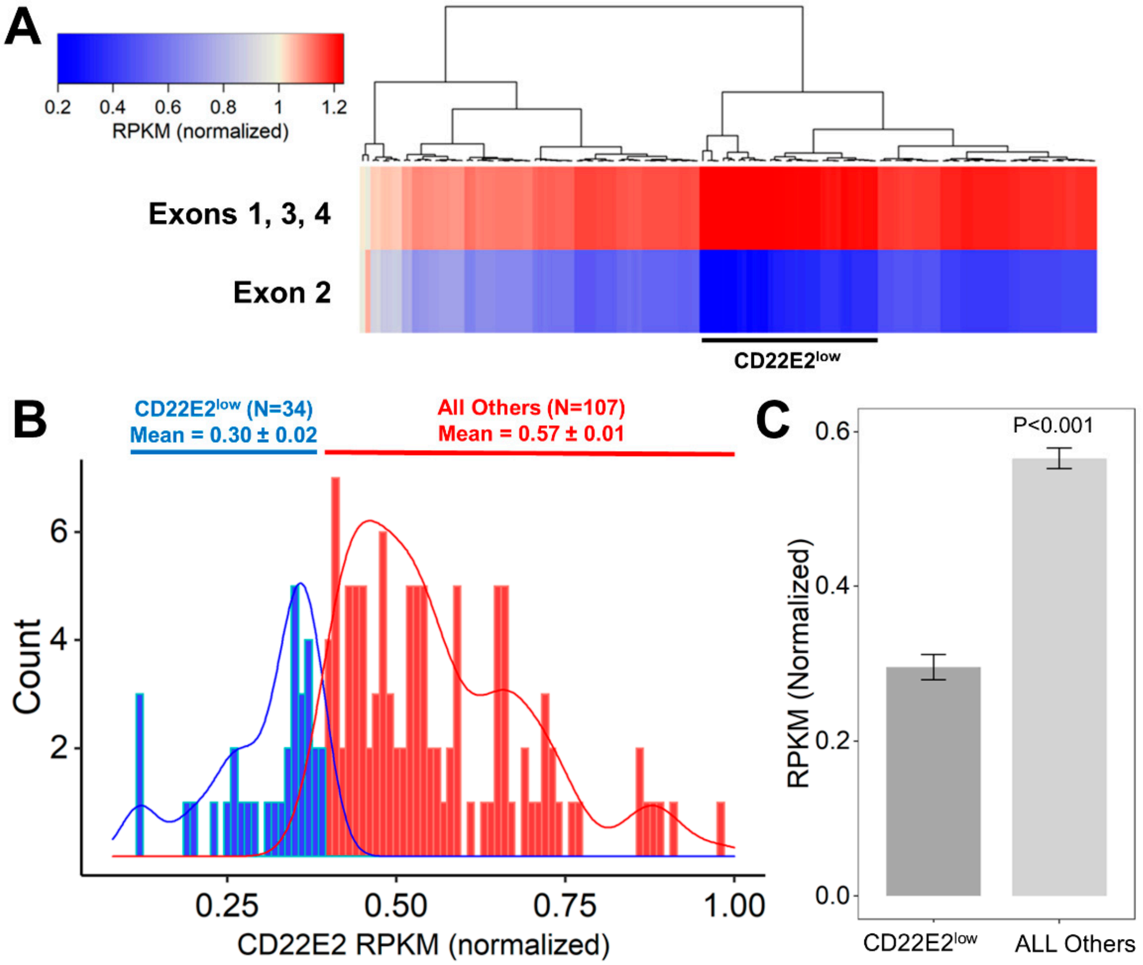


**Figure S1.** Identification of B-ALL patients with low CD22 Exon 2 (CD22E2) expression. [**A**] The cluster figure depicts the average normalized RPKM normalized values for CD22 exons 1, 3 and 4 (top row) and 2 (bottom row) for each patient (across columns). Blue color represents underexpression and red color overexpression relative to the mean-centered expressed of 4 exons per patient. The clustering algorithm (Euclidean distance and Wards linkage) identified 34 patients with low levels of CD22E2 expression relative to exons 1, 3 and 4 (black bar for CD22E2$^{low}$ patients). [**B**] Histogram depicts the distribution profile of normalized RPKM values for CD22E2 in CD22E2$^{low}$ patients (N=34) all of whom had normalized RPKM values of less than 0.397 (blue bars). The density plots depict 2 distribution profiles of 34 CD22E2$^{low}$ (blue line) and all other patients (red line). [**C**] CD22E2 expression level (in normalized RPKM) for the CD22E2$^{low}$ patients was 0.295 ± 0.016 (Median = 0.338; Range = 0 - 0.388), which was significantly lower than the mean CD22E2 expression level of 0.565 ± 0.014 (Median = 0.528; Range = 0.397 - 1.063) for the remaining all other 107 patients (Welch Two Sample T-test, T = 12.673, df = 83.3, P-value < $10^{-15}$).

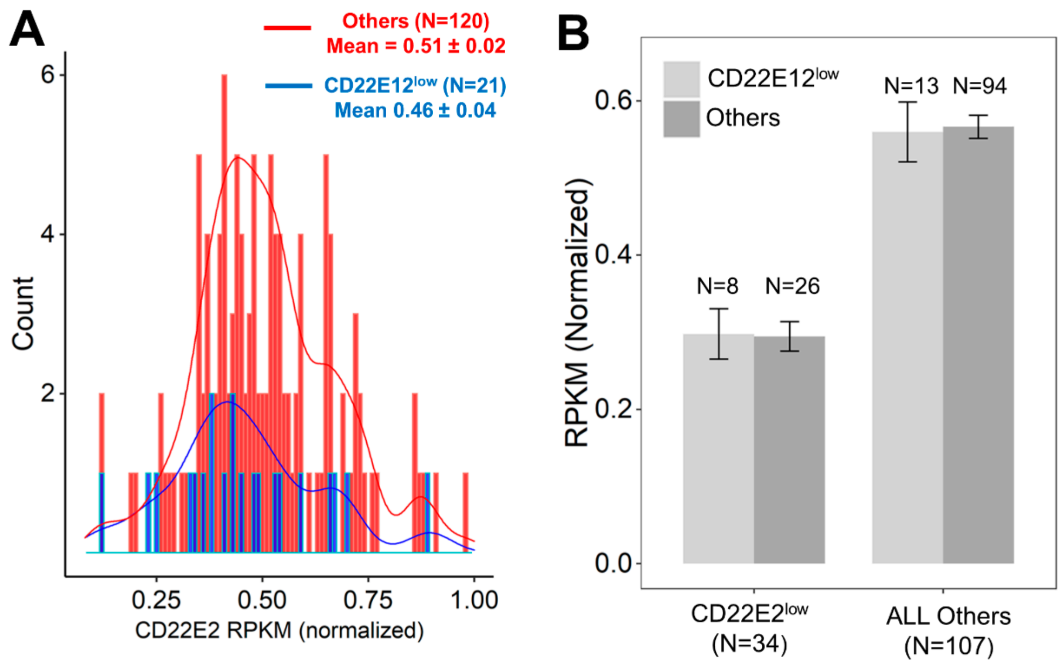


**Figure S2.** Newly diagnosed B-ALL patients exhibited similar expression of CD22 exon 2 in CD22E12$^{low}$ and all other patients. [**A**] Histogram depicts the distribution profile of normalized RPKM values for CD22E2 in CD22E12$^{low}$ patients (N=21; blue bars) and all other remaining patients (red bars; N=120). The density plots for CD22E2 mRNA expression for CD22E12$^{low}$ (blue line) vs. other patients (red line) displayed a near complete overlap. The mean CD22E2 expression level (in normalized RPKM) for the CD22E12$^{low}$ patients was 0.46 ± 0.039 (Median = 0.435; Range = 0.122 - 0.895), which was not significantly different from the mean CD22E2 expression level of 0.507 ± 0.016 (Median = 0.487; Range = 0 - 1.063) for the remaining 120 patients (Welch Two Sample T-test, T = 1.13, df = 27.2, P-value = 0.27). [**B**] CD22E2 RPKM expression values were examined in subgroups of 34 CD22E2$^{low}$ patients for 8 patients that were CD22E12$^{low}$ (light grey bar; Mean = 0.298 ± 0.032, Median = 0.332; Range = 0.122 - 0.385) versus 26 patients of the remaining others (dark grey bar; Mean = 0.295 ± 0.019, Median = 0.343; Range = 0 - 0.388), and in subgroups of 107 all other patients not identified as CD22E2$^{low}$ stratified according to 13 identified as CD22E12$^{low}$ (light grey bar; Mean = 0.56 ± 0.039, Median = 0.528; Range = 0.415 - 0.895)) and 94 other remaining patients (dark grey bar; Mean = 0.566 ± 0.015 (Median = 0.529; Range = 0.397 - 1.063)) showing that the mean expression was similar for CD22E12$^{low}$ versus others for both groups of CD22E2$^{low}$ and all other remaining patients not in the CD22E2$^{low}$ group (Two-way ANOVA, FDR adjusted P-value = 0.96).

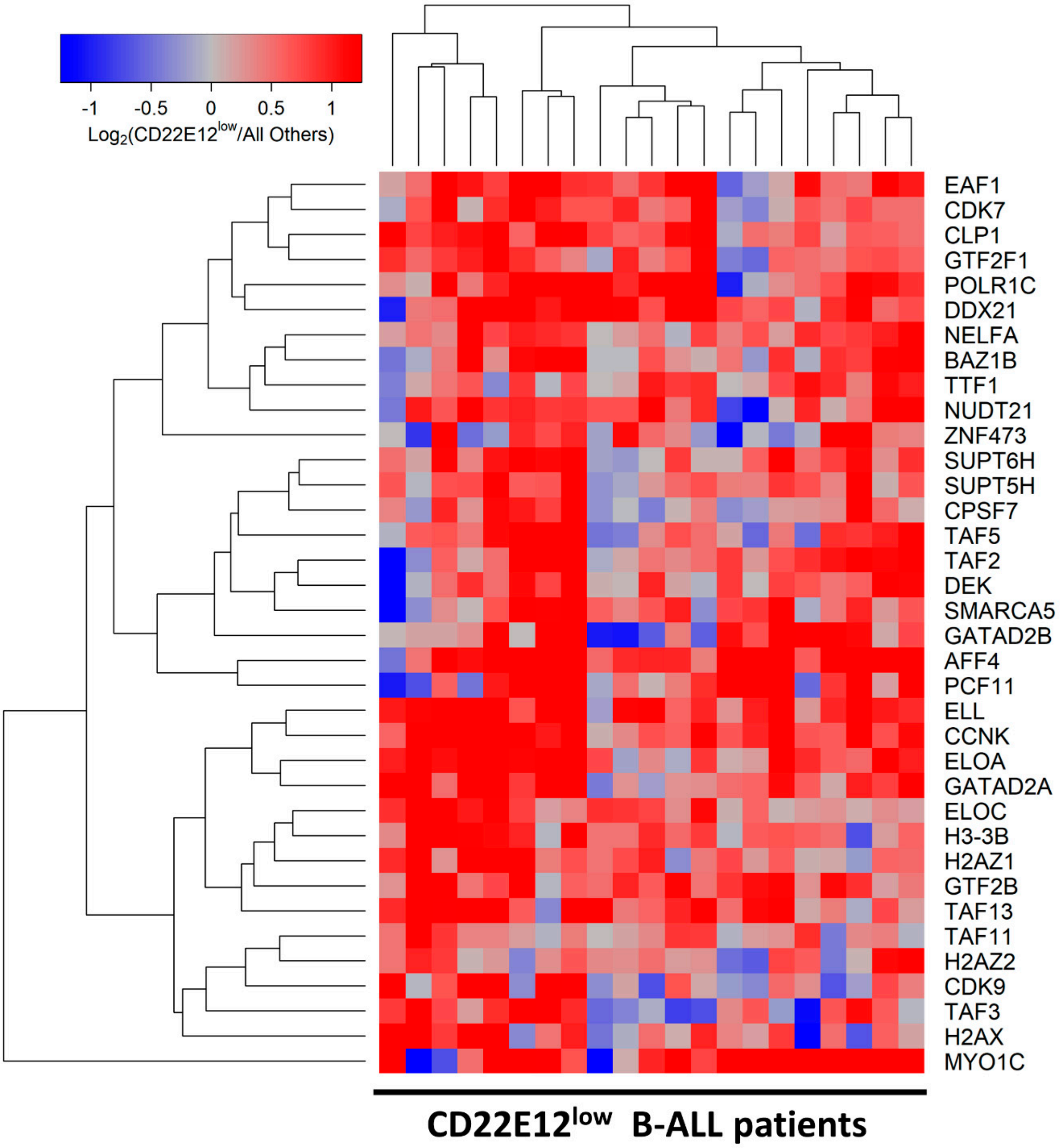


**Figure S3.** Significantly overexpressed transcription pathway genes in CD22E12$^{low}$ B-ALL patients. Reactomes involved in the transcription pathway included mRNA 3'-end processing, RNA Polymerase II transcription termination, transport of mature mRNA derived from an intronless transcript, RNA Polymerase II pre-transcription events, RNA Polymerase II transcription elongation, transcriptional regulation by small RNAs, and positive epigenetic regulation of rRNA expression were represented by 136 genes of which 75 genes exhibited fold change values greater than 1. All significantly upregulated as well as all significantly downregulated genes are shown in Table S2. With a P-value cutoff of 0.05, 45 genes were upregulated and 30 genes were downregulated in CD22E12$^{low}$ patients (**Table S2**). The depicted cluster figure displays the variance stabilized, normalized $log_2$ expression values in CD22E12$^{low}$ B-ALL patients mean-centered to the expression levels in all other patients (blue represents underexpression and red color represents overexpression in CD22E12$^{low}$ patients). The expression levels of co-regulated genes for both genes (rows) and patients (columns) are organized in the depicted dendrograms. Ranking according to Wald statistic (P-value<0.01) of the upregulated set of genes showed that GATA Zinc Finger Domain Containing 2A (GATAD2A)_ENSG00000167491 (Fold Increase = 1.9, P-value = 2.6 x $10^{-9}$), Nudix Hydrolase 21(NUDT21)_ENSG00000167005 (Fold Increase = 1.6; P-value = 7.3 x $10^{-9}$), cyclin K (CCNK)_ENSG00000090061 (Fold Increase = 1.8; P-value = 3.0 x $10^{-8}$) and negative elongation factor complex member A (NELFA)_ENSG00000185049 (Fold Increase = 1.5; P-value = 2.6 x $10^{-7}$) were the most differentially overexpressed genes in CD22E12$^{low}$ patients. The expression levels for the downregulated genes are shown in Table S2.

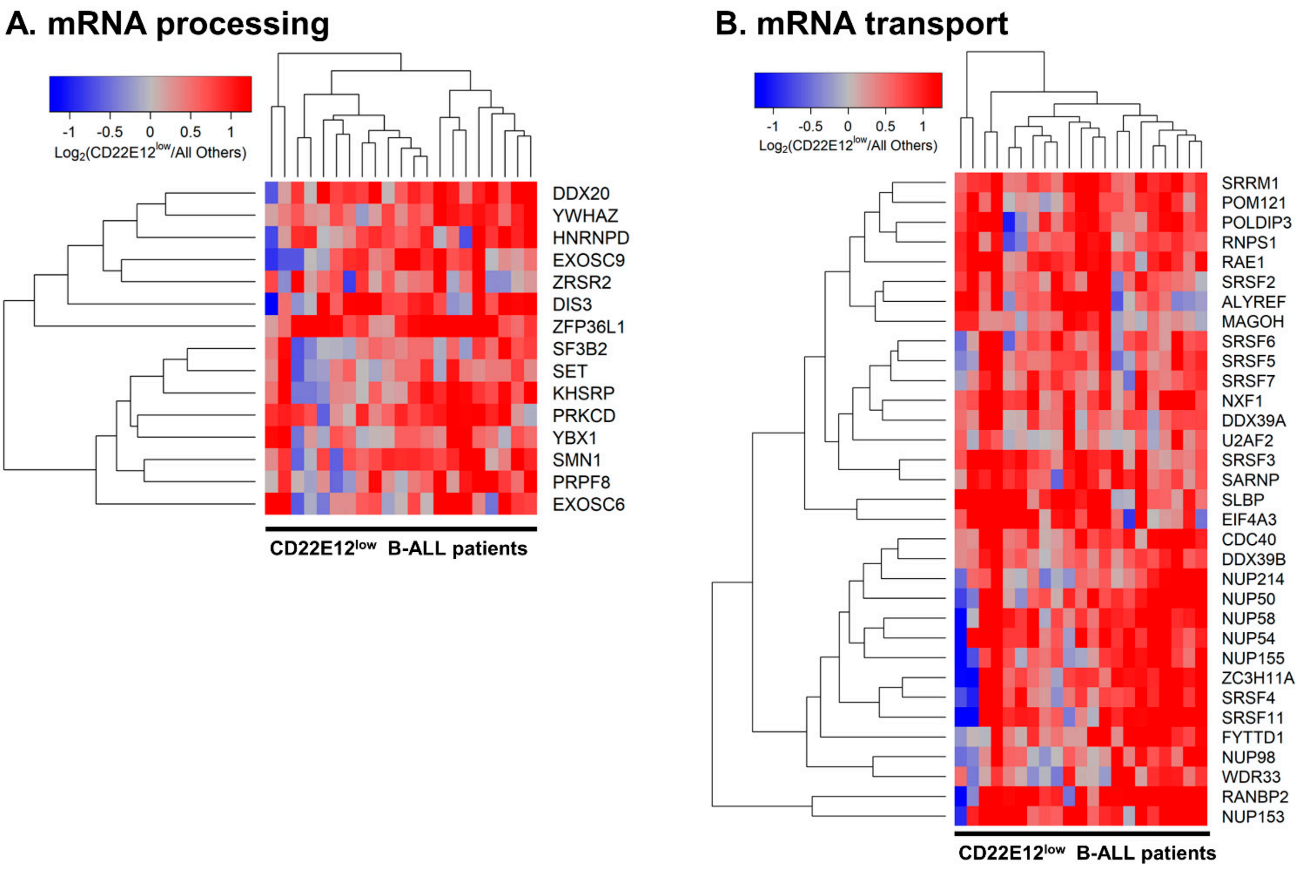


**Figure S4.** Significantly overexpressed mRNA processing and transport pathway group of genes in CD22E12$^{low}$ B-ALL patients. The depicted cluster figures in [A] and [B] display the variance stabilized, normalized log$_2$ expression values of the indicated pathway genes in CD22E12$^{low}$ B-ALL patients mean centered to the expression levels in all other patients (blue represents underexpression and the red color represents overexpression in CD22E12$^{low}$ patients). The expression levels of co-regulated genes for both genes (rows) and patients (columns) are organized in the depicted dendrograms. [**A**] Reactomes involved in mRNA processing pathway were represented by 94 genes of which 47 genes exhibited fold change values greater than 1. With a P-value cutoff of 0.05, 22 genes were upregulated and 25 genes were downregulated in CD22E12$^{low}$ patients (**Table S3**). Ranking according to Wald statistic (P-value<0.01) of the upregulated set of genes showed that survival of motor neuron 1 (SMN1)_ENSG00000172062 (Fold Increase = 1.6, P-value = 1.6 x 10$^{-6}$), KH-type spicing regulatory protein (KHSRP)_ENSG00000088247 (Fold Increase = 1.5; P-value = 1.5 x 10$^{-5}$), heterogeneous nuclear ribonucleoprotein a/b/d (HNRNPD)_ENSG00000138668 (Fold Increase = 1.5; P-value = 2.5 x 10$^{-5}$) and DEAD-Box Helicase 20 (DDX20)_ENSG00000064703 (Fold Increase = 1.6; P-value = 3.5 x 10$^{-5}$) were the most differentially overexpressed genes in CD22E12$^{low}$ patients. [**B**] Reactomes involved in the mRNA transport pathway were represented by 78 genes of which 55 genes exhibited fold change values greater than 1. With a P-value cutoff of 0.05, 38 genes were upregulated and 8 genes were downregulated in CD22E12$^{low}$ patients (**Table S4**). Ranking according to Wald statistic (P-value<0.01) of the upregulated set of genes showed that ribonucleic acid export 1(RAE1)_ENSG00000101146 (Fold Increase = 1.9, P-value = 1.1 x 10$^{-10}$), Polymerase delta-interacting protein 3 (POLDIP3)_ENSG00000100227 (Fold Increase = 1.6; P-value = 3.8 x 10$^{-8}$), Serine/arginine repetitive matrix protein 1 (SRRM1)_ENSG00000133226 (Fold Increase = 1.7; P-value = 4.3 x 10$^{-8}$) and Serine/arginine-rich splicing factor 3 (SRSF3)_ENSG00000112081 (Fold Increase = 1.7; P-value = 1.6 x 10$^{-7}$) were the most differentially overexpressed genes in CD22E12$^{low}$ patients. .

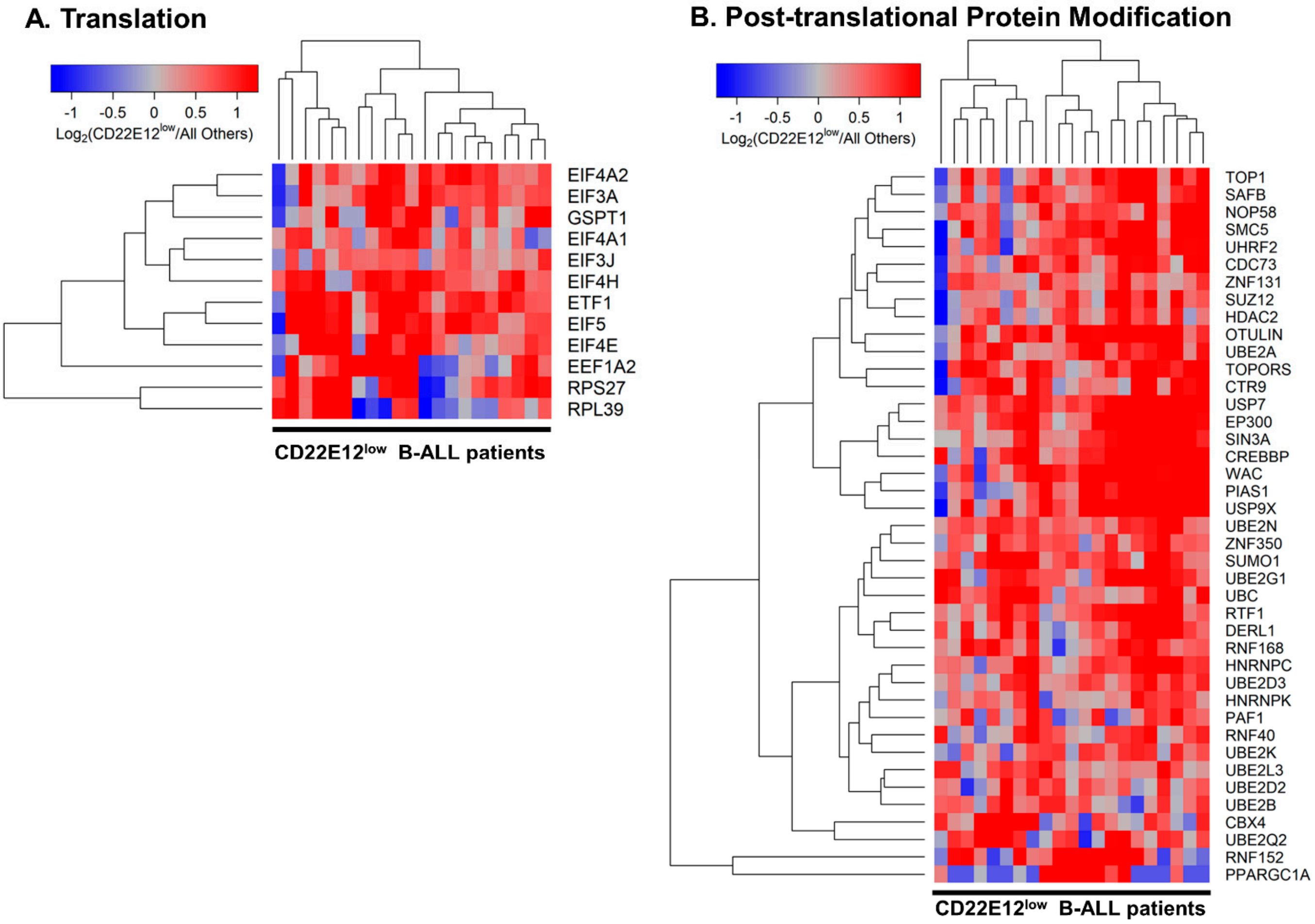


**Figure S5.** Significantly overexpressed translation and post-translational protein modification pathway genes in CD22E12$^{low}$ B-ALL patients. The depicted cluster figures in [A] and [B] display the variance stabilized, normalized $log_2$ expression values of the indicated pathway genes in CD22E12$^{low}$ B-ALL patients mean centered to the expression levels in all other patients (blue represents underexpression and the red color represents overexpression in CD22E12$^{low}$ patients). The expression levels of co-regulated genes for both genes (rows) and patients (columns) are organized in the depicted dendrograms. **[A]** Reactomes involved in the translation pathway were represented by 123 genes of which 111 genes exhibited fold change values greater than 1. With a P-value cutoff of 0.05, 26 genes were upregulated and 4 genes were downregulated in CD22E12$^{low}$ patients (**Table S5**). Ranking according to Wald statistic (P-value<0.01) of the upregulated set of genes showed that eukaryotic translation initiation factor 4H (EIF4H)_ENSG00000106682 (Fold Increase = 1.7, P-value = 1.4 x $10^{-7}$), eukaryotic translation initiation factor 4E (EIF4E)_ENSG00000151247 (Fold Increase = 1.8; P-value = 5.5 x $10^{-6}$), eukaryotic translation initiation factor 5(EIF5)_ENSG00000100664 (Fold Increase = 1.7; P-value = 5.0 x $10^{-5}$) and eukaryotic peptide chain release factor subunit 1 (ETF1)_ENSG00000120705 (Fold Increase = 1.7; P-value = 1.2 x $10^{-4}$) were the most differentially overexpressed translation pathway genes in CD22E12$^{low}$ patients. **[B].** Reactomes involed in the post-translational protein modification pathway were represented by 164 genes of which 96 genes exhibited fold change values greater than 1. With a P-value cutoff of 0.05, 54 genes were upregulated and 30 genes were downregulated in CD22E12$^{low}$ patients (**Table S6**). Ranking according to Wald statistic (P-value<0.01) of the upregulated set of genes showed that RNA polymerase-associated protein RTF1 homolog (RTF1)_ENSG00000137815 (Fold Increase = 1.8, P-value = 1.7 x $10^{-13}$), CREB-binding protein (CREBBP)_ENSG00000005339 (Fold Increase = 2.2; P-value = 3.5 x $10^{-11}$), Ubiquitin-conjugating enzyme E2 G1(UBE2G1)_ENSG00000132388 (Fold Increase = 1.8; P-value = 4.5 x $10^{-11}$) and histone acetyltransferase p300 (EP300)_ENSG00000100393 (Fold Increase = 2.1; P-value = 7.0 x $10^{-10}$) were the most differentially overexpressed genes in CD22E12$^{low}$ patients.

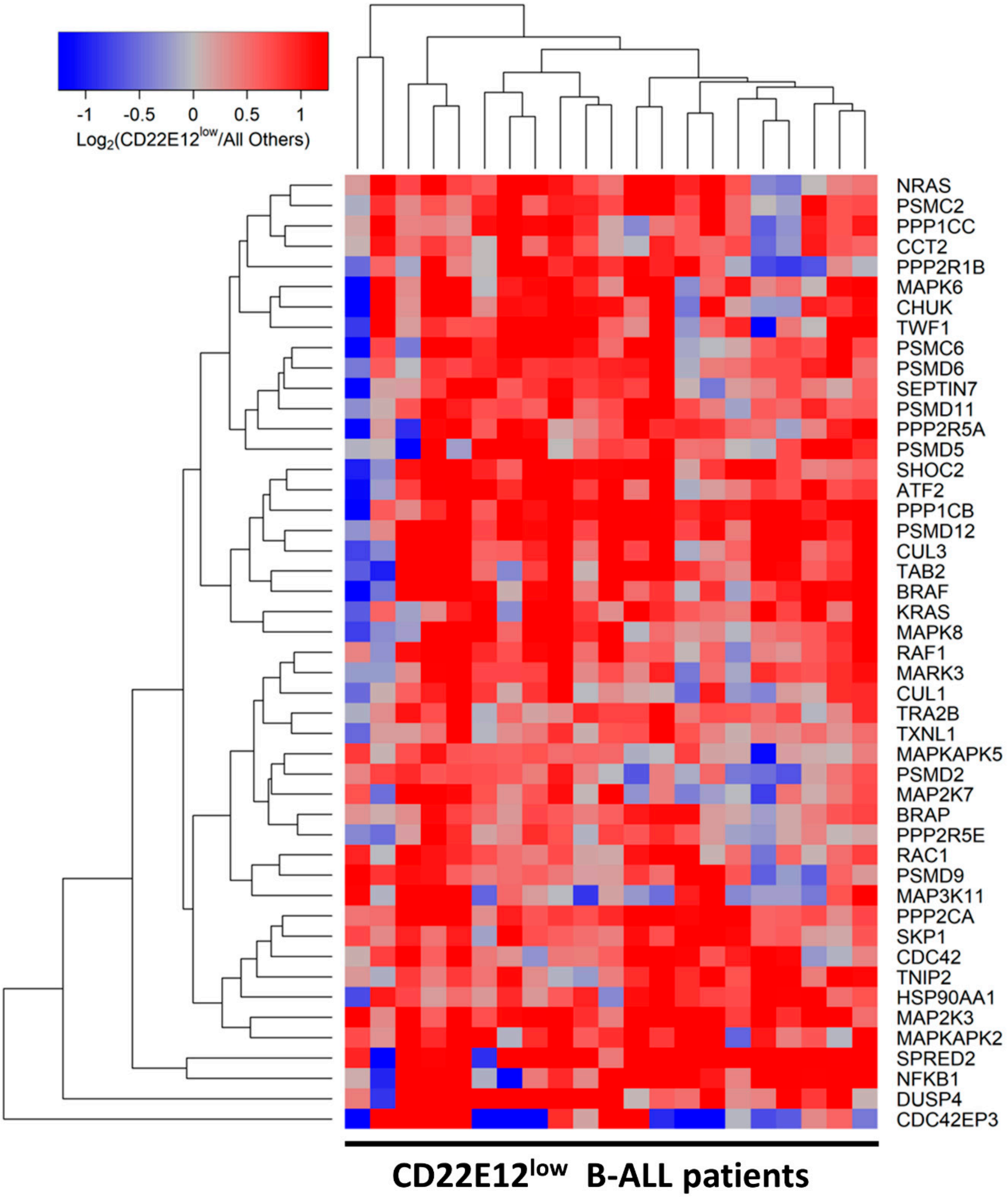


**Figure S6.** Significantly overexpressed signal transduction pathway genes in CD22E12$^{low}$ B-ALL patients. Reactomes involved in the signal transduction pathway were represented by 200 genes of which 127 genes exhibited fold change values greater than 1. With a P-value cutoff of 0.05, 70 genes were upregulated and 24 genes were downregulated in CD22E12$^{low}$ patients (**Table S7**). The depicted cluster figure displays the variance stabilized, normalized $\log_2$ expression values of the indicated pathway genes in CD22E12$^{low}$ B-ALL patients mean centered to the expression levels in all other patients (blue represents underexpression and red color represents overexpression in CD22E12$^{low}$ patients). The expression levels of co-regulated genes for both genes (rows) and patients (columns) are organized in the depicted dendrograms. Ranking according to Wald statistic (P-value<0.01) of the upregulated set of genes showed that serine/threonine-protein phosphatase 2A catalytic subunit alpha isoform (PPP2CA)_ENSG00000113575 (Fold Increase = 1.9, P-value = 3.3 x $10^{-11}$), 26S proteasome regulatory subunit 7 (PSMC2)_ENSG00000161057 (Fold Increase = 1.6; P-value = 8.3 x $10^{-10}$), S-phase kinase-associated protein 1 (SKP1)_ENSG00000113558 (Fold Increase = 1.6; P-value = 1.5 x $10^{-8}$) and Nuclear factor NF-kappa-B p105 subunit (NFKB1)_ENSG00000109320 (Fold Increase = 2.3; P-value = 2.1 x $10^{-7}$) were the most differentially overexpressed genes in CD22E12$^{low}$ patients.

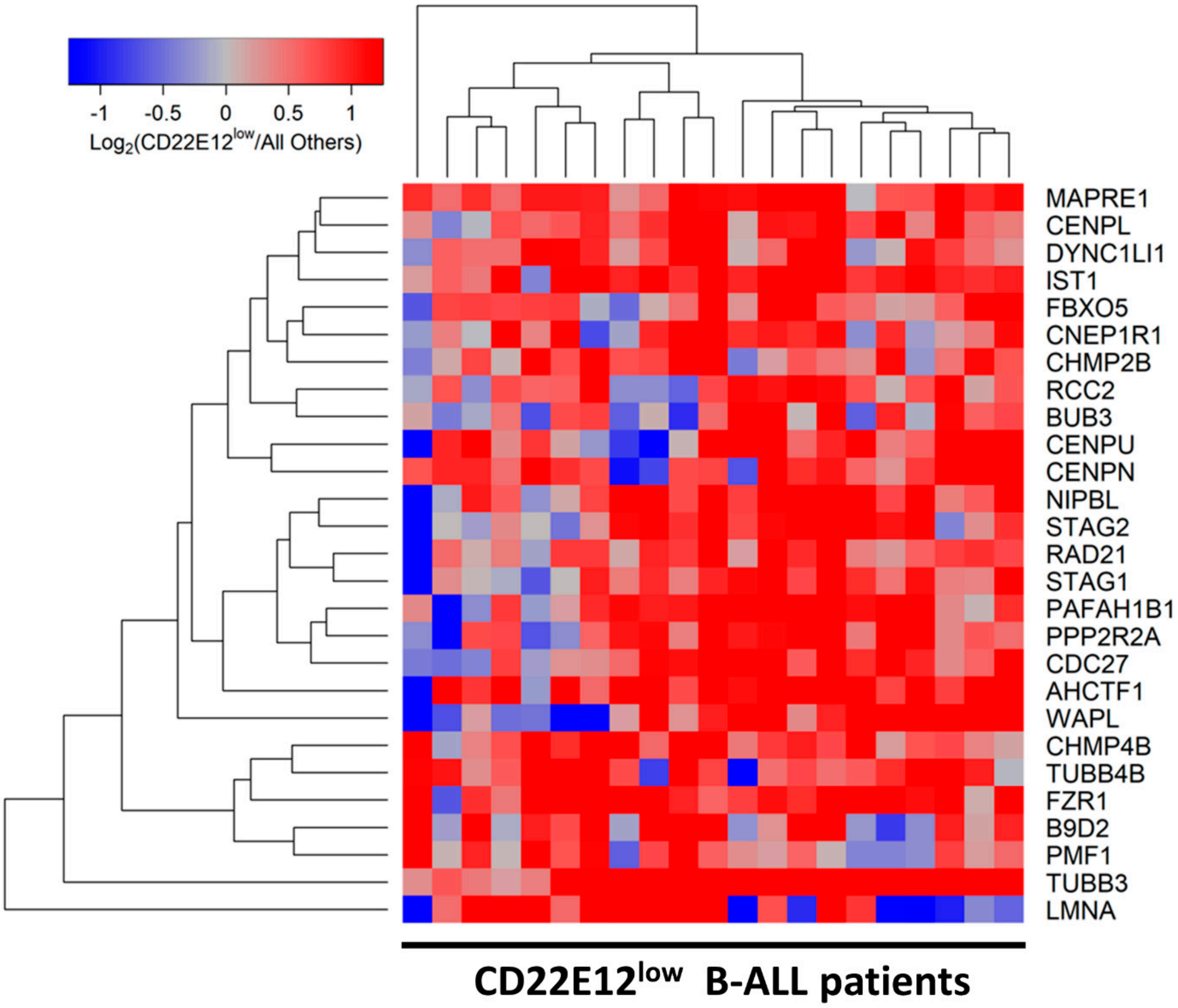


**Figure S7.** Significantly overexpressed Cell Cycle pathway group of genes in CD22E12$^{low}$ B-ALL patients Reactomes involved in the cell cycle pathway were represented by 160 genes of which 92 genes exhibited fold change values greater than 1. With a P-value cutoff of 0.05, 39 genes were upregulated and 32 genes were downregulated in CD22E12$^{low}$ patients (**Table S8**). The depicted cluster figure displays the variance stabilized, normalized $\log_2$ expression values of the indicated cell cycle pathway genes in CD22E12$^{low}$ B-ALL patients mean centered to the expression levels in all other patients (blue represents underexpression and red color represents overexpression in CD22E12$^{low}$ patients). The expression levels of co-regulated genes for both genes (rows) and patients (columns) are organized in the depicted dendrograms. Ranking according to Wald statistic (P-value<0.01) of the upregulated set of genes showed that cytoplasmic dynein 1 light intermediate chain 1 (DYNC1LI1)_ENSG00000144635 (Fold Increase = 1.7, P-value = 5.5 x $10^{-10}$), Microtubule-associated protein RP/EB family member 1 (MAPRE1)_ENSG00000101367 (Fold Increase = 1.8; P-value = 7.2 x $10^{-8}$), Fizzy-related protein homolog (FZR1)_ENSG00000105325 (Fold Increase = 2.0; P-value = 1.2 x $10^{-6}$) and charged multivesicular body protein 4b (CHMP4B)_ENSG00000101421 (Fold Increase = 1.8; P-value = 6.6 x $10^{-6}$) were the most differentially overexpressed genes in CD22E12$^{low}$ patients.

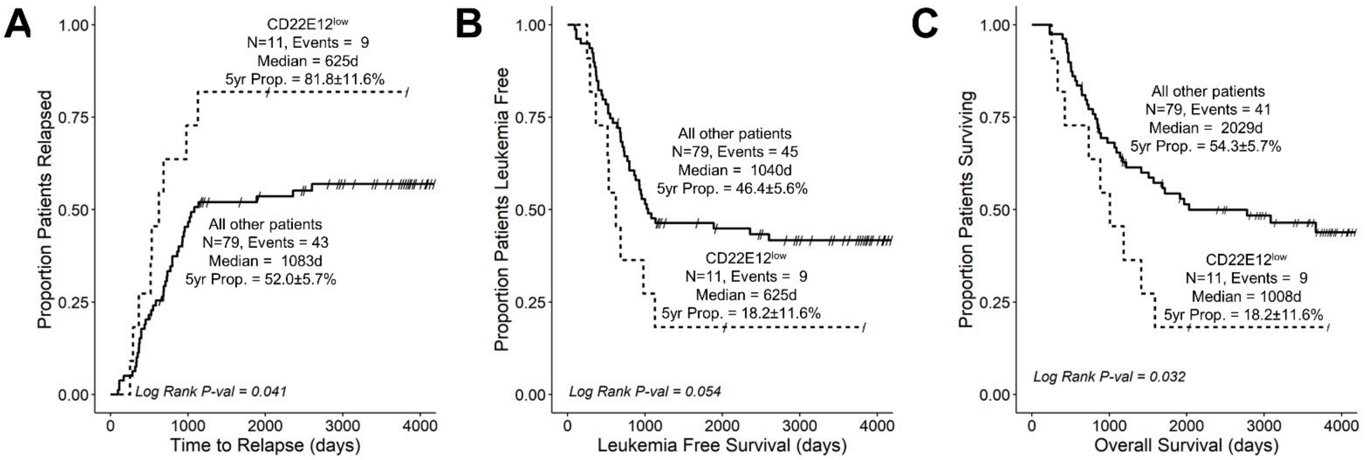


**Figure S8.** Unfavorable impact of CD22DE12-associated selective reduction of CD22E12 expression on treatment outcomes in newly diagnosed high-risk B-ALL. Treatment outcomes were compared using Kaplan Meier analysis for CD22E12$^{low}$ (N=11) vs. other patients (N=79) within the high-risk subset (N=90). [A] Relapse curves. [B] LFS curves. [C] OS curves. CD22E12$^{low}$ patients exhibited a higher probability of relapse, shorter time to relapse, worse LFS with shorter times to first event (relapse or death) and worse OS. See text for a discussion of results.

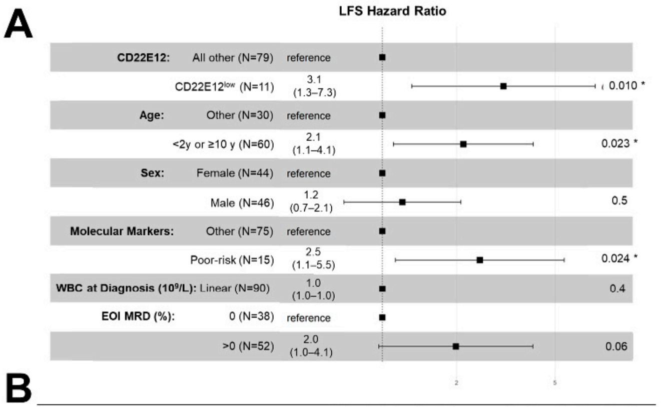


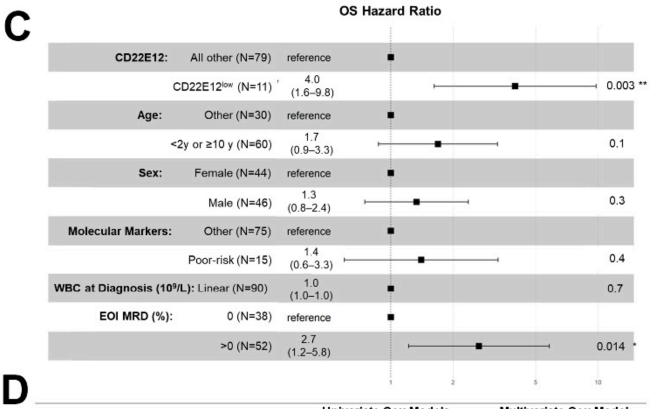


**B**

| Factor | B-ALL subset | Univariate Cox Models | | Multivariate Cox Model | |
|---|---|---|---|---|---|
| | | HR (95% CI) | P-value | HR (95% CI) | P-value |
| CD22E12 | CD22E12$^{low}$ | 2 .0 (1.0 - 4.1) | 0.06 | 3.1 (1.3 - 7.3) | 0.010 |
| Age | <2y or ≥10y | 1.9 (1.0 - 3.5) | 0.037 | 2.1 (1.1 - 4.1) | 0.023 |
| Sex | Male | 1.2 (0.7 - 2.1) | 0.5 | 1.2 (0.7 - 2.1) | 0.5 |
| Molecular Markers | Poor-risk | 2.5 (1.4 - 4.8) | 0.004 | 2.5 (1.1 - 5.5) | 0.024 |
| WBC at Diagnosis ($10^9$/L) | Linear | 1.0 (1.0 - 1.0) | 0.2 | 1.0 (1.0 - 1.0) | 0.4 |
| EOI MRD (%) | >0 | 1.9 (1.1 - 3.3) | 0.035 | 2.0 (1.0 - 4.1) | 0.06 |

**D**

| Factor | B-ALL subset | Univariate Cox Models | | Multivariate Cox Model | |
|---|---|---|---|---|---|
| | | HR (95% CI) | P-value | HR (95% CI) | P-value |
| CD22E12 | CD22E12$^{low}$ | 2.2 (1.1 - 4.5) | 0.036 | 4.0 (1.6 - 9.8) | 0.003 |
| Age | <2y or ≥10y | 1.8 (1.0 - 3.5) | 0.06 | 1.7 (0.9 - 3.3) | 0.1 |
| Sex | Male | 1.4 (0.8 - 2.4) | 0.3 | 1.3 (0.8 - 2.4) | 0.3 |
| Molecular Markers | Poor-risk | 1.6 (0.8 - 3.3) | 0.2 | 1.4 (0.6 - 3.3) | 0.4 |
| WBC at Diagnosis ($10^9$/L) | Linear | 1.0 (1.0 - 1.0) | 0.8 | 1.0 (1.0 - 1.0) | 0.7 |
| EOI MRD (%) | >0 | 1.9 (1.0 - 3.4) | 0.046 | 2.7 (1.2 - 5.8) | 0.014 |

**Figure S9.** CD22E12$^{low}$ status in high-risk B-ALL as a poor prognostic indicator in univariate and multivariate Cox proportional hazards models comparing the LFS and OS outcomes. We examined in 90 newly diagnosed high-risk B-ALL patients the effects of CD22E12$^{low}$ status as a poor prognostic indicator that is associated with worse LFS and OS outcomes in univariate and multivariate Cox proportional hazards models that included as variables the following candidate poor prognostic characteristics for hazard ratio (HR) determinations: (i) CD22E12$^{low}$ status; (ii) Age<2 y or ≥10 y; (iii) Male; (iv) poor-risk classification based on molecular markers (BCR-ABL1$^{+}$, MLL-R$^{+}$ or TCF3-PBX1$^{+}$); (v) WBC at diagnosis (as a linear covariate), and (vi) end-of-induction day 29 MRD burden >0 (MRD≥0.001 %). [A] Depicted are the Forest plots for LFS along with the corresponding HRs and *p*-values for each covariate in the multivariate Cox proportional hazards model. [B] The HR for LFS and corresponding P-value are provided for each variable in both the univariate and multivariate models. In the multivariate model, CD22E12$^{low}$ status was found to be a significant and independent predictor of poor LFS outcome: HR = 3.1 ± 0.4, P-value = 0.010). [C] Depicted are the Forest plots for OS along with the corresponding HRs and *p*-values for each covariate in the multivariate Cox proportional hazards model. [D] The HR for OS and corresponding P-value are provided for each variable in the univariate and multivariate models. CD22E12$^{low}$ status (HR = 2.2 ± 0.4, P-value = 0.04) and having a detectable (MRD>0) end of induction residual leukemia burden (HR = 1.9 ± 0.3, P-value = 0.046) were identified as predictors of poor OS. Both variables were identified as significant and independent predictors of poor OS outcome in the multivariate model (HR for CD22E12$^{low}$ status = 4.0 ± 0.5, P-value = 0.003; HR for EOI MRD>0 = 2.7 ± 0.4, P-value = 0.014).

**Table S1.** Patient Characteristics.

| Variable | Total Population (N=141) Mean/Median(Range) or N (% evaluable) | | CD22E12$^{low}$ (N=21) Mean/Median(Range) or N (% evaluable) | | All Others (N=120) Mean/Median(Range) or N (% evaluable) | |
|---|---|---|---|---|---|---|
| **Age (yrs)** | | | | | | |
| Mean±SEM / Median (Range) | 7.9 ± 0.5 | 6.5 (1.2 - 30) | 8.2 ± 1.2 | 7.6 (1.4 - 18.1) | 7.9 ± 0.5 | 6.4 (1.2 - 30) |
| **WBC (x10$^9$/L)** | | | | | | |
| Mean±SEM / Median (Range) | 72.2 ± 10.4 | 28.3 (1.1 - 1149) | 45.5 ± 13.1 | 15.9 (1.3 - 214.5) | 76.8 ± 12.01 | 33 (1.1 - 1149) |
| **Sex** | | | | | | |
| Female | 70 | (49.6%) | 10 | (47.6%) | 60 | (50%) |
| Male | 71 | (50.4%) | 11 | (52.4%) | 60 | (50%) |
| **Race** | | | | | | |
| Asian | 5 | (3.5%) | 0 | (0%) | 5 | (4.2%) |
| Black or African American | 17 | (12.1%) | 4 | (19%) | 13 | (10.8%) |
| Unknown | 22 | (15.6%) | 5 | (23.8%) | 17 | (14.2%) |
| White | 97 | (68.8%) | 12 | (57.1%) | 85 | (70.8%) |
| **Ethnicity** | | | | | | |
| Hispanic or Latino | 31 | (22%) | 8 | (38.1%) | 23 | (19.2%) |
| Not Hispanic or Latino | 99 | (70.2%) | 12 | (57.1%) | 87 | (72.5%) |
| Unknown | 11 | (7.8%) | 1 | (4.8%) | 10 | (8.3%) |
| **CNS Status at Diagnosis** | | | | | | |
| CNS 1 | 113 | (80.1%) | 16 | (76.2%) | 97 | (80.8%) |
| CNS 2 | 25 | (17.7%) | 5 | (23.8%) | 20 | (16.7%) |
| CNS 3 | 3 | (2.1%) | 0 | (0%) | 3 | (2.5%) |
| **NCI Risk** | | | | | | |
| High Risk | 90 | (63.8%) | 11 | (52.4%) | 79 | (65.8%) |
| Standard risk | 51 | (36.2%) | 10 | (47.6%) | 41 | (34.2%) |
| **Cytogenetics (N=106)** | | | | | | |
| Normal diploid | 16 | (15.1%) | 2 | (16.7%) | 14 | (14.9%) |
| Hyperdiploid with no SCA | 12 | (11.3%) | 3 | (25%) | 9 | (9.6%) |
| Hyperdiploid with SCA | 27 | (25.5%) | 1 | (8.3%) | 26 | (27.7%) |
| Hypodiploid with SCA | 8 | (7.5%) | 1 | (8.3%) | 7 | (7.4%) |
| Pseudodiploid | 43 | (40.6%) | 5 | (41.7%) | 38 | (40.4%) |
| Not determined. | 35 | | 9 | | 26 | |
| t(4;11) | 3 | (2.8%) | 0 | (0%) | 3 | (3.2%) |
| t(1:19) | 11 | (10.4%) | 2 | (16.7%) | 9 | (9.6%) |
| t(9;22) | 5 | (4.7%) | 0 | (0%) | 5 | (5.3%) |
| **Molecular markers/FISH (N=132)** | | | | | | |
| BCR-ABL1 | 5 | (3.8%) | 0 | (0%) | 5 | (4.5%) |
| ETV6-RUNX1 | 11 | (8.3%) | 0 | (0%) | 11 | (9.8%) |

| | | | | | | |
|---|---|---|---|---|---|---|
| Hyperdiploid | 15 | (11.4%) | 4 | (20%) | 11 | (9.8%) |
| iAMP21 | 3 | (2.3%) | 0 | (0%) | 3 | (2.7%) |
| MLL rearranged | 4 | (3%) | 0 | (0%) | 4 | (3.6%) |
| TCF3-HLF | 1 | (0.8%) | 0 | (0%) | 1 | (0.9%) |
| TCF3-PBX1 | 14 | (10.6%) | 3 | (15%) | 11 | (9.8%) |
| Trisomy of chromosomes 4 and 10 | 10 | (7.6%) | 0 | (0%) | 10 | (8.9%) |
| None of the above | 69 | (52.3%) | 13 | (65%) | 56 | (50%) |
| Not Determined | 9 | | 1 | | 8 | |

**Table S2.** Dysregulated expression of Transcription pathway group of genes in CD22E12$^{low}$ B-ALL patients.

| Gene | Fold Increase (CD22E12$^{low}$/All Others) | Wald Statistic (P-value) |
|---|---|---|
| GATAD2A_ENSG00000167491 | 1.88 | 2.6 x $10^{-9}$ |
| NUDT21_ENSG00000167005 | 1.6 | 7.3 x $10^{-9}$ |
| CCNK_ENSG00000090061 | 1.82 | 3.0 x $10^{-8}$ |
| NELFA_ENSG00000185049 | 1.52 | 2.6 x $10^{-7}$ |
| POLR1C_ENSG00000171453 | 1.86 | 3.8 x $10^{-7}$ |
| ELOA_ENSG00000011007 | 1.87 | 4.6 x $10^{-7}$ |
| CDK7_ENSG00000134058 | 1.57 | 9.8 x $10^{-7}$ |
| BAZ1B_ENSG00000009954 | 1.51 | 2.4 x $10^{-6}$ |
| GTF2F1_ENSG00000125651 | 1.45 | 7.4 x $10^{-6}$ |
| EAF1_ENSG00000144597 | 1.64 | 1.5 x $10^{-5}$ |
| CLP1_ENSG00000172409 | 1.59 | 1.7 x $10^{-5}$ |
| AFF4_ENSG00000072364 | 1.98 | 2.7 x $10^{-5}$ |
| ELOC_ENSG00000154582 | 1.52 | 3.2 x $10^{-5}$ |
| GTF2B_ENSG00000137947 | 1.55 | 4.1 x $10^{-5}$ |
| SUPT5H_ENSG00000196235 | 1.47 | 5.1 x $10^{-5}$ |
| TAF5_ENSG00000148835 | 1.63 | 7.2 x $10^{-5}$ |
| ELL_ENSG00000105656 | 1.74 | 8.5 x $10^{-5}$ |
| TTF1_ENSG00000125482 | 1.37 | 5.6 x $10^{-4}$ |
| TAF11_ENSG00000064995 | 1.28 | 8.4 x $10^{-4}$ |
| SUPT6H_ENSG00000109111 | 1.43 | 9.2 x $10^{-4}$ |
| PCF11_ENSG00000165494 | 1.65 | 1.6 x $10^{-3}$ |
| TAF2_ENSG00000064313 | 1.44 | 1.7 x $10^{-3}$ |
| H2AZ2_ENSG00000105968 | 1.27 | 2.3 x $10^{-3}$ |
| TAF13_ENSG00000197780 | 1.55 | 2.5 x $10^{-3}$ |
| SMARCA5_ENSG00000153147 | 1.42 | 2.5 x $10^{-3}$ |
| DDX21_ENSG00000165732 | 1.52 | 2.5 x $10^{-3}$ |
| H2AZ1_ENSG00000164032 | 1.42 | 2.5 x $10^{-3}$ |
| CPSF7_ENSG00000149532 | 1.29 | 3.1 x $10^{-3}$ |
| GATAD2B_ENSG00000143614 | 1.42 | 3.4 x $10^{-3}$ |
| ZNF473_ENSG00000142528 | 1.43 | 3.9 x $10^{-3}$ |
| DEK_ENSG00000124795 | 1.36 | 4.3 x $10^{-3}$ |
| H2AX_ENSG00000188486 | 1.52 | 4.9 x $10^{-3}$ |
| TAF3_ENSG00000165632 | 1.3 | 6.4 x $10^{-3}$ |
| H3-3B_ENSG00000132475 | 1.39 | 7.4 x $10^{-3}$ |
| CDK9_ENSG00000136807 | 1.3 | 7.7 x $10^{-3}$ |
| MYO1C_ENSG00000197879 | 1.73 | 7.9 x $10^{-3}$ |
| PAPOLA_ENSG00000090060 | 1.37 | 1.0 x $10^{-2}$ |
| CHD4_ENSG00000111642 | 1.3 | 1.2 x $10^{-2}$ |
| TAF4B_ENSG00000141384 | 1.44 | 1.4 x $10^{-2}$ |
| TAF7_ENSG00000178913 | 1.27 | 1.6 x $10^{-2}$ |
| H3-3A_ENSG00000163041 | 1.25 | 1.8 x $10^{-2}$ |
| TAF1B_ENSG00000115750 | 1.27 | 2.4 x $10^{-2}$ |
| NELFCD_ENSG00000101158 | 1.19 | 3.0 x $10^{-2}$ |

| | | |
|---|---|---|
| MTA2_ENSG00000149480 | 1.21 | $4.0 \times 10^{-2}$ |
| POLR2B_ENSG00000047315 | 1.29 | $4.8 \times 10^{-2}$ |
| GSK3B_ENSG00000082701 | 1.18 | $5.2 \times 10^{-2}$ |
| PABPN1_ENSG00000100836 | 1.15 | $6.2 \times 10^{-2}$ |
| POLR1D_ENSG00000186184 | 1.21 | $6.2 \times 10^{-2}$ |
| SNRPB_ENSG00000125835 | 1.29 | $6.2 \times 10^{-2}$ |
| SNRPF_ENSG00000139343 | 1.27 | $6.6 \times 10^{-2}$ |
| TAF7L_ENSG00000102387 | 1.46 | $9.7 \times 10^{-2}$ |
| CTDP1_ENSG00000060069 | 1.29 | $9.7 \times 10^{-2}$ |
| CCNT2_ENSG00000082258 | 1.25 | $1.4 \times 10^{-1}$ |
| POLR1F_ENSG00000105849 | 1.18 | $1.4 \times 10^{-1}$ |
| ERCC3_ENSG00000163161 | 1.1 | $1.7 \times 10^{-1}$ |
| CCNT1_ENSG00000129315 | 1.19 | $1.8 \times 10^{-1}$ |
| SNRPG_ENSG00000143977 | 1.14 | $1.9 \times 10^{-1}$ |
| SUPT4H1_ENSG00000213246 | 1.15 | $2.3 \times 10^{-1}$ |
| ELOB_ENSG00000103363 | 1.17 | $2.6 \times 10^{-1}$ |
| CBX3_ENSG00000122565 | 1.08 | $2.9 \times 10^{-1}$ |
| MTA1_ENSG00000182979 | 1.08 | $3.5 \times 10^{-1}$ |
| SNRPE_ENSG00000182004 | 1.13 | $3.7 \times 10^{-1}$ |
| CSTF2_ENSG00000101811 | 1.09 | $4.6 \times 10^{-1}$ |
| H2AJ_ENSG00000246705 | 1.12 | $4.8 \times 10^{-1}$ |
| GTF2F2_ENSG00000188342 | 1.07 | $4.8 \times 10^{-1}$ |
| TAF4_ENSG00000130699 | 1.07 | $4.8 \times 10^{-1}$ |
| RBBP7_ENSG00000102054 | 1.06 | $5.0 \times 10^{-1}$ |
| SUPT16H_ENSG00000092201 | 1.06 | $5.2 \times 10^{-1}$ |
| POLR1B_ENSG00000125630 | 1.06 | $5.7 \times 10^{-1}$ |
| TAF1C_ENSG00000103168 | 1.04 | $6.4 \times 10^{-1}$ |
| POLR2D_ENSG00000144231 | 1.04 | $6.8 \times 10^{-1}$ |
| CSTF1_ENSG00000101138 | 1.03 | $7.3 \times 10^{-1}$ |
| TAF1D_ENSG00000166012 | 1.04 | $7.9 \times 10^{-1}$ |
| KAT2B_ENSG00000114166 | 1.02 | $8.2 \times 10^{-1}$ |
| TCEA1_ENSG00000187735 | 1 | $9.9 \times 10^{-1}$ |
| TAF10_ENSG00000166337 | 1 | $1.0 \times 10^{0}$ |
| TAF1L_ENSG00000122728 | 0.98 | $9.1 \times 10^{-1}$ |
| POLR2J_ENSG00000005075 | 0.98 | $8.8 \times 10^{-1}$ |
| POLR2A_ENSG00000181222 | 0.99 | $8.8 \times 10^{-1}$ |
| POLR1A_ENSG00000068654 | 0.98 | $8.6 \times 10^{-1}$ |
| TBP_ENSG00000112592 | 0.97 | $6.9 \times 10^{-1}$ |
| POLR2K_ENSG00000147669 | 0.95 | $6.8 \times 10^{-1}$ |
| POLR2L_ENSG00000177700 | 0.92 | $6.4 \times 10^{-1}$ |
| POLR1E_ENSG00000137054 | 0.94 | $5.8 \times 10^{-1}$ |
| POLR1H_ENSG00000066379 | 0.93 | $5.8 \times 10^{-1}$ |
| POLR2C_ENSG00000102978 | 0.95 | $5.7 \times 10^{-1}$ |
| POLR2F_ENSG00000100142 | 0.93 | $5.4 \times 10^{-1}$ |
| IWS1_ENSG00000163166 | 0.94 | $4.9 \times 10^{-1}$ |
| CCNH_ENSG00000134480 | 0.88 | $4.2 \times 10^{-1}$ |
| MLLT3_ENSG00000171843 | 0.84 | $3.9 \times 10^{-1}$ |

| | | |
|---|---|---|
| TAF1A_ENSG00000143498 | 0.89 | $3.7 \times 10^{-1}$ |
| TAF1_ENSG00000147133 | 0.89 | $3.6 \times 10^{-1}$ |
| POLR1G_ENSG00000117877 | 0.81 | $2.5 \times 10^{-1}$ |
| TAF9B_ENSG00000187325 | 0.86 | $2.5 \times 10^{-1}$ |
| NELFE_ENSG00000204356 | 0.87 | $2.1 \times 10^{-1}$ |
| POLR2I_ENSG00000105258 | 0.82 | $2.0 \times 10^{-1}$ |
| H2AC7_ENSG00000196866 | 0.76 | $1.7 \times 10^{-1}$ |
| POLR2E_ENSG00000099817 | 0.83 | $1.6 \times 10^{-1}$ |
| GTF2A2_ENSG00000140307 | 0.82 | $1.3 \times 10^{-1}$ |
| MBD3_ENSG00000071655 | 0.86 | $1.3 \times 10^{-1}$ |
| ACTB_ENSG00000075624 | 0.81 | $1.2 \times 10^{-1}$ |
| POLR2G_ENSG00000168002 | 0.81 | $1.0 \times 10^{-1}$ |
| MLLT1_ENSG00000130382 | 0.82 | $8.4 \times 10^{-2}$ |
| H3C13_ENSG00000183598 | 0.67 | $8.0 \times 10^{-2}$ |
| GTF2E1_ENSG00000153767 | 0.82 | $7.7 \times 10^{-2}$ |
| IPO8_ENSG00000133704 | 0.84 | $5.9 \times 10^{-2}$ |
| SNRPD3_ENSG00000100028 | 0.79 | $5.0 \times 10^{-2}$ |
| H2AC20_ENSG00000184260 | 0.65 | $4.5 \times 10^{-2}$ |
| LSM10_ENSG00000181817 | 0.76 | $4.4 \times 10^{-2}$ |
| SSRP1_ENSG00000149136 | 0.79 | $4.0 \times 10^{-2}$ |
| H2BC21_ENSG00000184678 | 0.67 | $3.7 \times 10^{-2}$ |
| POLR2H_ENSG00000163882 | 0.77 | $3.1 \times 10^{-2}$ |
| KAT2A_ENSG00000108773 | 0.78 | $2.6 \times 10^{-2}$ |
| RBBP4_ENSG00000162521 | 0.84 | $2.6 \times 10^{-2}$ |
| H3C12_ENSG00000197153 | 0.64 | $2.5 \times 10^{-2}$ |
| H3C4_ENSG00000197409 | 0.62 | $2.3 \times 10^{-2}$ |
| GTF2A1_ENSG00000165417 | 0.75 | $1.9 \times 10^{-2}$ |
| MTA3_ENSG00000057935 | 0.8 | $1.5 \times 10^{-2}$ |
| TAF6_ENSG00000106290 | 0.76 | $1.1 \times 10^{-2}$ |
| EHMT2_ENSG00000204371 | 0.77 | $9.2 \times 10^{-3}$ |
| EAF2_ENSG00000145088 | 0.7 | $8.5 \times 10^{-3}$ |
| ERCC2_ENSG00000104884 | 0.73 | $7.7 \times 10^{-3}$ |
| GTF2H2_ENSG00000145736 | 0.64 | $6.4 \times 10^{-3}$ |
| GTF2E2_ENSG00000197265 | 0.78 | $5.6 \times 10^{-3}$ |
| ERCC6_ENSG00000225830 | 0.68 | $2.8 \times 10^{-3}$ |
| CSTF2T_ENSG00000177613 | 0.7 | $2.4 \times 10^{-3}$ |
| GTF2H1_ENSG00000110768 | 0.72 | $1.9 \times 10^{-3}$ |
| H2AC6_ENSG00000180573 | 0.57 | $1.8 \times 10^{-3}$ |
| MYBBP1A_ENSG00000132382 | 0.75 | $1.3 \times 10^{-3}$ |
| LSM11_ENSG00000155858 | 0.68 | $1.0 \times 10^{-3}$ |
| MNAT1_ENSG00000020426 | 0.7 | $5.4 \times 10^{-4}$ |
| TAF12_ENSG00000120656 | 0.65 | $3.8 \times 10^{-4}$ |
| GTF2H4_ENSG00000213780 | 0.65 | $5.9 \times 10^{-5}$ |
| GTF2H3_ENSG00000111358 | 0.63 | $2.3 \times 10^{-6}$ |
| NELFB_ENSG00000188986 | 0.55 | $2.2 \times 10^{-8}$ |
| CSTF3_ENSG00000176102 | 0.47 | $1.7 \times 10^{-9}$ |

Reactomes involved in the transcription pathway included mRNA 3'-end processing, RNA Polymerase II transcription termination, transport of mature mRNA derived from an intronless transcript,  RNA Polymerase II pre-transcription events, RNA Polymerase II transcription elongation, transcriptional regulation by small RNAs, and positive epigenetic regulation of rRNA expression.

**Table S3.** Expression of mRNA processing pathway group of genes in CD22E12$^{low}$ B-ALL patients.

| Gene | Fold Increase (CD22E12$^{low}$/All Others) | Wald Statistic (P-value) |
|---|---|---|
| SMN1_ENSG00000172062 | 1.64 | 1.6 x $10^{-6}$ |
| KHSRP_ENSG00000088247 | 1.53 | 1.5 x $10^{-5}$ |
| HNRNPD_ENSG00000138668 | 1.51 | 2.5 x $10^{-5}$ |
| DDX20_ENSG00000064703 | 1.57 | 3.5 x $10^{-5}$ |
| PRKCD_ENSG00000163932 | 1.55 | 3.7 x $10^{-5}$ |
| PRPF8_ENSG00000174231 | 1.46 | 5.8 x $10^{-5}$ |
| YWHAZ_ENSG00000164924 | 1.46 | 6.8 x $10^{-5}$ |
| EXOSC6_ENSG00000223496 | 1.65 | 7.8 x $10^{-5}$ |
| SET_ENSG00000119335 | 1.26 | 8.2 x $10^{-4}$ |
| DIS3_ENSG00000083520 | 1.54 | 8.8 x $10^{-4}$ |
| ZFP36L1_ENSG00000185650 | 1.74 | 9.7 x $10^{-4}$ |
| SF3B2_ENSG00000087365 | 1.27 | 1.2 x $10^{-3}$ |
| EXOSC9_ENSG00000123737 | 1.36 | 1.4 x $10^{-3}$ |
| ZRSR2_ENSG00000169249 | 1.31 | 3.7 x $10^{-3}$ |
| YBX1_ENSG00000065978 | 1.36 | 7.3 x $10^{-3}$ |
| SNRNP200_ENSG00000144028 | 1.23 | 1.3 x $10^{-2}$ |
| RPP30_ENSG00000148688 | 1.27 | 1.4 x $10^{-2}$ |
| RPP21_ENSG00000241370 | 1.36 | 1.7 x $10^{-2}$ |
| PHAX_ENSG00000164902 | 1.29 | 2.6 x $10^{-2}$ |
| HSPB1_ENSG00000106211 | 1.51 | 3.9 x $10^{-2}$ |
| SF3B6_ENSG00000115128 | 1.28 | 4.6 x $10^{-2}$ |
| SF3B5_ENSG00000169976 | 1.36 | 4.6 x $10^{-2}$ |
| ELAVL1_ENSG00000066044 | 1.21 | 5.2 x $10^{-2}$ |
| SNRNP40_ENSG00000060688 | 1.15 | 6.0 x $10^{-2}$ |
| TGS1_ENSG00000137574 | 1.19 | 6.8 x $10^{-2}$ |
| SF3B1_ENSG00000115524 | 1.26 | 7.3 x $10^{-2}$ |
| SNU13_ENSG00000100138 | 1.2 | 8.9 x $10^{-2}$ |
| SF3B4_ENSG00000143368 | 1.26 | 1.0 x $10^{-1}$ |
| XRN1_ENSG00000114127 | 1.23 | 2.1 x $10^{-1}$ |
| GEMIN4_ENSG00000179409 | 1.14 | 3.1 x $10^{-1}$ |
| DCP2_ENSG00000172795 | 1.12 | 3.3 x $10^{-1}$ |
| GEMIN2_ENSG00000092208 | 1.14 | 3.5 x $10^{-1}$ |
| POP7_ENSG00000172336 | 1.13 | 4.0 x $10^{-1}$ |
| DDX1_ENSG00000079785 | 1.08 | 4.0 x $10^{-1}$ |
| RTRAF_ENSG00000087302 | 1.08 | 4.2 x $10^{-1}$ |
| ZFP36_ENSG00000128016 | 1.13 | 4.6 x $10^{-1}$ |
| C2orf49_ENSG00000135974 | 1.09 | 4.7 x $10^{-1}$ |
| RPP14_ENSG00000163684 | 1.05 | 5.3 x $10^{-1}$ |
| RTCB_ENSG00000100220 | 1.06 | 5.5 x $10^{-1}$ |
| EXOSC8_ENSG00000120699 | 1.05 | 5.6 x $10^{-1}$ |
| ZCRB1_ENSG00000139168 | 1.07 | 5.9 x $10^{-1}$ |
| DDX42_ENSG00000198231 | 1.04 | 6.7 x $10^{-1}$ |

| | | |
|---|---|---|
| SNRPD2_ENSG00000125743 | 1.05 | $7.2 \times 10^{-1}$ |
| SNRNP35_ENSG00000184209 | 1.02 | $7.6 \times 10^{-1}$ |
| HSPA1B_ENSG00000204388 | 2.43 | $9.3 \times 10^{-1}$ |
| SNRPD1_ENSG00000167088 | 0.99 | $9.4 \times 10^{-1}$ |
| HSPA8_ENSG00000109971 | 0.98 | $8.9 \times 10^{-1}$ |
| FAM98B_ENSG00000171262 | 0.94 | $6.2 \times 10^{-1}$ |
| EXOSC2_ENSG00000130713 | 0.95 | $6.0 \times 10^{-1}$ |
| XPOT_ENSG00000184575 | 0.95 | $6.0 \times 10^{-1}$ |
| EFTUD2_ENSG00000108883 | 0.95 | $5.4 \times 10^{-1}$ |
| SF3B3_ENSG00000189091 | 0.95 | $5.3 \times 10^{-1}$ |
| TRNT1_ENSG00000072756 | 0.94 | $5.2 \times 10^{-1}$ |
| XPO1_ENSG00000082898 | 0.92 | $5.1 \times 10^{-1}$ |
| TXNL4A_ENSG00000141759 | 0.93 | $4.8 \times 10^{-1}$ |
| SNRNP25_ENSG00000161981 | 0.88 | $3.7 \times 10^{-1}$ |
| PRKCA_ENSG00000154229 | 0.84 | $3.5 \times 10^{-1}$ |
| HSPA1A_ENSG00000204389 | 2.76 | $2.5 \times 10^{-1}$ |
| ANP32A_ENSG00000140350 | 0.88 | $1.6 \times 10^{-1}$ |
| RPP40_ENSG00000124787 | 0.78 | $1.6 \times 10^{-1}$ |
| DDX23_ENSG00000174243 | 0.89 | $1.2 \times 10^{-1}$ |
| ELAC2_ENSG00000006744 | 0.89 | $1.2 \times 10^{-1}$ |
| PDCD7_ENSG00000090470 | 0.86 | $1.2 \times 10^{-1}$ |
| TSEN34_ENSG00000170892 | 0.84 | $1.1 \times 10^{-1}$ |
| ZBTB8OS_ENSG00000176261 | 0.86 | $1.1 \times 10^{-1}$ |
| PRPF6_ENSG00000101161 | 0.85 | $7.5 \times 10^{-2}$ |
| RPP38_ENSG00000152464 | 0.81 | $6.7 \times 10^{-2}$ |
| ZMAT5_ENSG00000100319 | 0.78 | $5.8 \times 10^{-2}$ |
| EXOSC4_ENSG00000178896 | 1 | $5.7 \times 10^{-2}$ |
| TSEN15_ENSG00000198860 | 0.75 | $3.1 \times 10^{-2}$ |
| GEMIN6_ENSG00000152147 | 0.71 | $2.2 \times 10^{-2}$ |
| SNUPN_ENSG00000169371 | 0.82 | $2.2 \times 10^{-2}$ |
| EXOSC7_ENSG00000075914 | 0.86 | $2.0 \times 10^{-2}$ |
| SNRNP48_ENSG00000168566 | 0.8 | $2.0 \times 10^{-2}$ |
| POP4_ENSG00000105171 | 0.79 | $2.0 \times 10^{-2}$ |
| RPP25_ENSG00000178718 | 0.58 | $1.3 \times 10^{-2}$ |
| TSEN54_ENSG00000182173 | 0.73 | $1.2 \times 10^{-2}$ |
| RNPC3_ENSG00000185946 | 0.68 | $9.3 \times 10^{-3}$ |
| AKT1_ENSG00000142208 | 0.76 | $8.9 \times 10^{-3}$ |
| LSM2_ENSG00000204392 | 0.67 | $8.8 \times 10^{-3}$ |
| PRMT5_ENSG00000100462 | 0.71 | $6.9 \times 10^{-3}$ |
| TNFSF13_ENSG00000161955 | 0.66 | $4.2 \times 10^{-3}$ |
| GEMIN8_ENSG00000046647 | 0.73 | $3.0 \times 10^{-3}$ |
| EXOSC3_ENSG00000107371 | 0.68 | $4.2 \times 10^{-4}$ |
| EXOSC1_ENSG00000171311 | 0.72 | $2.7 \times 10^{-4}$ |
| EXOSC5_ENSG00000077348 | 0.49 | $1.9 \times 10^{-4}$ |
| POP1_ENSG00000104356 | 0.66 | $1.6 \times 10^{-4}$ |
| GEMIN5_ENSG00000082516 | 0.65 | $1.3 \times 10^{-4}$ |
| GEMIN7_ENSG00000142252 | 0.65 | $8.1 \times 10^{-5}$ |

| | | |
|---|---|---|
| POP5_ENSG00000167272 | 0.52 | $5.5 \times 10^{-5}$ |
| CLNS1A_ENSG00000074201 | 0.61 | $2.1 \times 10^{-5}$ |
| TSEN2_ENSG00000154743 | 0.53 | $1.2 \times 10^{-5}$ |
| PARN_ENSG00000140694 | 0.69 | $5.9 \times 10^{-6}$ |
| WDR77_ENSG00000116455 | 0.52 | $7.4 \times 10^{-7}$ |

Reactomes involved in mRNA processing pathway included regulation of mRNA stability by proteins that bind AU-rich elements; mRNA Splicing - minor pathway; tRNA processing in the nucleus and metabolism of non-coding RNA.

**Table S4.** Expression of mRNA_transport pathway group of genes in CD22E12$^{low}$ B-ALL patients.

| Gene | Fold Increase (CD22E12$^{low}$/All Others) | Wald Statistic (P-value) |
|---|---|---|
| RAE1_ENSG00000101146 | 1.89 | $1.1 \times 10^{-10}$ |
| POLDIP3_ENSG00000100227 | 1.62 | $3.8 \times 10^{-8}$ |
| SRRM1_ENSG00000133226 | 1.69 | $4.3 \times 10^{-8}$ |
| SRSF3_ENSG00000112081 | 1.69 | $1.6 \times 10^{-7}$ |
| NUP153_ENSG00000124789 | 2.1 | $2.0 \times 10^{-7}$ |
| NUP50_ENSG00000093000 | 1.76 | $3.3 \times 10^{-7}$ |
| FYTTD1_ENSG00000122068 | 1.88 | $5.0 \times 10^{-7}$ |
| RNPS1_ENSG00000205937 | 1.53 | $5.3 \times 10^{-7}$ |
| SRSF2_ENSG00000161547 | 1.57 | $6.4 \times 10^{-7}$ |
| SARNP_ENSG00000205323 | 1.64 | $7.0 \times 10^{-7}$ |
| SLBP_ENSG00000163950 | 1.93 | $1.1 \times 10^{-6}$ |
| NXF1_ENSG00000162231 | 1.69 | $1.8 \times 10^{-6}$ |
| DDX39B_ENSG00000198563 | 1.47 | $8.6 \times 10^{-6}$ |
| POM121_ENSG00000196313 | 1.61 | $1.0 \times 10^{-5}$ |
| NUP214_ENSG00000126883 | 1.42 | $1.2 \times 10^{-5}$ |
| NUP54_ENSG00000138750 | 1.65 | $1.5 \times 10^{-5}$ |
| NUP58_ENSG00000139496 | 1.7 | $3.6 \times 10^{-5}$ |
| SRSF5_ENSG00000100650 | 1.5 | $1.1 \times 10^{-4}$ |
| WDR33_ENSG00000136709 | 1.36 | $2.8 \times 10^{-4}$ |
| RANBP2_ENSG00000153201 | 1.89 | $3.8 \times 10^{-4}$ |
| ALYREF_ENSG00000183684 | 1.51 | $4.1 \times 10^{-4}$ |
| SRSF6_ENSG00000124193 | 1.46 | $5.2 \times 10^{-4}$ |
| DDX39A_ENSG00000123136 | 1.48 | $5.6 \times 10^{-4}$ |
| SRSF4_ENSG00000116350 | 1.51 | $6.9 \times 10^{-4}$ |
| MAGOH_ENSG00000162385 | 1.34 | $8.2 \times 10^{-4}$ |
| ZC3H11A_ENSG00000058673 | 1.57 | $1.0 \times 10^{-3}$ |
| SRSF7_ENSG00000115875 | 1.38 | $1.3 \times 10^{-3}$ |
| CDC40_ENSG00000168438 | 1.5 | $1.4 \times 10^{-3}$ |
| U2AF2_ENSG00000063244 | 1.25 | $3.2 \times 10^{-3}$ |
| NUP155_ENSG00000113569 | 1.41 | $3.3 \times 10^{-3}$ |
| SRSF11_ENSG00000116754 | 1.5 | $6.8 \times 10^{-3}$ |
| EIF4A3_ENSG00000141543 | 1.52 | $7.7 \times 10^{-3}$ |
| NUP98_ENSG00000110713 | 1.37 | $8.5 \times 10^{-3}$ |
| FIP1L1_ENSG00000145216 | 1.23 | $1.3 \times 10^{-2}$ |
| NUP88_ENSG00000108559 | 1.29 | $1.6 \times 10^{-2}$ |
| SEC13_ENSG00000157020 | 1.19 | $2.0 \times 10^{-2}$ |
| SRSF9_ENSG00000111786 | 1.2 | $2.1 \times 10^{-2}$ |
| NUP133_ENSG00000069248 | 1.25 | $3.8 \times 10^{-2}$ |
| SLU7_ENSG00000164609 | 1.26 | $5.4 \times 10^{-2}$ |
| U2AF1_ENSG00000160201 | 1.31 | $6.0 \times 10^{-2}$ |
| THOC1_ENSG00000079134 | 1.33 | $6.2 \times 10^{-2}$ |
| NXT1_ENSG00000132661 | 1.3 | $7.8 \times 10^{-2}$ |

| | | |
|---|---|---|
| CHTOP_ENSG00000160679 | 1.16 | $1.1 \times 10^{-1}$ |
| U2AF1L4_ENSG00000161265 | 1.19 | $1.2 \times 10^{-1}$ |
| TPR_ENSG00000047410 | 1.21 | $1.4 \times 10^{-1}$ |
| DHX38_ENSG00000140829 | 1.15 | $2.1 \times 10^{-1}$ |
| NUP188_ENSG00000095319 | 1.07 | $3.2 \times 10^{-1}$ |
| THOC2_ENSG00000125676 | 1.1 | $4.8 \times 10^{-1}$ |
| NCBP1_ENSG00000136937 | 1.09 | $5.2 \times 10^{-1}$ |
| NUP93_ENSG00000102900 | 1.05 | $5.4 \times 10^{-1}$ |
| SEH1L_ENSG00000085415 | 1.08 | $5.5 \times 10^{-1}$ |
| NUP160_ENSG00000030066 | 1.05 | $6.2 \times 10^{-1}$ |
| NUP62_ENSG00000213024 | 1.02 | $8.8 \times 10^{-1}$ |
| NUP107_ENSG00000111581 | 1.01 | $9.5 \times 10^{-1}$ |
| NCBP2_ENSG00000114503 | 1 | $9.7 \times 10^{-1}$ |
| CASC3_ENSG00000108349 | 1 | $9.8 \times 10^{-1}$ |
| CPSF3_ENSG00000119203 | 1 | $9.6 \times 10^{-1}$ |
| UPF3B_ENSG00000125351 | 0.93 | $4.8 \times 10^{-1}$ |
| SRSF1_ENSG00000136450 | 0.93 | $4.8 \times 10^{-1}$ |
| NUP210_ENSG00000132182 | 0.89 | $4.8 \times 10^{-1}$ |
| NUP42_ENSG00000136243 | 0.94 | $4.7 \times 10^{-1}$ |
| NUP35_ENSG00000163002 | 0.93 | $4.7 \times 10^{-1}$ |
| SYMPK_ENSG00000125755 | 0.94 | $3.5 \times 10^{-1}$ |
| NUP205_ENSG00000155561 | 0.9 | $3.2 \times 10^{-1}$ |
| NUP85_ENSG00000125450 | 0.92 | $2.8 \times 10^{-1}$ |
| NDC1_ENSG00000058804 | 0.89 | $2.8 \times 10^{-1}$ |
| THOC6_ENSG00000131652 | 0.85 | $1.9 \times 10^{-1}$ |
| THOC7_ENSG00000163634 | 0.84 | $1.7 \times 10^{-1}$ |
| CPSF4_ENSG00000160917 | 0.9 | $1.7 \times 10^{-1}$ |
| CPSF1_ENSG00000071894 | 0.83 | $5.5 \times 10^{-2}$ |
| THOC3_ENSG00000051596 | 0.74 | $1.7 \times 10^{-2}$ |
| NUP43_ENSG00000120253 | 0.66 | $4.1 \times 10^{-4}$ |
| CPSF2_ENSG00000165934 | 0.68 | $2.6 \times 10^{-4}$ |
| MAGOHB_ENSG00000111196 | 0.65 | $9.9 \times 10^{-5}$ |
| GLE1_ENSG00000119392 | 0.68 | $8.0 \times 10^{-5}$ |
| AAAS_ENSG00000094914 | 0.59 | $1.2 \times 10^{-5}$ |
| NUP37_ENSG00000075188 | 0.53 | $2.3 \times 10^{-6}$ |
| THOC5_ENSG00000100296 | 0.68 | $1.0 \times 10^{-6}$ |

Reactomes involved in the mRNA transport pathway included transport of mature transcript to cytoplasm, transport of mature mRNA derived from an intron-containing transcript and transport of mature mRNAs derived from intronless transcripts.

**Table S5.** Expression of translation pathway group of genes in CD22E12$^{low}$ B-ALL patients.

| Gene | Fold Increase (CD22E12$^{low}$/All Others) | Wald Statistic (P-value) |
|---|---|---|
| EIF4H_ENSG00000106682 | 1.67 | 1.4 x $10^{-7}$ |
| EIF4E_ENSG00000151247 | 1.81 | 5.5 x $10^{-6}$ |
| EIF5_ENSG00000100664 | 1.7 | 5.0 x $10^{-5}$ |
| ETF1_ENSG00000120705 | 1.67 | 1.2 x $10^{-4}$ |
| GSPT1_ENSG00000103342 | 1.53 | 1.6 x $10^{-4}$ |
| EIF3J_ENSG00000104131 | 1.30 | 3.6 x $10^{-4}$ |
| RPL39_ENSG00000198918 | 1.74 | 4.8 x $10^{-4}$ |
| EIF4A2_ENSG00000156976 | 1.45 | 5.5 x $10^{-4}$ |
| EEF1A2_ENSG00000101210 | 1.8 | 6.0 x $10^{-4}$ |
| EIF3A_ENSG00000107581 | 1.46 | 8.8 x $10^{-4}$ |
| EIF4A1_ENSG00000161960 | 1.31 | 3.9 x $10^{-3}$ |
| RPS27_ENSG00000177954 | 1.48 | 7.8 x $10^{-3}$ |
| RPL36AL_ENSG00000165502 | 1.38 | 1.0 x $10^{-2}$ |
| EIF3H_ENSG00000147677 | 1.28 | 1.7 x $10^{-2}$ |
| EIF1AX_ENSG00000173674 | 1.35 | 1.8 x $10^{-2}$ |
| RPL10_ENSG00000147403 | 1.44 | 2.0 x $10^{-2}$ |
| RPL24_ENSG00000114391 | 1.34 | 2.0 x $10^{-2}$ |
| RPS26_ENSG00000197728 | 1.24 | 2.2 x $10^{-2}$ |
| RPS29_ENSG00000213741 | 1.43 | 3.1 x $10^{-2}$ |
| RPS4X_ENSG00000198034 | 1.37 | 3.6 x $10^{-2}$ |
| EEF2_ENSG00000167658 | 1.28 | 3.6 x $10^{-2}$ |
| EEF1A1_ENSG00000156508 | 1.28 | 4.0 x $10^{-2}$ |
| EIF3F_ENSG00000175390 | 1.28 | 4.3 x $10^{-2}$ |
| RPS7_ENSG00000171863 | 1.28 | 4.5 x $10^{-2}$ |
| RPS13_ENSG00000110700 | 1.33 | 4.5 x $10^{-2}$ |
| RPL23A_ENSG00000198242 | 1.25 | 4.8 x $10^{-2}$ |
| RPLP2_ENSG00000177600 | 1.36 | 5.2 x $10^{-2}$ |
| RPL38_ENSG00000172809 | 1.35 | 5.4 x $10^{-2}$ |
| RPS12_ENSG00000112306 | 1.34 | 5.9 x $10^{-2}$ |
| RPS9_ENSG00000170889 | 1.29 | 6.6 x $10^{-2}$ |
| RPL13A_ENSG00000142541 | 1.27 | 6.7 x $10^{-2}$ |
| RPL34_ENSG00000109475 | 1.3 | 7.0 x $10^{-2}$ |
| RPL6_ENSG00000089009 | 1.22 | 7.0 x $10^{-2}$ |
| RPL18_ENSG00000063177 | 1.28 | 7.3 x $10^{-2}$ |
| RPL4_ENSG00000174444 | 1.23 | 7.9 x $10^{-2}$ |
| RPL12_ENSG00000197958 | 1.3 | 7.9 x $10^{-2}$ |
| EIF3B_ENSG00000106263 | 1.17 | 8.3 x $10^{-2}$ |
| RPL10A_ENSG00000198755 | 1.3 | 8.6 x $10^{-2}$ |
| RPL28_ENSG00000108107 | 1.3 | 8.6 x $10^{-2}$ |
| UBA52_ENSG00000221983 | 1.29 | 8.7 x $10^{-2}$ |
| RPL35A_ENSG00000182899 | 1.28 | 9.2 x $10^{-2}$ |
| RPLP1_ENSG00000137818 | 1.31 | 9.2 x $10^{-2}$ |

| | | |
|---|---|---|
| RPS15A_ENSG00000134419 | 1.27 | 9.4 x $10^{-2}$ |
| RPL31_ENSG00000071082 | 1.29 | 9.4 x $10^{-2}$ |
| RPL8_ENSG00000161016 | 1.25 | 9.9 x $10^{-2}$ |
| RPL14_ENSG00000188846 | 1.24 | 1.0 x $10^{-1}$ |
| EEF1D_ENSG00000104529 | 1.17 | 1.0 x $10^{-1}$ |
| RPL5_ENSG00000122406 | 1.2 | 1.0 x $10^{-1}$ |
| RPS16_ENSG00000105193 | 1.27 | 1.1 x $10^{-1}$ |
| RPS25_ENSG00000118181 | 1.25 | 1.1 x $10^{-1}$ |
| RPL11_ENSG00000142676 | 1.26 | 1.1 x $10^{-1}$ |
| EIF3L_ENSG00000100129 | 1.19 | 1.1 x $10^{-1}$ |
| RPS14_ENSG00000164587 | 1.25 | 1.1 x $10^{-1}$ |
| RPL21_ENSG00000122026 | 1.21 | 1.2 x $10^{-1}$ |
| EIF3D_ENSG00000100353 | 1.15 | 1.2 x $10^{-1}$ |
| RPL37_ENSG00000145592 | 1.26 | 1.3 x $10^{-1}$ |
| RPL15_ENSG00000174748 | 1.19 | 1.3 x $10^{-1}$ |
| EIF4G1_ENSG00000114867 | 1.15 | 1.4 x $10^{-1}$ |
| RPS27A_ENSG00000143947 | 1.22 | 1.4 x $10^{-1}$ |
| RPL37A_ENSG00000197756 | 1.26 | 1.4 x $10^{-1}$ |
| RPS19_ENSG00000105372 | 1.23 | 1.5 x $10^{-1}$ |
| RPL35_ENSG00000136942 | 1.25 | 1.5 x $10^{-1}$ |
| RPS5_ENSG00000083845 | 1.23 | 1.5 x $10^{-1}$ |
| RPS17_ENSG00000182774 | 1.28 | 1.6 x $10^{-1}$ |
| RPL13_ENSG00000167526 | 1.23 | 1.6 x $10^{-1}$ |
| RPL19_ENSG00000108298 | 1.21 | 1.6 x $10^{-1}$ |
| RPS20_ENSG00000008988 | 1.23 | 1.6 x $10^{-1}$ |
| RPL32_ENSG00000144713 | 1.22 | 1.6 x $10^{-1}$ |
| RPL30_ENSG00000156482 | 1.23 | 1.7 x $10^{-1}$ |
| RPS28_ENSG00000233927 | 1.25 | 1.7 x $10^{-1}$ |
| RPL18A_ENSG00000105640 | 1.25 | 1.7 x $10^{-1}$ |
| RPL27_ENSG00000131469 | 1.23 | 1.7 x $10^{-1}$ |
| RPS8_ENSG00000142937 | 1.19 | 1.8 x $10^{-1}$ |
| EIF3M_ENSG00000149100 | 1.12 | 1.8 x $10^{-1}$ |
| RPS11_ENSG00000142534 | 1.22 | 1.8 x $10^{-1}$ |
| RPL9_ENSG00000163682 | 1.14 | 1.8 x $10^{-1}$ |
| RPL29_ENSG00000162244 | 1.23 | 1.8 x $10^{-1}$ |
| EEF1B2_ENSG00000114942 | 1.21 | 1.9 x $10^{-1}$ |
| RPL22L1_ENSG00000163584 | 1.2 | 2.0 x $10^{-1}$ |
| RPL3_ENSG00000100316 | 1.18 | 2.0 x $10^{-1}$ |
| EIF3G_ENSG00000130811 | 1.13 | 2.0 x $10^{-1}$ |
| RPS2_ENSG00000140988 | 1.2 | 2.2 x $10^{-1}$ |
| RPS21_ENSG00000171858 | 1.22 | 2.2 x $10^{-1}$ |
| RPL26_ENSG00000161970 | 1.19 | 2.2 x $10^{-1}$ |
| RPS23_ENSG00000186468 | 1.17 | 2.3 x $10^{-1}$ |
| RPL7A_ENSG00000148303 | 1.17 | 2.4 x $10^{-1}$ |
| RPL22_ENSG00000116251 | 1.13 | 2.5 x $10^{-1}$ |
| EIF4B_ENSG00000063046 | 1.08 | 2.5 x $10^{-1}$ |
| RPS6_ENSG00000137154 | 1.16 | 2.7 x $10^{-1}$ |

| | | |
|---|---|---|
| RPS10_ENSG00000124614 | 1.2 | 2.8 x $10^{-1}$ |
| RPL23_ENSG00000125691 | 1.16 | 2.8 x $10^{-1}$ |
| RPL17_ENSG00000265681 | 1.13 | 2.8 x $10^{-1}$ |
| RPS15_ENSG00000115268 | 1.19 | 2.8 x $10^{-1}$ |
| RPS24_ENSG00000138326 | 1.16 | 2.9 x $10^{-1}$ |
| EIF2S1_ENSG00000134001 | 1.11 | 3.0 x $10^{-1}$ |
| RPS3A_ENSG00000145425 | 1.11 | 3.4 x $10^{-1}$ |
| RPL36_ENSG00000130255 | 1.15 | 3.7 x $10^{-1}$ |
| RPL27A_ENSG00000166441 | 1.13 | 3.8 x $10^{-1}$ |
| RPS3_ENSG00000149273 | 1.12 | 4.0 x $10^{-1}$ |
| PABPC1_ENSG00000070756 | 1.09 | 4.0 x $10^{-1}$ |
| RPS18_ENSG00000231500 | 1.14 | 4.2 x $10^{-1}$ |
| EIF3K_ENSG00000178982 | 1.1 | 4.6 x $10^{-1}$ |
| RPLP0_ENSG00000089157 | 1.11 | 4.8 x $10^{-1}$ |
| RPL41_ENSG00000229117 | 1.07 | 5.9 x $10^{-1}$ |
| EIF2S3_ENSG00000130741 | 1.05 | 5.9 x $10^{-1}$ |
| EEF1G_ENSG00000254772 | 1.06 | 6.5 x $10^{-1}$ |
| RPSA_ENSG00000168028 | 1.06 | 6.7 x $10^{-1}$ |
| EIF3E_ENSG00000104408 | 1.04 | 7.5 x $10^{-1}$ |
| EIF3I_ENSG00000084623 | 1.03 | 7.8 x $10^{-1}$ |
| RPL26L1_ENSG00000037241 | 1.01 | 9.1 x $10^{-1}$ |
| RPL3L_ENSG00000140986 | 1.72 | 1.0 x $10^{0}$ |
| RPS4Y1_ENSG00000129824 | 0.98 | 9.3 x $10^{-1}$ |
| GSPT2_ENSG00000189369 | 0.95 | 7.6 x $10^{-1}$ |
| RPL36A_ENSG00000241343 | 0.96 | 7.5 x $10^{-1}$ |
| RPL7_ENSG00000147604 | 0.95 | 7.4 x $10^{-1}$ |
| RPL39L_ENSG00000163923 | 0.92 | 7.0 x $10^{-1}$ |
| EIF2S2_ENSG00000125977 | 0.94 | 5.5 x $10^{-1}$ |
| TRMT112_ENSG00000173113 | 0.91 | 5.0 x $10^{-1}$ |
| EIF3C_ENSG00000184110 | 0.88 | 2.3 x $10^{-1}$ |
| RPS27L_ENSG00000185088 | 0.67 | 3.4 x $10^{-3}$ |
| EIF4EBP1_ENSG00000187840 | 0.59 | 1.2 x $10^{-3}$ |
| APEH_ENSG00000164062 | 0.56 | 5.2 x $10^{-5}$ |
| N6AMT1_ENSG00000156239 | 0.36 | 2.5 x $10^{-9}$ |

Reactomes involved in the translation pathway included formation of a pool of free 40S subunits, eukaryotic translation elongation, peptide chain elongation, eukaryotic translation termination, ribosomal scanning and start codon recognition, translation initiation complex formation, activation of the mRNA upon binding of the cap-binding complex and eIFs, and subsequent binding to 43S, and the formation of the ternary complex, and subsequently, 43S complex. .

**Table S6.** Expression of post-translational protein modification pathway group of genes in CD22E12[low] B-ALL patients.

| Gene | Fold Increase (CD22E12[low]/All Others) | Wald Statistic (P-value) |
|---|---|---|
| RTF1_ENSG00000137815 | 1.79 | $1.7 \times 10^{-13}$ |
| CREBBP_ENSG00000005339 | 2.16 | $3.5 \times 10^{-11}$ |
| UBE2G1_ENSG00000132388 | 1.79 | $4.5 \times 10^{-11}$ |
| EP300_ENSG00000100393 | 2.08 | $7.0 \times 10^{-10}$ |
| SIN3A_ENSG00000169375 | 1.84 | $1.0 \times 10^{-8}$ |
| SUMO1_ENSG00000116030 | 1.66 | $1.6 \times 10^{-8}$ |
| UBE2N_ENSG00000177889 | 1.56 | $5.3 \times 10^{-8}$ |
| USP7_ENSG00000187555 | 1.95 | $1.0 \times 10^{-7}$ |
| WAC_ENSG00000095787 | 1.99 | $1.6 \times 10^{-7}$ |
| DERL1_ENSG00000136986 | 1.62 | $4.4 \times 10^{-7}$ |
| HNRNPC_ENSG00000092199 | 1.57 | $8.1 \times 10^{-7}$ |
| UBE2D3_ENSG00000109332 | 1.48 | $8.2 \times 10^{-7}$ |
| OTULIN_ENSG00000154124 | 1.8 | $6.9 \times 10^{-6}$ |
| PIAS1_ENSG00000033800 | 1.76 | $9.5 \times 10^{-6}$ |
| HNRNPK_ENSG00000165119 | 1.37 | $1.0 \times 10^{-5}$ |
| USP9X_ENSG00000124486 | 2.05 | $1.7 \times 10^{-5}$ |
| UBE2A_ENSG00000077721 | 1.61 | $2.4 \times 10^{-5}$ |
| UBC_ENSG00000150991 | 1.61 | $3.7 \times 10^{-5}$ |
| CTR9_ENSG00000198730 | 1.66 | $4.3 \times 10^{-5}$ |
| CBX4_ENSG00000141582 | 1.87 | $1.0 \times 10^{-4}$ |
| SMC5_ENSG00000198887 | 1.67 | $1.4 \times 10^{-4}$ |
| TOP1_ENSG00000198900 | 1.61 | $1.5 \times 10^{-4}$ |
| TOPORS_ENSG00000197579 | 1.71 | $1.7 \times 10^{-4}$ |
| UBE2K_ENSG00000078140 | 1.35 | $2.1 \times 10^{-4}$ |
| UBE2L3_ENSG00000185651 | 1.39 | $2.5 \times 10^{-4}$ |
| NOP58_ENSG00000055044 | 1.52 | $3.0 \times 10^{-4}$ |
| CDC73_ENSG00000134371 | 1.46 | $4.7 \times 10^{-4}$ |
| UBE2Q2_ENSG00000140367 | 1.51 | $4.8 \times 10^{-4}$ |
| ZNF350_ENSG00000256683 | 1.44 | $7.3 \times 10^{-4}$ |
| RNF40_ENSG00000103549 | 1.41 | $7.6 \times 10^{-4}$ |
| UBE2D2_ENSG00000131508 | 1.34 | $1.3 \times 10^{-3}$ |
| RNF168_ENSG00000163961 | 1.43 | $2.3 \times 10^{-3}$ |
| SAFB_ENSG00000160633 | 1.47 | $2.5 \times 10^{-3}$ |
| PPARGC1A_ENSG00000109819 | 4.44 | $4.7 \times 10^{-3}$ |
| ZNF131_ENSG00000172262 | 1.33 | $4.7 \times 10^{-3}$ |
| UBE2B_ENSG00000119048 | 1.3 | $5.9 \times 10^{-3}$ |
| PAF1_ENSG00000006712 | 1.31 | $6.2 \times 10^{-3}$ |
| UHRF2_ENSG00000147854 | 1.45 | $6.8 \times 10^{-3}$ |
| SUZ12_ENSG00000178691 | 1.35 | $7.5 \times 10^{-3}$ |
| HDAC2_ENSG00000196591 | 1.3 | $7.7 \times 10^{-3}$ |
| RNF152_ENSG00000176641 | 1.88 | $8.2 \times 10^{-3}$ |
| ING2_ENSG00000168556 | 1.39 | $1.2 \times 10^{-2}$ |

| | | |
|---|---|---|
| NPM1_ENSG00000181163 | 1.28 | 1.2 x $10^{-2}$ |
| UBE2D1_ENSG00000072401 | 1.5 | 1.4 x $10^{-2}$ |
| UBE2R2_ENSG00000107341 | 1.31 | 1.8 x $10^{-2}$ |
| VCP_ENSG00000165280 | 1.25 | 1.9 x $10^{-2}$ |
| TOP2B_ENSG00000077097 | 1.39 | 1.9 x $10^{-2}$ |
| UBE2H_ENSG00000186591 | 1.41 | 2.0 x $10^{-2}$ |
| NCOA1_ENSG00000084676 | 1.22 | 2.2 x $10^{-2}$ |
| BCL10_ENSG00000142867 | 1.29 | 2.5 x $10^{-2}$ |
| UBB_ENSG00000170315 | 1.32 | 3.6 x $10^{-2}$ |
| UBE2I_ENSG00000103275 | 1.24 | 3.7 x $10^{-2}$ |
| SELENOS_ENSG00000131871 | 1.24 | 4.0 x $10^{-2}$ |
| RNF2_ENSG00000121481 | 1.18 | 4.3 x $10^{-2}$ |
| TRIM28_ENSG00000130726 | 1.2 | 5.0 x $10^{-2}$ |
| UBE2Z_ENSG00000159202 | 1.17 | 6.9 x $10^{-2}$ |
| SUMO2_ENSG00000188612 | 1.21 | 8.4 x $10^{-2}$ |
| NSMCE3_ENSG00000185115 | 1.25 | 9.8 x $10^{-2}$ |
| BMI1_ENSG00000168283 | 1.25 | 1.1 x $10^{-1}$ |
| HIPK2_ENSG00000064393 | 1.25 | 1.3 x $10^{-1}$ |
| HLA-A_ENSG00000206503 | 1.21 | 1.5 x $10^{-1}$ |
| PCNA_ENSG00000132646 | 1.24 | 1.5 x $10^{-1}$ |
| WRN_ENSG00000165392 | 1.25 | 1.7 x $10^{-1}$ |
| SUMO3_ENSG00000184900 | 1.2 | 1.7 x $10^{-1}$ |
| UBE2S_ENSG00000108106 | 1.2 | 1.8 x $10^{-1}$ |
| PHC2_ENSG00000134686 | 1.16 | 1.9 x $10^{-1}$ |
| DDX5_ENSG00000108654 | 1.15 | 2.1 x $10^{-1}$ |
| PIAS2_ENSG00000078043 | 1.15 | 2.2 x $10^{-1}$ |
| CDCA8_ENSG00000134690 | 1.22 | 2.3 x $10^{-1}$ |
| UBA6_ENSG00000033178 | 1.19 | 2.4 x $10^{-1}$ |
| CBX8_ENSG00000141570 | 1.17 | 2.7 x $10^{-1}$ |
| UCHL3_ENSG00000118939 | 1.1 | 2.9 x $10^{-1}$ |
| VHL_ENSG00000134086 | 1.11 | 3.1 x $10^{-1}$ |
| LEO1_ENSG00000166477 | 1.13 | 3.3 x $10^{-1}$ |
| TDG_ENSG00000139372 | 1.09 | 3.7 x $10^{-1}$ |
| PEX12_ENSG00000108733 | 1.16 | 3.8 x $10^{-1}$ |
| PIAS4_ENSG00000105229 | 1.09 | 3.9 x $10^{-1}$ |
| PHC3_ENSG00000173889 | 1.1 | 4.2 x $10^{-1}$ |
| PHC1_ENSG00000111752 | 1.08 | 4.4 x $10^{-1}$ |
| NRIP1_ENSG00000180530 | 1.13 | 5.0 x $10^{-1}$ |
| SP100_ENSG00000067066 | 1.08 | 5.2 x $10^{-1}$ |
| CDC34_ENSG00000099804 | 1.08 | 5.2 x $10^{-1}$ |
| NCOR2_ENSG00000196498 | 1.07 | 5.4 x $10^{-1}$ |
| HDAC4_ENSG00000068024 | 1.1 | 5.4 x $10^{-1}$ |
| PEX2_ENSG00000164751 | 1.1 | 5.4 x $10^{-1}$ |
| RANGAP1_ENSG00000100401 | 1.06 | 5.4 x $10^{-1}$ |
| MRTFA_ENSG00000196588 | 1.06 | 5.5 x $10^{-1}$ |
| NCOA2_ENSG00000140396 | 1.07 | 6.0 x $10^{-1}$ |
| TOP2A_ENSG00000131747 | 1.09 | 6.0 x $10^{-1}$ |

| | | |
|---|---|---|
| EID3_ENSG00000255150 | 1.07 | $6.3 \times 10^{-1}$ |
| SCMH1_ENSG00000010803 | 1.03 | $7.5 \times 10^{-1}$ |
| DAXX_ENSG00000204209 | 1.04 | $7.5 \times 10^{-1}$ |
| HDAC1_ENSG00000116478 | 1.02 | $8.1 \times 10^{-1}$ |
| HLTF_ENSG00000071794 | 1 | $9.7 \times 10^{-1}$ |
| H4C11_ENSG00000197238 | 1 | $9.8 \times 10^{-1}$ |
| RRAGA_ENSG00000155876 | 0.99 | $9.6 \times 10^{-1}$ |
| CDKN2A_ENSG00000147889 | 0.93 | $7.5 \times 10^{-1}$ |
| UBE2G2_ENSG00000184787 | 0.97 | $7.2 \times 10^{-1}$ |
| BLM_ENSG00000197299 | 0.95 | $6.9 \times 10^{-1}$ |
| H4C3_ENSG00000197061 | 0.92 | $6.8 \times 10^{-1}$ |
| UBA1_ENSG00000130985 | 0.96 | $6.7 \times 10^{-1}$ |
| CHD3_ENSG00000170004 | 0.96 | $6.7 \times 10^{-1}$ |
| UBE2W_ENSG00000104343 | 0.94 | $6.5 \times 10^{-1}$ |
| RNF181_ENSG00000168894 | 0.94 | $6.1 \times 10^{-1}$ |
| PEX13_ENSG00000162928 | 0.95 | $6.1 \times 10^{-1}$ |
| SATB1_ENSG00000182568 | 0.94 | $5.6 \times 10^{-1}$ |
| INCENP_ENSG00000149503 | 0.92 | $5.6 \times 10^{-1}$ |
| H2BC4_ENSG00000180596 | 0.88 | $5.4 \times 10^{-1}$ |
| H2BC12_ENSG00000197903 | 0.89 | $4.9 \times 10^{-1}$ |
| AURKB_ENSG00000178999 | 0.89 | $4.8 \times 10^{-1}$ |
| UBE2E3_ENSG00000170035 | 0.86 | $4.6 \times 10^{-1}$ |
| UBE2E1_ENSG00000170142 | 0.94 | $4.6 \times 10^{-1}$ |
| HDAC7_ENSG00000061273 | 0.92 | $4.6 \times 10^{-1}$ |
| BIRC5_ENSG00000089685 | 0.87 | $4.0 \times 10^{-1}$ |
| RING1_ENSG00000204227 | 0.91 | $3.9 \times 10^{-1}$ |
| PIAS3_ENSG00000131788 | 0.93 | $3.8 \times 10^{-1}$ |
| SATB2_ENSG00000119042 | 0.49 | $3.4 \times 10^{-1}$ |
| MDM2_ENSG00000135679 | 0.85 | $3.0 \times 10^{-1}$ |
| RNF20_ENSG00000155827 | 0.92 | $2.8 \times 10^{-1}$ |
| HERC2_ENSG00000128731 | 0.88 | $2.6 \times 10^{-1}$ |
| PML_ENSG00000140464 | 0.9 | $2.4 \times 10^{-1}$ |
| H2BC5_ENSG00000158373 | 0.79 | $2.3 \times 10^{-1}$ |
| RAD18_ENSG00000070950 | 0.88 | $2.0 \times 10^{-1}$ |
| CETN2_ENSG00000147400 | 0.82 | $2.0 \times 10^{-1}$ |
| NSMCE4A_ENSG00000107672 | 0.89 | $1.5 \times 10^{-1}$ |
| RNF144A_ENSG00000151692 | 1.43 | $1.2 \times 10^{-1}$ |
| CBX2_ENSG00000173894 | 0.78 | $1.2 \times 10^{-1}$ |
| PARP1_ENSG00000143799 | 0.8 | $1.0 \times 10^{-1}$ |
| PARK7_ENSG00000116288 | 0.81 | $8.3 \times 10^{-2}$ |
| PEX14_ENSG00000142655 | 0.83 | $8.1 \times 10^{-2}$ |
| H4-16_ENSG00000197837 | 0.7 | $6.9 \times 10^{-2}$ |
| H2BC15_ENSG00000233822 | 0.69 | $6.4 \times 10^{-2}$ |
| AURKA_ENSG00000087586 | 0.72 | $5.7 \times 10^{-2}$ |
| H2BC11_ENSG00000124635 | 0.68 | $5.1 \times 10^{-2}$ |
| NSMCE1_ENSG00000169189 | 0.71 | $4.2 \times 10^{-2}$ |
| UBE2C_ENSG00000175063 | 0.69 | $3.9 \times 10^{-2}$ |

| | | |
|---|---|---|
| MDC1_ENSG00000137337 | 0.83 | $3.8 \times 10^{-2}$ |
| PRKDC_ENSG00000253729 | 0.76 | $3.6 \times 10^{-2}$ |
| H2BC13_ENSG00000185130 | 0.61 | $2.8 \times 10^{-2}$ |
| MBD1_ENSG00000141644 | 0.86 | $1.9 \times 10^{-2}$ |
| CBX5_ENSG00000094916 | 0.76 | $1.2 \times 10^{-2}$ |
| USP5_ENSG00000111667 | 0.76 | $8.2 \times 10^{-3}$ |
| SHPRH_ENSG00000146414 | 0.71 | $7.6 \times 10^{-3}$ |
| XPC_ENSG00000154767 | 0.77 | $6.9 \times 10^{-3}$ |
| UBE2T_ENSG00000077152 | 0.6 | $5.3 \times 10^{-3}$ |
| SMC6_ENSG00000163029 | 0.63 | $3.0 \times 10^{-3}$ |
| RAD52_ENSG00000002016 | 0.69 | $2.2 \times 10^{-3}$ |
| BRCA1_ENSG00000012048 | 0.63 | $1.7 \times 10^{-3}$ |
| H4C8_ENSG00000158406 | 0.55 | $1.5 \times 10^{-3}$ |
| NSMCE2_ENSG00000156831 | 0.77 | $1.3 \times 10^{-3}$ |
| CASP8AP2_ENSG00000118412 | 0.61 | $1.1 \times 10^{-3}$ |
| PEX10_ENSG00000157911 | 0.67 | $7.8 \times 10^{-4}$ |
| L3MBTL2_ENSG00000100395 | 0.73 | $3.3 \times 10^{-4}$ |
| WDR61_ENSG00000140395 | 0.64 | $2.8 \times 10^{-4}$ |
| ZBED1_ENSG00000214717 | 0.61 | $1.8 \times 10^{-4}$ |
| UBE2V2_ENSG00000169139 | 0.61 | $1.0 \times 10^{-4}$ |
| TRIM27_ENSG00000204713 | 0.69 | $6.7 \times 10^{-5}$ |
| DDX17_ENSG00000100201 | 0.58 | $6.0 \times 10^{-5}$ |
| XRCC4_ENSG00000152422 | 0.57 | $2.7 \times 10^{-5}$ |
| UBE2J2_ENSG00000160087 | 0.65 | $1.9 \times 10^{-5}$ |
| CTBP1_ENSG00000159692 | 0.62 | $9.1 \times 10^{-6}$ |
| RPA1_ENSG00000132383 | 0.53 | $1.6 \times 10^{-6}$ |
| PEX5_ENSG00000139197 | 0.6 | $4.2 \times 10^{-7}$ |
| TMEM129_ENSG00000168936 | 0.53 | $2.9 \times 10^{-8}$ |

Reactomes involved in the post-translational protein modification pathway involved synthesis of active ubiquitin, protein ubiquitination; SUMOylation of proteins, including RNA-binding proteins, DNA replication proteins, ubiquitinylation proteins, transcription cofactors, chromatin organization proteins and DNA damage response and repair proteins. .

**Table S7.** Expression of signal transduction pathway genes in CD22E12^low B-ALL patients.

| Gene | Fold Increase (CD22E12^low/All Others) | Wald Statistic (P-value) |
|---|---|---|
| PPP2CA_ENSG00000113575 | 1.91 | $3.3 \times 10^{-11}$ |
| PSMC2_ENSG00000161057 | 1.64 | $8.3 \times 10^{-10}$ |
| SKP1_ENSG00000113558 | 1.57 | $1.5 \times 10^{-8}$ |
| NFKB1_ENSG00000109320 | 2.32 | $2.1 \times 10^{-7}$ |
| NRAS_ENSG00000213281 | 1.62 | $5.2 \times 10^{-7}$ |
| RAF1_ENSG00000132155 | 1.66 | $6.6 \times 10^{-7}$ |
| TAB2_ENSG00000055208 | 1.85 | $6.7 \times 10^{-7}$ |
| PSMD11_ENSG00000108671 | 1.61 | $7.4 \times 10^{-7}$ |
| PSMD12_ENSG00000197170 | 1.95 | $1.2 \times 10^{-6}$ |
| CDC42_ENSG00000070831 | 1.65 | $1.2 \times 10^{-6}$ |
| CUL3_ENSG00000036257 | 1.86 | $2.1 \times 10^{-6}$ |
| RAC1_ENSG00000136238 | 1.54 | $2.1 \times 10^{-6}$ |
| MAPKAPK5_ENSG00000089022 | 1.3 | $5.4 \times 10^{-6}$ |
| DUSP4_ENSG00000120875 | 2.31 | $6 \times 10^{-6}$ |
| MARK3_ENSG00000075413 | 1.5 | $1.3 \times 10^{-5}$ |
| MAP2K3_ENSG00000034152 | 1.94 | $1.7 \times 10^{-5}$ |
| MAPKAPK2_ENSG00000162889 | 1.76 | $1.7 \times 10^{-5}$ |
| PPP1CB_ENSG00000213639 | 1.75 | $1.8 \times 10^{-5}$ |
| ATF2_ENSG00000115966 | 1.67 | $2.8 \times 10^{-5}$ |
| HSP90AA1_ENSG00000080824 | 1.68 | $3.1 \times 10^{-5}$ |
| PSMD6_ENSG00000163636 | 1.51 | $4.3 \times 10^{-5}$ |
| PPP1CC_ENSG00000186298 | 1.53 | $6.5 \times 10^{-5}$ |
| PSMD9_ENSG00000110801 | 1.46 | $7.5 \times 10^{-5}$ |
| CCT2_ENSG00000166226 | 1.4 | $8.7 \times 10^{-5}$ |
| CUL1_ENSG00000055130 | 1.35 | $2.8 \times 10^{-4}$ |
| SPRED2_ENSG00000198369 | 2.08 | $3.4 \times 10^{-4}$ |
| PSMD5_ENSG00000095261 | 1.52 | $3.5 \times 10^{-4}$ |
| CHUK_ENSG00000213341 | 1.57 | $3.6 \times 10^{-4}$ |
| MAPK6_ENSG00000069956 | 1.57 | $4.3 \times 10^{-4}$ |
| TXNL1_ENSG00000091164 | 1.38 | $4.6 \times 10^{-4}$ |
| TNIP2_ENSG00000168884 | 1.52 | $4.8 \times 10^{-4}$ |
| MAPK8_ENSG00000107643 | 1.62 | $5.4 \times 10^{-4}$ |
| SHOC2_ENSG00000108061 | 1.61 | $5.4 \times 10^{-4}$ |
| SEPTIN7_ENSG00000122545 | 1.42 | $6 \times 10^{-4}$ |
| PSMC6_ENSG00000100519 | 1.53 | $1 \times 10^{-3}$ |
| BRAF_ENSG00000157764 | 1.61 | $1.1 \times 10^{-3}$ |
| KRAS_ENSG00000133703 | 1.59 | $1.4 \times 10^{-3}$ |
| TWF1_ENSG00000151239 | 1.52 | $1.8 \times 10^{-3}$ |
| PPP2R5A_ENSG00000066027 | 1.46 | $1.9 \times 10^{-3}$ |
| MAP3K11_ENSG00000173327 | 1.42 | $2 \times 10^{-3}$ |
| PPP2R5E_ENSG00000154001 | 1.26 | $2.4 \times 10^{-3}$ |
| CDC42EP3_ENSG00000163171 | 1.9 | $2.6 \times 10^{-3}$ |

| | | |
|---|---|---|
| BRAP_ENSG00000089234 | 1.34 | $2.9 \times 10^{-3}$ |
| MAP2K7_ENSG00000076984 | 1.35 | $3.2 \times 10^{-3}$ |
| PPP2R1B_ENSG00000137713 | 1.41 | $3.5 \times 10^{-3}$ |
| TRA2B_ENSG00000136527 | 1.42 | $3.8 \times 10^{-3}$ |
| PSMD2_ENSG00000175166 | 1.23 | $9.3 \times 10^{-3}$ |
| TNRC6A_ENSG00000090905 | 1.41 | $1.2 \times 10^{-2}$ |
| CDC14A_ENSG00000079335 | 1.44 | $1.2 \times 10^{-2}$ |
| TNRC6C_ENSG00000078687 | 1.36 | $1.3 \times 10^{-2}$ |
| FBXW11_ENSG00000072803 | 1.32 | $1.4 \times 10^{-2}$ |
| PSME4_ENSG00000068878 | 1.49 | $1.4 \times 10^{-2}$ |
| IRAK2_ENSG00000134070 | 1.6 | $1.5 \times 10^{-2}$ |
| PSMD14_ENSG00000115233 | 1.22 | $1.7 \times 10^{-2}$ |
| HSP90AB1_ENSG00000096384 | 1.34 | $1.7 \times 10^{-2}$ |
| TRAF6_ENSG00000175104 | 1.31 | $1.8 \times 10^{-2}$ |
| ARAF_ENSG00000078061 | 1.25 | $2.1 \times 10^{-2}$ |
| PSMC1_ENSG00000100764 | 1.29 | $2.1 \times 10^{-2}$ |
| PSMA2_ENSG00000106588 | 1.19 | $2.2 \times 10^{-2}$ |
| DAB2IP_ENSG00000136848 | 1.66 | $2.6 \times 10^{-2}$ |
| MEF2A_ENSG00000068305 | 1.3 | $2.7 \times 10^{-2}$ |
| YWHAB_ENSG00000166913 | 1.23 | $2.7 \times 10^{-2}$ |
| IRAK1_ENSG00000184216 | 1.29 | $3.3 \times 10^{-2}$ |
| PSMA3_ENSG00000100567 | 1.18 | $3.4 \times 10^{-2}$ |
| FOXO1_ENSG00000150907 | 1.39 | $3.5 \times 10^{-2}$ |
| MRAS_ENSG00000158186 | 1.37 | $3.6 \times 10^{-2}$ |
| PSMB1_ENSG00000008018 | 1.22 | $3.9 \times 10^{-2}$ |
| RBMX_ENSG00000147274 | 1.15 | $4.1 \times 10^{-2}$ |
| PSMB7_ENSG00000136930 | 1.23 | $4.7 \times 10^{-2}$ |
| CAMK2A_ENSG00000070808 | 1.24 | $4.8 \times 10^{-2}$ |
| MAPK1_ENSG00000100030 | 1.19 | $5.6 \times 10^{-2}$ |
| ATF1_ENSG00000123268 | 1.27 | $5.7 \times 10^{-2}$ |
| MAP2K1_ENSG00000169032 | 1.3 | $6.4 \times 10^{-2}$ |
| NCOA3_ENSG00000124151 | 1.26 | $6.5 \times 10^{-2}$ |
| SPRED1_ENSG00000166068 | 1.51 | $6.6 \times 10^{-2}$ |
| DUSP7_ENSG00000164086 | 1.32 | $6.6 \times 10^{-2}$ |
| CALM1_ENSG00000198668 | 1.19 | $6.8 \times 10^{-2}$ |
| PSMD7_ENSG00000103035 | 1.17 | $8 \times 10^{-2}$ |
| ELK1_ENSG00000126767 | 1.25 | $8.3 \times 10^{-2}$ |
| PSMC4_ENSG00000013275 | 1.18 | $8.4 \times 10^{-2}$ |
| MSI2_ENSG00000153944 | 1.29 | $8.5 \times 10^{-2}$ |
| TAB3_ENSG00000157625 | 1.22 | $9.7 \times 10^{-2}$ |
| CAMK2D_ENSG00000145349 | 1.41 | $1 \times 10^{-1}$ |
| AGO2_ENSG00000123908 | 1.32 | $1.1 \times 10^{-1}$ |
| PSMC5_ENSG00000087191 | 1.14 | $1.1 \times 10^{-1}$ |
| SRC_ENSG00000197122 | 1.26 | $1.1 \times 10^{-1}$ |
| KBTBD7_ENSG00000120696 | 1.25 | $1.1 \times 10^{-1}$ |
| NOD2_ENSG00000167207 | 1.4 | $1.2 \times 10^{-1}$ |
| KSR1_ENSG00000141068 | 1.19 | $1.2 \times 10^{-1}$ |

| | | |
|---|---|---|
| PSMD1_ENSG00000173692 | 1.13 | $1.3 \times 10^{-1}$ |
| PSMA1_ENSG00000129084 | 1.1 | $1.6 \times 10^{-1}$ |
| PPP2R5C_ENSG00000078304 | 1.17 | $1.8 \times 10^{-1}$ |
| PSMB4_ENSG00000159377 | 1.12 | $1.9 \times 10^{-1}$ |
| DBN1_ENSG00000113758 | 1.19 | $2.4 \times 10^{-1}$ |
| ACTG1_ENSG00000184009 | 1.19 | $2.4 \times 10^{-1}$ |
| MYO6_ENSG00000196586 | 2.59 | $2.5 \times 10^{-1}$ |
| PHIP_ENSG00000146247 | 1.18 | $2.7 \times 10^{-1}$ |
| MAP3K8_ENSG00000107968 | 1.19 | $3 \times 10^{-1}$ |
| RBX1_ENSG00000100387 | 1.11 | $3.3 \times 10^{-1}$ |
| PEDS1-UBE2V1_ENSG00000124208 | 1.09 | $3.4 \times 10^{-1}$ |
| PSMA7_ENSG00000101182 | 1.09 | $3.7 \times 10^{-1}$ |
| SPRED3_ENSG00000188766 | 1.15 | $4.2 \times 10^{-1}$ |
| AGO3_ENSG00000126070 | 1.09 | $4.3 \times 10^{-1}$ |
| RIPK2_ENSG00000104312 | 1.13 | $4.3 \times 10^{-1}$ |
| RASAL2_ENSG00000075391 | 1.2 | $4.3 \times 10^{-1}$ |
| PSMC3_ENSG00000165916 | 1.08 | $4.4 \times 10^{-1}$ |
| PSMB2_ENSG00000126067 | 1.09 | $4.4 \times 10^{-1}$ |
| MAP2K4_ENSG00000065559 | 1.09 | $4.4 \times 10^{-1}$ |
| KALRN_ENSG00000160145 | 2.15 | $4.9 \times 10^{-1}$ |
| UBE2V1_ENSG00000244687 | 1.06 | $4.9 \times 10^{-1}$ |
| PAK1_ENSG00000149269 | 1.08 | $5.3 \times 10^{-1}$ |
| PPP2CB_ENSG00000104695 | 1.06 | $5.3 \times 10^{-1}$ |
| RASA2_ENSG00000155903 | 1.1 | $5.4 \times 10^{-1}$ |
| MAPK14_ENSG00000112062 | 1.08 | $5.7 \times 10^{-1}$ |
| CDC37_ENSG00000105401 | 1.05 | $5.9 \times 10^{-1}$ |
| MYC_ENSG00000136997 | 1.09 | $6 \times 10^{-1}$ |
| RPS6KA3_ENSG00000177189 | 1.08 | $6.2 \times 10^{-1}$ |
| DUSP6_ENSG00000139318 | 1.07 | $6.8 \times 10^{-1}$ |
| JUN_ENSG00000177606 | 1.06 | $7.2 \times 10^{-1}$ |
| CDK1_ENSG00000170312 | 1.05 | $7.8 \times 10^{-1}$ |
| DNAJB1_ENSG00000132002 | 2.3 | $7.9 \times 10^{-1}$ |
| PSMA8_ENSG00000154611 | 1.37 | $8.1 \times 10^{-1}$ |
| CDC42EP5_ENSG00000167617 | 1.05 | $8.4 \times 10^{-1}$ |
| RASAL1_ENSG00000111344 | 1.05 | $8.5 \times 10^{-1}$ |
| CCT6A_ENSG00000146731 | 1.02 | $8.5 \times 10^{-1}$ |
| MAPK7_ENSG00000166484 | 1.03 | $8.8 \times 10^{-1}$ |
| TNRC6B_ENSG00000100354 | 1 | $1 \times 10^{0}$ |
| SEM1_ENSG00000127922 | 1 | $1 \times 10^{0}$ |
| NF1_ENSG00000196712 | 1 | $9.9 \times 10^{-1}$ |
| PSMB6_ENSG00000142507 | 0.99 | $9.6 \times 10^{-1}$ |
| RPS6KA5_ENSG00000100784 | 0.99 | $9.3 \times 10^{-1}$ |
| HRAS_ENSG00000174775 | 0.99 | $9.2 \times 10^{-1}$ |
| PSMB8_ENSG00000204264 | 0.99 | $9.1 \times 10^{-1}$ |
| FOS_ENSG00000170345 | 0.98 | $9 \times 10^{-1}$ |
| PSME3_ENSG00000131467 | 0.96 | $7.1 \times 10^{-1}$ |
| PSME1_ENSG00000092010 | 0.95 | $6.8 \times 10^{-1}$ |

| | | |
|---|---|---|
| MAP2K2_ENSG00000126934 | 0.94 | 6.1 x $10^{-1}$ |
| FOXO3_ENSG00000118689 | 0.93 | 5.9 x $10^{-1}$ |
| ACTN1_ENSG00000072110 | 0.91 | 5.7 x $10^{-1}$ |
| STK38_ENSG00000112079 | 0.93 | 5.4 x $10^{-1}$ |
| PRKACA_ENSG00000072062 | 0.93 | 5.3 x $10^{-1}$ |
| PPP2R5B_ENSG00000068971 | 0.93 | 5.2 x $10^{-1}$ |
| JAK2_ENSG00000096968 | 0.91 | 4.7 x $10^{-1}$ |
| PSMD8_ENSG00000099341 | 0.91 | 4.6 x $10^{-1}$ |
| MOV10_ENSG00000155363 | 0.93 | 4.2 x $10^{-1}$ |
| TMOD3_ENSG00000138594 | 0.92 | 4 x $10^{-1}$ |
| MEF2C_ENSG00000081189 | 0.88 | 3.8 x $10^{-1}$ |
| RASA3_ENSG00000185989 | 0.88 | 3.7 x $10^{-1}$ |
| CAMK2B_ENSG00000058404 | 1.3 | 3.5 x $10^{-1}$ |
| MAP2K6_ENSG00000108984 | 0.85 | 3.3 x $10^{-1}$ |
| MAPK11_ENSG00000185386 | 0.84 | 3.2 x $10^{-1}$ |
| RPS6KA2_ENSG00000071242 | 0.82 | 3.2 x $10^{-1}$ |
| ETV4_ENSG00000175832 | 0.67 | 3.1 x $10^{-1}$ |
| PSMF1_ENSG00000125818 | 0.88 | 2.6 x $10^{-1}$ |
| RASAL3_ENSG00000105122 | 0.89 | 2.3 x $10^{-1}$ |
| CCND3_ENSG00000112576 | 0.83 | 2.1 x $10^{-1}$ |
| SYNGAP1_ENSG00000197283 | 0.85 | 2.1 x $10^{-1}$ |
| PSMA6_ENSG00000100902 | 0.87 | 2.1 x $10^{-1}$ |
| PPP2R1A_ENSG00000105568 | 0.86 | 1.8 x $10^{-1}$ |
| RASA4_ENSG00000105808 | 0.84 | 1.6 x $10^{-1}$ |
| PSMD10_ENSG00000101843 | 0.83 | 1.6 x $10^{-1}$ |
| CDC42EP2_ENSG00000149798 | 0.78 | 1.4 x $10^{-1}$ |
| NOD1_ENSG00000106100 | 0.81 | 1.3 x $10^{-1}$ |
| PSMB5_ENSG00000100804 | 0.84 | 1.2 x $10^{-1}$ |
| PSME2_ENSG00000100911 | 0.84 | 1.1 x $10^{-1}$ |
| BTRC_ENSG00000166167 | 0.82 | 1 x $10^{-1}$ |
| PSMD3_ENSG00000108344 | 0.88 | 1 x $10^{-1}$ |
| PSMD13_ENSG00000185627 | 0.87 | 8.4 x $10^{-2}$ |
| MAPK10_ENSG00000109339 | 0.83 | 7.9 x $10^{-2}$ |
| PAK2_ENSG00000180370 | 0.86 | 7.6 x $10^{-2}$ |
| CALM3_ENSG00000160014 | 0.8 | 7.6 x $10^{-2}$ |
| AGO4_ENSG00000134698 | 0.84 | 7.3 x $10^{-2}$ |
| CREB1_ENSG00000118260 | 0.85 | 6.6 x $10^{-2}$ |
| CDC14B_ENSG00000081377 | 0.32 | 6.6 x $10^{-2}$ |
| MAPK9_ENSG00000050748 | 0.88 | 6.1 x $10^{-2}$ |
| PSMA4_ENSG00000041357 | 0.83 | 5.1 x $10^{-2}$ |
| PPP2R5D_ENSG00000112640 | 0.83 | 5 x $10^{-2}$ |
| CALM2_ENSG00000143933 | 0.81 | 4.6 x $10^{-2}$ |
| RAG2_ENSG00000175097 | 0.65 | 4.2 x $10^{-2}$ |
| VRK3_ENSG00000105053 | 0.82 | 2.6 x $10^{-2}$ |
| RAG1_ENSG00000166349 | 0.6 | 2.2 x $10^{-2}$ |
| MAP3K7_ENSG00000135341 | 0.8 | 1.4 x $10^{-2}$ |
| PSMA5_ENSG00000143106 | 0.76 | 6.4 x $10^{-3}$ |

| | | |
|---|---|---|
| PHB_ENSG00000167085 | 0.66 | $4.7 \times 10^{-3}$ |
| CCT7_ENSG00000135624 | 0.68 | $3.4 \times 10^{-3}$ |
| AGO1_ENSG00000092847 | 0.78 | $2.5 \times 10^{-3}$ |
| RASA1_ENSG00000145715 | 0.69 | $2.3 \times 10^{-3}$ |
| PSMB10_ENSG00000205220 | 0.65 | $1.8 \times 10^{-3}$ |
| MAPK3_ENSG00000102882 | 0.7 | $1.2 \times 10^{-3}$ |
| IKBKB_ENSG00000104365 | 0.71 | $4.2 \times 10^{-4}$ |
| CAMK2G_ENSG00000148660 | 0.68 | $3.4 \times 10^{-4}$ |
| DUSP3_ENSG00000108861 | 0.59 | $3.2 \times 10^{-4}$ |
| IGF2BP1_ENSG00000159217 | 0.76 | $2.4 \times 10^{-4}$ |
| MAPKAPK3_ENSG00000114738 | 0.62 | $1.4 \times 10^{-4}$ |
| RPS6KA1_ENSG00000117676 | 0.63 | $1.1 \times 10^{-4}$ |
| PRKACB_ENSG00000142875 | 0.49 | $2.8 \times 10^{-5}$ |
| PSMD4_ENSG00000159352 | 0.59 | $7.1 \times 10^{-6}$ |
| RHOBTB2_ENSG00000008853 | 0.44 | $5.7 \times 10^{-6}$ |
| TAB1_ENSG00000100324 | 0.68 | $5.2 \times 10^{-6}$ |
| PSMB9_ENSG00000240065 | 0.48 | $3.4 \times 10^{-6}$ |

Reactomes involved in signal transduction included RAF activation, MAP kinase activation, RHOBTB2, GTPase cycle, regulation of RAS by GAPs and MAPK6/MAPK4 signaling. .

**Table S8.** Expression of cell cycle pathway genes in CD22E12$^{low}$ B-ALL patients.

| Gene | Fold Increase (CD22E12$^{low}$/All Others) | Wald Statistic (P-value) |
| --- | --- | --- |
| DYNC1LI1_ENSG00000144635 | 1.69 | 5.5 x $10^{-10}$ |
| MAPRE1_ENSG00000101367 | 1.76 | 7.2 x $10^{-8}$ |
| FZR1_ENSG00000105325 | 1.98 | 1.2 x $10^{-6}$ |
| CHMP4B_ENSG00000101421 | 1.79 | 6.6 x $10^{-6}$ |
| PAFAH1B1_ENSG00000007168 | 1.72 | 7.5 x $10^{-6}$ |
| RCC2_ENSG00000179051 | 1.52 | 1.0 x $10^{-5}$ |
| CHMP2B_ENSG00000083937 | 1.55 | 3.5 x $10^{-5}$ |
| B9D2_ENSG00000123810 | 1.81 | 5.3 x $10^{-5}$ |
| CENPN_ENSG00000166451 | 1.6 | 6.0 x $10^{-5}$ |
| CNEP1R1_ENSG00000205423 | 1.52 | 1.7 x $10^{-4}$ |
| CDC27_ENSG00000004897 | 1.63 | 1.9 x $10^{-4}$ |
| TUBB3_ENSG00000258947 | 2.39 | 2.2 x $10^{-4}$ |
| CENPL_ENSG00000120334 | 1.54 | 2.3 x $10^{-4}$ |
| FBXO5_ENSG00000112029 | 1.55 | 2.5 x $10^{-4}$ |
| IST1_ENSG00000182149 | 1.67 | 3.2 x $10^{-4}$ |
| CENPU_ENSG00000151725 | 1.69 | 3.6 x $10^{-4}$ |
| BUB3_ENSG00000154473 | 1.33 | 6.1 x $10^{-4}$ |
| AHCTF1_ENSG00000153207 | 1.85 | 6.8 x $10^{-4}$ |
| NIPBL_ENSG00000164190 | 1.63 | 9.8 x $10^{-4}$ |
| WAPL_ENSG00000062650 | 1.7 | 1.0 x $10^{-3}$ |
| RAD21_ENSG00000164754 | 1.43 | 1.1 x $10^{-3}$ |
| TUBB4B_ENSG00000188229 | 1.59 | 1.2 x $10^{-3}$ |
| STAG2_ENSG00000101972 | 1.56 | 1.8 x $10^{-3}$ |
| PPP2R2A_ENSG00000221914 | 1.44 | 2.2 x $10^{-3}$ |
| LMNA_ENSG00000160789 | 1.99 | 2.6 x $10^{-3}$ |
| STAG1_ENSG00000118007 | 1.38 | 4.3 x $10^{-3}$ |
| PMF1_ENSG00000160783 | 1.35 | 9.9 x $10^{-3}$ |
| RAN_ENSG00000132341 | 1.29 | 1.0 x $10^{-2}$ |
| ESCO1_ENSG00000141446 | 1.37 | 1.3 x $10^{-2}$ |
| LPIN1_ENSG00000134324 | 1.35 | 2.1 x $10^{-2}$ |
| CENPO_ENSG00000138092 | 1.32 | 2.3 x $10^{-2}$ |
| ERCC6L_ENSG00000186871 | 1.5 | 2.3 x $10^{-2}$ |
| MAU2_ENSG00000129933 | 1.2 | 2.4 x $10^{-2}$ |
| LBR_ENSG00000143815 | 1.29 | 2.5 x $10^{-2}$ |
| PMF1-BGLAP_ENSG00000260238 | 1.31 | 2.5 x $10^{-2}$ |
| OPA1_ENSG00000198836 | 1.33 | 2.6 x $10^{-2}$ |
| PDS5B_ENSG00000083642 | 1.32 | 2.8 x $10^{-2}$ |
| RNF103-CHMP3_ENSG00000249884 | 1.32 | 3.3 x $10^{-2}$ |
| TNPO1_ENSG00000083312 | 1.27 | 3.7 x $10^{-2}$ |
| ANKLE2_ENSG00000176915 | 1.2 | 5.6 x $10^{-2}$ |
| TUBB2A_ENSG00000137267 | 1.46 | 5.6 x $10^{-2}$ |
| TUBB4A_ENSG00000104833 | 1.57 | 7.0 x $10^{-2}$ |

| | | |
|---|---|---|
| NUF2_ENSG00000143228 | 1.35 | 7.6 x $10^{-2}$ |
| PDS5A_ENSG00000121892 | 1.18 | 8.3 x $10^{-2}$ |
| CLASP1_ENSG00000074054 | 1.21 | 9.2 x $10^{-2}$ |
| ARHGAP10_ENSG00000071205 | 1.38 | 9.5 x $10^{-2}$ |
| NDE1_ENSG00000072864 | 1.22 | 9.8 x $10^{-2}$ |
| KNL1_ENSG00000137812 | 1.33 | 1.0 x $10^{-1}$ |
| TUBB6_ENSG00000176014 | 1.44 | 1.1 x $10^{-1}$ |
| KPNB1_ENSG00000108424 | 1.13 | 1.5 x $10^{-1}$ |
| TUBB2B_ENSG00000137285 | 0.53 | 1.6 x $10^{-1}$ |
| EMD_ENSG00000102119 | 1.17 | 1.9 x $10^{-1}$ |
| CKAP5_ENSG00000175216 | 1.18 | 1.9 x $10^{-1}$ |
| CLIP1_ENSG00000130779 | 1.17 | 1.9 x $10^{-1}$ |
| LEMD3_ENSG00000174106 | 1.15 | 2.2 x $10^{-1}$ |
| DYNC1H1_ENSG00000197102 | 1.18 | 2.3 x $10^{-1}$ |
| CDCA5_ENSG00000146670 | 1.21 | 2.4 x $10^{-1}$ |
| LPIN2_ENSG00000101577 | 1.14 | 2.6 x $10^{-1}$ |
| DYNLL2_ENSG00000264364 | 1.18 | 2.8 x $10^{-1}$ |
| TMPO_ENSG00000120802 | 1.12 | 2.8 x $10^{-1}$ |
| SMC3_ENSG00000108055 | 1.13 | 3.0 x $10^{-1}$ |
| TUBA1C_ENSG00000167553 | 1.15 | 3.0 x $10^{-1}$ |
| ANAPC10_ENSG00000164162 | 1.12 | 3.1 x $10^{-1}$ |
| CHMP7_ENSG00000147457 | 1.14 | 3.3 x $10^{-1}$ |
| NDEL1_ENSG00000166579 | 1.1 | 3.9 x $10^{-1}$ |
| NDC80_ENSG00000080986 | 1.12 | 3.9 x $10^{-1}$ |
| BUB1B_ENSG00000156970 | 1.14 | 4.1 x $10^{-1}$ |
| DYNC1LI2_ENSG00000135720 | 1.13 | 4.7 x $10^{-1}$ |
| CENPC_ENSG00000145241 | 1.11 | 4.8 x $10^{-1}$ |
| CLASP2_ENSG00000163539 | 1.09 | 5.2 x $10^{-1}$ |
| KIF18A_ENSG00000121621 | 1.11 | 5.2 x $10^{-1}$ |
| TUBB1_ENSG00000101162 | 1.14 | 5.4 x $10^{-1}$ |
| NEK7_ENSG00000151414 | 1.08 | 5.5 x $10^{-1}$ |
| KIF23_ENSG00000137807 | 1.1 | 5.5 x $10^{-1}$ |
| ESCO2_ENSG00000171320 | 1.09 | 6.0 x $10^{-1}$ |
| KIF2A_ENSG00000068796 | 1.06 | 6.0 x $10^{-1}$ |
| CHMP2A_ENSG00000130724 | 1.05 | 6.1 x $10^{-1}$ |
| LMNB1_ENSG00000113368 | 1.07 | 6.2 x $10^{-1}$ |
| NEK2_ENSG00000117650 | 1.07 | 6.8 x $10^{-1}$ |
| SGO1_ENSG00000129810 | 1.06 | 6.9 x $10^{-1}$ |
| TAOK1_ENSG00000160551 | 1.06 | 7.0 x $10^{-1}$ |
| CHMP3_ENSG00000115561 | 1.03 | 7.1 x $10^{-1}$ |
| CENPF_ENSG00000117724 | 1.07 | 7.1 x $10^{-1}$ |
| MAD1L1_ENSG00000002822 | 1.03 | 7.9 x $10^{-1}$ |
| CTDNEP1_ENSG00000175826 | 1.03 | 8.0 x $10^{-1}$ |
| ANAPC2_ENSG00000176248 | 1.02 | 8.2 x $10^{-1}$ |
| ANAPC15_ENSG00000110200 | 1.03 | 8.3 x $10^{-1}$ |
| CCNA2_ENSG00000145386 | 1.02 | 9.0 x $10^{-1}$ |
| SIRT2_ENSG00000068903 | 1.01 | 9.0 x $10^{-1}$ |

| | | |
|---|---|---|
| CENPH_ENSG00000153044 | 1.02 | $9.1 \times 10^{-1}$ |
| CENPE_ENSG00000138778 | 1.01 | $9.4 \times 10^{-1}$ |
| PTTG1_ENSG00000164611 | 1.01 | $9.7 \times 10^{-1}$ |
| TUBA1A_ENSG00000167552 | 1 | $9.8 \times 10^{-1}$ |
| KIF2C_ENSG00000142945 | 0.96 | $8.1 \times 10^{-1}$ |
| KNTC1_ENSG00000184445 | 0.96 | $8.0 \times 10^{-1}$ |
| LEMD2_ENSG00000161904 | 0.97 | $7.9 \times 10^{-1}$ |
| VRK1_ENSG00000100749 | 0.97 | $7.8 \times 10^{-1}$ |
| MIS12_ENSG00000167842 | 0.97 | $7.7 \times 10^{-1}$ |
| RCC1_ENSG00000180198 | 0.97 | $7.6 \times 10^{-1}$ |
| TUBA1B_ENSG00000123416 | 0.95 | $7.5 \times 10^{-1}$ |
| ZW10_ENSG00000086827 | 0.96 | $7.0 \times 10^{-1}$ |
| SKA1_ENSG00000154839 | 0.94 | $7.0 \times 10^{-1}$ |
| SKP2_ENSG00000145604 | 0.96 | $6.9 \times 10^{-1}$ |
| KIF20A_ENSG00000112984 | 0.93 | $6.8 \times 10^{-1}$ |
| MAD2L1_ENSG00000164109 | 0.92 | $5.6 \times 10^{-1}$ |
| CDK2_ENSG00000123374 | 0.94 | $5.6 \times 10^{-1}$ |
| SPAST_ENSG00000021574 | 0.94 | $4.5 \times 10^{-1}$ |
| TUBA8_ENSG00000183785 | 0.85 | $3.9 \times 10^{-1}$ |
| NUDC_ENSG00000090273 | 0.92 | $3.8 \times 10^{-1}$ |
| ANAPC11_ENSG00000141552 | 0.87 | $3.6 \times 10^{-1}$ |
| CDC20_ENSG00000117399 | 0.83 | $3.1 \times 10^{-1}$ |
| TUBA4A_ENSG00000127824 | 0.84 | $2.6 \times 10^{-1}$ |
| CENPS-CORT_ENSG00000251503 | 0.85 | $2.5 \times 10^{-1}$ |
| VPS4A_ENSG00000132612 | 0.92 | $2.2 \times 10^{-1}$ |
| SPC24_ENSG00000161888 | 0.81 | $1.8 \times 10^{-1}$ |
| CCNB1_ENSG00000134057 | 0.81 | $1.7 \times 10^{-1}$ |
| CENPS_ENSG00000175279 | 0.82 | $1.7 \times 10^{-1}$ |
| CCNB2_ENSG00000157456 | 0.78 | $1.4 \times 10^{-1}$ |
| PLK1_ENSG00000166851 | 0.78 | $1.4 \times 10^{-1}$ |
| BUB1_ENSG00000169679 | 0.8 | $1.3 \times 10^{-1}$ |
| ZWINT_ENSG00000122952 | 0.78 | $1.2 \times 10^{-1}$ |
| CENPT_ENSG00000102901 | 0.88 | $1.1 \times 10^{-1}$ |
| CENPI_ENSG00000102384 | 0.77 | $9.3 \times 10^{-2}$ |
| ANAPC4_ENSG00000053900 | 0.79 | $8.4 \times 10^{-2}$ |
| NEK6_ENSG00000119408 | 0.76 | $6.5 \times 10^{-2}$ |
| SMC1A_ENSG00000072501 | 0.83 | $5.9 \times 10^{-2}$ |
| DSN1_ENSG00000149636 | 0.81 | $5.9 \times 10^{-2}$ |
| DYNLL1_ENSG00000088986 | 0.73 | $5.3 \times 10^{-2}$ |
| RB1_ENSG00000139687 | 0.78 | $4.4 \times 10^{-2}$ |
| CENPM_ENSG00000100162 | 0.7 | $4.1 \times 10^{-2}$ |
| CENPA_ENSG00000115163 | 0.72 | $3.4 \times 10^{-2}$ |
| SPC25_ENSG00000152253 | 0.68 | $3.2 \times 10^{-2}$ |
| ANAPC1_ENSG00000153107 | 0.85 | $3.1 \times 10^{-2}$ |
| SKA2_ENSG00000182628 | 0.76 | $3.1 \times 10^{-2}$ |
| BANF1_ENSG00000175334 | 0.69 | $2.5 \times 10^{-2}$ |
| SPDL1_ENSG00000040275 | 0.75 | $2.0 \times 10^{-2}$ |

| | | |
|---|---|---|
| ANAPC5_ENSG00000089053 | 0.82 | 1.9 x $10^{-2}$ |
| NSL1_ENSG00000117697 | 0.8 | 1.6 x $10^{-2}$ |
| LPIN3_ENSG00000132793 | 0.78 | 1.1 x $10^{-2}$ |
| ZWILCH_ENSG00000174442 | 0.76 | 9.9 x $10^{-3}$ |
| SGO2_ENSG00000163535 | 0.67 | 8.2 x $10^{-3}$ |
| VRK2_ENSG00000028116 | 0.77 | 7.1 x $10^{-3}$ |
| CDC23_ENSG00000094880 | 0.78 | 6.3 x $10^{-3}$ |
| CENPP_ENSG00000188312 | 0.73 | 5.6 x $10^{-3}$ |
| CENPQ_ENSG00000031691 | 0.69 | 5.6 x $10^{-3}$ |
| CDC26_ENSG00000176386 | 0.73 | 5.2 x $10^{-3}$ |
| DYNC1I1_ENSG00000158560 | 0.93 | 4.1 x $10^{-3}$ |
| ANAPC7_ENSG00000196510 | 0.76 | 3.3 x $10^{-3}$ |
| PRKCB_ENSG00000166501 | 0.64 | 2.5 x $10^{-3}$ |
| ITGB3BP_ENSG00000142856 | 0.7 | 2.1 x $10^{-3}$ |
| DYNC1I2_ENSG00000077380 | 0.75 | 2.0 x $10^{-3}$ |
| CC2D1B_ENSG00000154222 | 0.74 | 3.8 x $10^{-4}$ |
| CDC16_ENSG00000130177 | 0.63 | 3.2 x $10^{-4}$ |
| ANAPC16_ENSG00000166295 | 0.61 | 3.0 x $10^{-4}$ |
| OMA1_ENSG00000162600 | 0.53 | 1.7 x $10^{-5}$ |
| CCNA1_ENSG00000133101 | 0.61 | 1.0 x $10^{-5}$ |
| CHMP4A_ENSG00000254505 | 0.65 | 6.3 x $10^{-6}$ |
| CENPK_ENSG00000123219 | 0.54 | 3.0 x $10^{-6}$ |
| NEK9_ENSG00000119638 | 0.49 | 1.7 x $10^{-8}$ |
| CHMP6_ENSG00000176108 | 0.45 | 1.3 x $10^{-11}$ |

Reactomes involved in the cell cycle pathway included postmitotic nuclear pore complex (NPC) reformation, nuclear envelope (NE) reassembly, mitotic telophase/cytokinesis, regulation of cell cycle linking apoptosis and proliferation, establishment of sister chromatid cohesion; nuclear pore complex (NPC) disassembly, nuclear envelope breakdown, amplification of signal from unattached kinetochores via a MAD2 inhibitory signal, amplification of signal from the kinetochores, APC/C-mediated degradation of cell cycle proteins and regulation of mitotic cell cycle.

**Table S9.** Comparison of the clinical features of the 141-patient RNAseq subset with those for the the total TARGET B-ALL patient population.

| Variable | RNA seq dataset (N=141) Mean/Median(Range) or N (% evaluable) | | TARGET B-ALL patients (N=1267) Mean/Median(Range) or N (% evaluable) | | P-value Mann-Whitney U Test or Fisher's Exact |
|---|---|---|---|---|---|
| **Age (yrs)** Mean±SEM / Median (Range) | 7.9 ± 0.5 | 6.5 (1.2 - 30) | 8.4 ± 0.2 (N=1126) | 6.9 (1 - 26.2) | 0.2 |
| **WBC (x10$^9$/L)** Mean±SEM / Median (Range) | 72.2 ± 10.4 | 28.3 (1.1 - 1149) | 63.7 ± 3.2 (N=1126) | 24.1 (0.4 - 1306) | 0.046 |
| **MRD at Day 8** Mean±SEM / Median (Range) | 5.1 ± 1.7 (N=40) | 1.1 (0 - 61.2) | 3.2 ± 0.5 (N=271) | 0.5 (0 - 84.6) | 0.016 |
| **MRD at Day 29** Mean±SEM / Median (Range) | 0.8 ± 0.3 | 0 (0 - 26) | 1.2 ± 0.2 (N=1119) | 0 (0 - 87.8) | 0.004 |
| **MRD at End of Consolidation** Mean±SEM / Median (Range) | 1.4 ± 1.2 (N=26) | 0 (0 - 31.5) | 0.5 ± 0.4 (N=117) | 0 (0 - 48.5) | 0.9 |
| **Induction Failure (N=140)** | 2/140(1.4%) | | 22/1260(1.7%) | | 1 |
| **Age category** Adult (≥ 18 yrs) | 5/141(3.5%) | | 41/1267(3.2%) | | 0.8 |
| **CNS Status at Diagnosis** CNS 2 + CNS 3 | 28/141(19.9%) | | 147/1267(11.6%) | | 0.003 (Odds Ratio =2.1) |
| **CNS site of relapse** | 15/141(10.6%) | | 64/1267(5.1%) | | 0.003 (Odds Ratio = 2.6) |
| **NCI Risk** High Risk | 90/141(63.8%) | | 855/1267(65.8%) | | 0.3 |
| **Age risk** Poor (Age <2 yrs or ≥10 years) | 64/141(45.3%) | | 620/1267(48.9%) | | 0.4 |
| **WBC category** ≥20x10$^9$/L | 83/141(58.9%) | | 690/1267(54.4%) | | 0.3 |
| **MRD at Day 8** MRD>0 | 40/40(100%) | | 288/311(92.6%) | | 0.055 |
| **MRD at Day 29** MRD>0 | 73/141(51.8%) | | 530/1267(41.8%) | | 0.014 (Odds Ratio = 1.6) |

| | | | |
|---|---|---|---|
| **MRD at Consolidation** | | | |
| MRD>0 | 8/26(30.8%) | 49/143(34.3%) | 0.8 |
| **Cytogenetics** | | | |
| Pseudodiploid | 43/106(40.1%) | 335/901(37.2%) | 0.5 |
| Pseudodiploid + Hypodiploid or Hyperdiploid with SCA | 78/106(73.6%) | 580/901(64.4%) | 0.04 |
| | | | (Odds Ratio = 1.6) |
| **Molecular Markers/FISH** | | | |
| BCR-ABL1 | 5/132(3.8%) | 38/774(4.9%) | 0.7 |
| MLL rearranged | 4/132(3%) | 21/774(2.7%) | 0.8 |
| TCF3-PBX1 | 14/132(10.6%) | 56/774(7.2%) | 0.14 |
| Hyperdiploid with Trisomy of chromosomes 4 and 10 | 10/132(7.6%) | 53/774(6.8%) | 0.7 |
| ETV6-RUNX1 | 11/132(8.3%) | 76/774(9.8%) | 0.6 |
| ETV6-RUNX1 + Trisomy of chromosomes 4 and 10 | 21/132(15.9%) | 129/774(16.7%) | 0.9 |
